# Synthesis of Benzimidazole-1,2,4-triazole Derivatives as Potential Antifungal Agents Targeting 14α-Demethylase

**DOI:** 10.1021/acsomega.2c07755

**Published:** 2023-01-19

**Authors:** Emir Güzel, Ulviye Acar Çevik, Asaf Evrim Evren, Hayrani Eren Bostancı, Ülküye Dudu Gül, Uğur Kayış, Yusuf Özkay, Zafer Asım Kaplancıklı

**Affiliations:** † Department of Pharmaceutical Chemistry, Faculty of Pharmacy, Biruni University, İstanbul 34010 Turkey; ‡ Department of Pharmaceutical Chemistry, Faculty of Pharmacy, Anadolu University, Eskişehir 26470, Turkey; § Department of Pharmacy Services, Vocational School of Health Services, Bilecik Şeyh Edebali University, 11000 Bilecik, Turkey; ∥ Department of Biochemistry, Faculty of Pharmacy, Sivas Cumhuriyet University, Sivas 58140, Turkey; ⊥ Department of Bioengineering, Faculty of Engineering, Bilecik Seyh Edebali University, Bilecik 11230, Turkey; # Pazaryeri Vocational School, Program of Pharmacy Services, Bilecik Şey Edebali University, 11230 Bilecik, Turkey

## Abstract

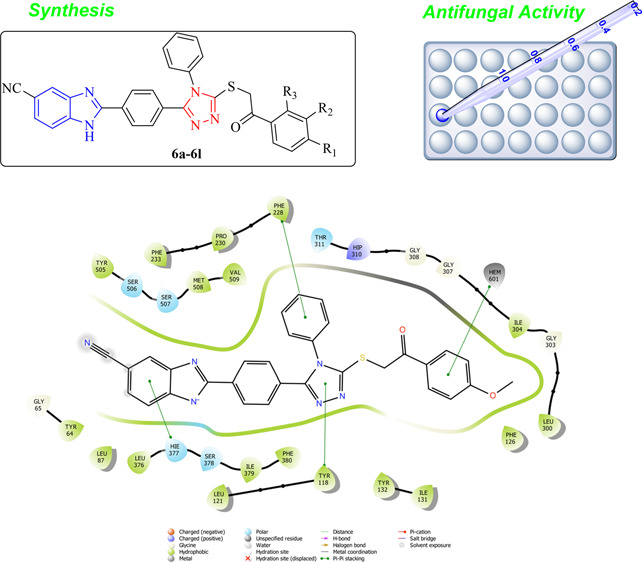

Invasive fungal infections (IFIs) are increasing as major infectious diseases around the world, and the limited efficacy of existing medications has resulted in substantial morbidity and death in patients due to the lack of effective antifungal agents and serious drug resistance. In this study, a series of benzimidazole-1,2,4-triazole derivatives (**6a**–**6l**) were synthesized and characterized by ^1^H NMR, ^13^C NMR, and HR-MS spectral analysis. All the target compounds were screened for their *in vitro* antifungal activity against four fungal strains, namely, *C. albicans*, *C. glabrata*, *C. krusei*, and *C. parapsilopsis*. The synthesized compounds exhibited significant antifungal potential, especially against *C. glabrata*. Three compounds (**6b**, **6i**, and **6j**) showed higher antifungal activity with their MIC values (0.97 μg/mL) compared with voriconazole and fluconazole. Molecular docking provided a possible binding mode of compounds **6b**, **6i**, and **6j** in the 14α-demethylase active site. Our studies suggested that the benzimidazole-1,2,4-triazole derivatives can be used as a new fungicidal lead targeting 14α-demethylase for further structural optimization. In addition, their effects on the L929 cell line were also investigated to evaluate the cytotoxic effects of the compounds. SEM analyses were performed to examine the effects of compounds **6a**, **6i**, and **6j** on *C. glabrata* cells under *in vivo* experimental conditions.

## 1 Introduction

Invasive fungal infection has become a growing threat with high morbidity and mortality due to the widespread use of glucocorticoids, broad-spectrum antimicrobials, and AIDS chemotherapy drugs, as well as the use of invasive procedures such as hemodialysis, deep vein catheterization, and transplantation. According to estimates, 1.5–2 million individuals die each year as a result of fungal diseases.^1−3^ At present, five chemical classes of compounds are employed for the treatment and prevention of fungal diseases worldwide: azoles (Fluconazole, Miconazole, Albaconazole, VT1161, etc.), polyenes (Amphotericin B and Nystatin), acrylamines (Butenafine, Naftifine, Terbinafine, etc.), antimetabolites (5-fluorocytosine), and echinocandins.^4−7^ However, the current therapeutic medications face significant obstacles, such as drug toxicity, drug–drug interactions, and drug resistance. That is why it is critical to synthesize effective and safe novel antifungal medications that are both.^8,9^

**Figure 1:**
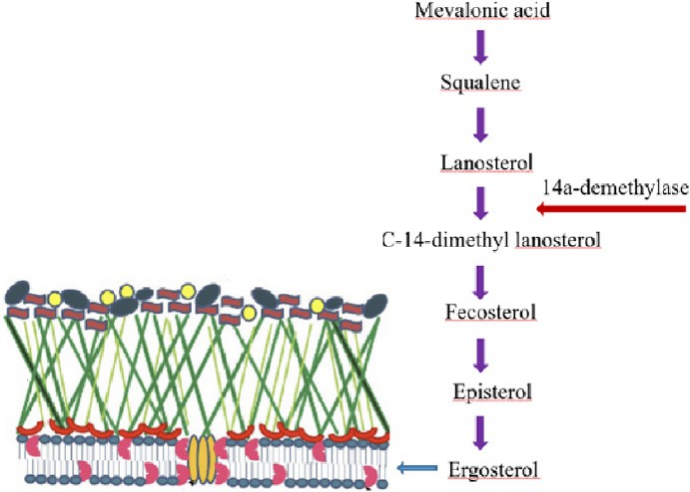
Predictive mechanism of potential antifungal compounds.

Ergosterol is structurally distinct from the sterols found in mammals and plants, and it serves a vital function in modulating the permeability and fluidity of fungal cell membranes.^10^ The cytochrome P450 enzyme lanosterol 14α-demethylase (CYP51) is a prominent target for the treatment of fungal infection. Azole drugs operate as fungistatic agents by reducing the action of CYP51, which prevents fungi from producing ergosterol.^11−13^ Azole antifungal drugs inhibit 14-DM by binding as the sixth ligand to the heme iron in the enzyme’s active site, changing the shape of the active site and functioning as non-competitive inhibitors. The affinity of the N-1 substituent for the cytochrome protein as well as the strength of the binding to heme iron determines the efficiency of azoles.^14^ When ergosterol biosynthesis is inhibited, fungal cell membranes are rapidly degraded, resulting in the suppression or death of harmful fungus.^15−17^ The predictive mechanism of potential antifungal compounds is shown in Figure 1.

**Figure 2:**
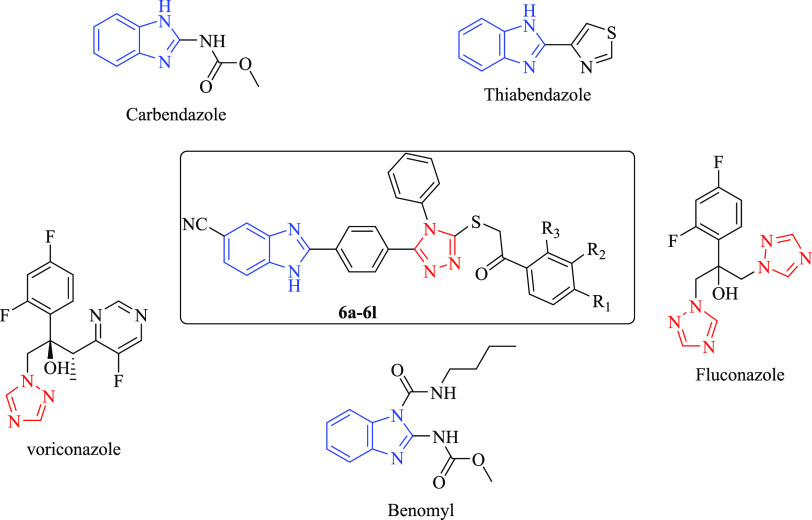
Antifungal drugs with benzimidazole and triazole structure and chemical structure of the designed compounds.

For a long time, benzimidazole derivatives as strong and safe antifungal medicines have drawn increasing interest among the pharmaceuticals used to treat fungal infections. Carbendazole, benomyl, and thiabendazole have been frequently used among them.^18−20^ Triazoles, a type of heterocyclic molecule, are favored scaffolds in a variety of fields. Fluconazole, itraconazole, and voriconazole are antifungal medicines available for therapeutic use that contain 1,2,4-triazole fragments.^21−24^ In recent years, many studies have been conducted on the antifungal activities of triazoles.^25−28^ Antifungal drugs with benzimidazole and triazole structures and the chemical structure of the designed compounds are shown in Figure 2.

**Figure 3:**
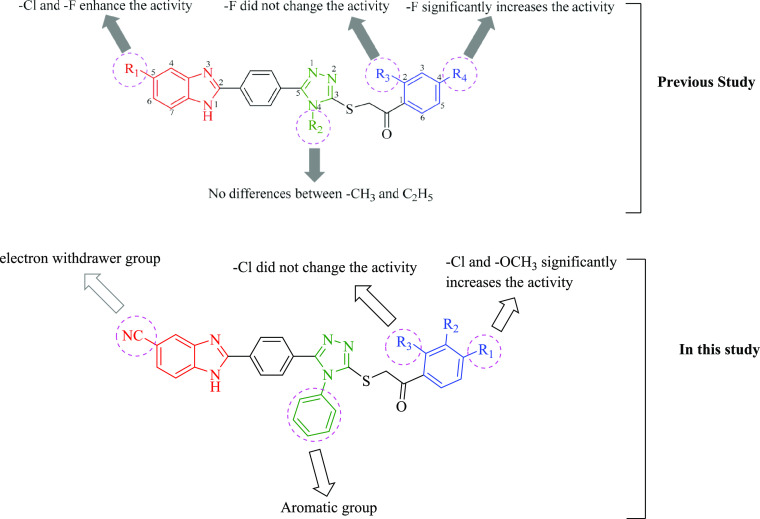
Design of synthesized compounds containing the benzimidazole-triazole structure.

In our previous research, we investigated the antifungal effects of compounds with a benzimidazole-1,2,4-triazole structure. The results of the study presented significant antifungal effects The structural variations of compounds can be classified in three regions (Figure 3). The first one is a benzimidazole ring carrying chloro or fluoro substituents at the C-5 position, or it is not substituted. The second region is a triazole ring in which there is a methyl or ethyl substituents at the N-4 position. The last region is a phenyl ring of the 1-phenyl-1-ethan-1-one substructure that carries different substituations. Looking at the chemical structure of the compounds that showed stronger anticandidal activity, they commonly bear fluoro substituents at the C-4 position of phenyl. Hence, it can be declared that C-4 of phenyl is a very important position in terms of anticandidal activity. The C-5 position of benzimidazole is essential, and fluoro or chloro substitution of this position significantly increases the antifungal activity. The methyl or ethyl substituents at the N-4 position of triazole did not cause a meaningful difference on biological activity.^29^

In the recent study, we synthesized the benzimidazole-triazole complex by replacing the methyl and ethyl group with a phenyl ring at the N-4 position of the triazole ring. The fifth position of the benzimidazole ring was substituted with the electron withdrawing −CN group (Figure 3). The structures of the synthesized derivatives were elucidated by ^1^H NMR, ^13^C NMR, and mass spectroscopic data. Then, we determined antifungal effects of the synthesized compounds by in vitro activity tests against four *Candida* species (*C. albicans*, *C. glabrata*, *C. krusei*, and *C. parapsilopsis*). At the active site of 14α-demethylase, molecular docking studies of compounds were performed.

## 2 Results and Discussion

### 2.1 Chemistry

**Scheme 1:**
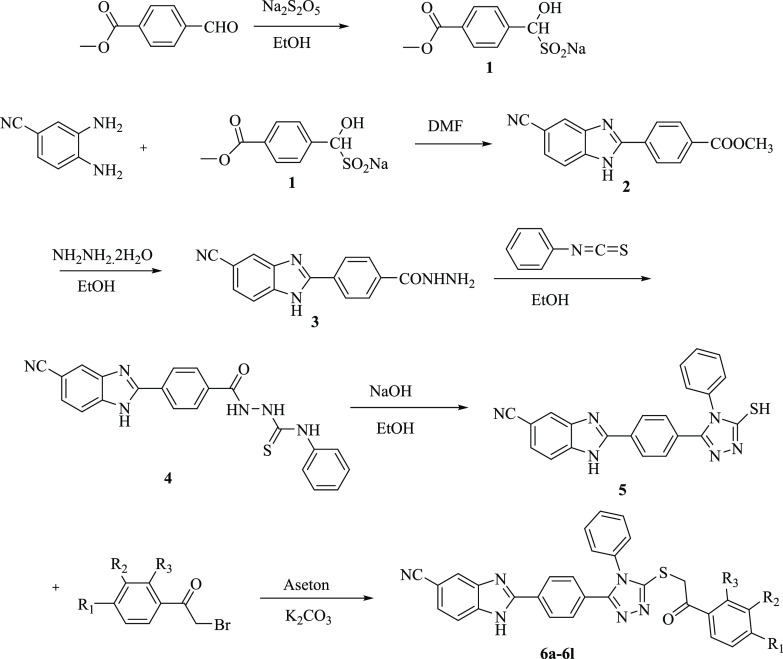
General Procedure for Synthesis of the Final Compounds **6a**–**6l**

The target molecules were synthesized in six steps as depicted in Scheme 1. First of all, the aldehyde part of the methyl 4-formylbenzoate compound was treated with sodium metabisulfite in ethanol to obtain the sodium metabisulfite addition product of the aldehyde. In the second step, as a result of the condensation reaction of the benzaldehyde sodium metabisulfite product and 5-cyano-1,2-phenylenediamine under reflux, methyl 4-(5-cyano-1*H*-benz[*d*]imidazole-2-yl)benzoate (**2**) was obtained. In the next step, compound **2** was treated with hydrazine hydrate in ethanol to obtain the 4-(5-cyano-1*H*-benz[*d*]imidazol-2-yl)benzohydrazide (**3**). The hydrazide derivative compound (**3**) was then refluxed with phenyl isothiocyanate, and precipitates were filtered. The compound (**4**) and NaOH in ethanol were refluxed, and 2-(4-(5-merkapto-4-fenil-4*H*-1,2,4-triazol-3-il)fenil)-1*H*-benz[*d*]imidazol-5-karbonitril (**5**) was obtained. The final reaction step was carried out between compound **5** and the appropriate 2-bromoacetophenone derivatives, and the target compounds (**6a**–**6l**) were obtained. The chemical structures of the compounds were shown in Table 1.

**Table 1:** Chemical Structure of the Synthesized Compounds **6a**–**6l**

–comp.	R_1_	R_2_	R_3_
**6a**	–NO_2_	–H	–H
**6b**	–OCH_3_	–H	–H
**6c**	–CN	–H	–H
**6d**	–F	–H	–H
**6e**	–phenyl	–H	–H
**6f**	–Br	–H	–H
**6g**	–Cl	–Cl	–H
**6h**	–F	–H	–F
**6i**	–Cl	–H	–H
**6j**	–Cl	–H	–Cl
**6k**	–H	–NO_2_	–H
**6l**	–H	–H	–H

The structures of the synthesized compounds were elucidated by ^1^H NMR, ^13^C NMR, and mass spectroscopy data. The methoxy group in the fourth position of the phenyl ring of compound **6b** was observed as a singlet at 3.84 ppm. The peaks of methylene (−CH_2_) protons, which are common to all of the compounds, were observed as singlets in the range of 4.87–5.02 ppm. The signals belonging to aromatic protons were found at 6.85–8.31 ppm. When the ^13^C NMR spectra of the compounds were examined, the carbon of methoxy belonging to the compound **6b** ring resonated at 56.16 ppm. Signals belonging to methylene substituent were detected at 29.40–31.14 ppm as a singlet. All masses were in accordance with the estimated M + H/2 values.

### 2.2 In Vitro Antifungal Activity

The antifungal activity of the target compounds **6a**–**6l** was evaluated by an in vitro method against *C. albicans*, *C. glabrata*, *C. krusei*, and *C. parapsilopsis*. The results are listed in Table 2. When the antifungal effects of the compounds were examined, it was found that all the compounds in the series were effective against *C. glabrata*. All synthesized compounds **6a**–**6l** also showed antifungal activity comparable to reference drugs with MIC_50_ values of 0.97–1.95 μg/mL. Especially, compounds **6b**, **6i**, and **6j** were the most effective compounds in the series with an MIC value of 0.97 μg/mL. These compounds were found to be two times more effective than the reference drug voriconazole and four times more effective than fluconazole. In the series, compounds **6a**, **6c**, **6d**, **6e**, **6f**, **6g**, **6h**, **6k**, and **6l** showed the same activity as voriconazole, while they were found to be two times more effective than fluconazole.

**Table 2:** Antifungal Activity Data of Synthesized Compounds and Reference Drugs **6a**–**6l** (μg/mL)

comp.	*C. albicans*	*C. krusei*	*C. glabrata*	*C. parapsilosis*
**6a**	31.25	125	1.95	62.5
**6b**	31.25	125	0.97	31.25
**6c**	31.25	125	1.95	31.25
**6d**	62.5	125	1.95	125
**6e**	62.5	125	1.95	62.5
**6f**	62.5	125	1.95	62.5
**6g**	125	250	1.95	31.25
**6h**	62.5	62.5	1.95	62.5
**6i**	62.5	125	0.97	31.25
**6j**	62.5	125	0.97	31.25
**6k**	31.25	125	1.95	31.25
**6l**	31.25	125	1.95	31.25
voriconazole	3.90	3.90	1.95	3.90
fluconazole	7.81	7.81	3.90	3.90

The differences in the chemical structures and the antibacterial activity profiles of compounds directed us to discuss structure activity relationships (SARs) (Figure 3). In our previous study, the fifth position of the benzimidazole ring is designed as chloro, fluoro, and non-substituted.^29^ According to the antifungal activity results, it was suggested that the C-5 position of benzimidazole is essential and fluoro or chloro substitution of this position significantly increases the antifungal activity. Therefore, in this study, instead of non-substituted benzimidazole, the electron-withdrawing group, the cyano group, was used. In the previous study, it was found that the methyl or ethyl substituents at the N-4 position of the triazole did not cause a significant difference on the biological activity. In this study, the phenyl ring was used as the aromatic group instead of the alkyl group in the fourth position of the triazole. Synthesized compounds were derivatized on the phenyl ring with various substituents. Looking at the chemical structure of the compounds (**6b**, **6i**, and **6j**) that showed stronger anticandidal activity, they bear chloro and methoxy substituents at the C-4 position of phenyl. Hence, it can be declared that C-4 of phenyl is a very important position in terms of anticandidal activity. Chloro and methoxy substituents at this position significantly enhance the biological activity.

### 2.3 Cytotoxicity Assay

**Figure 4:**
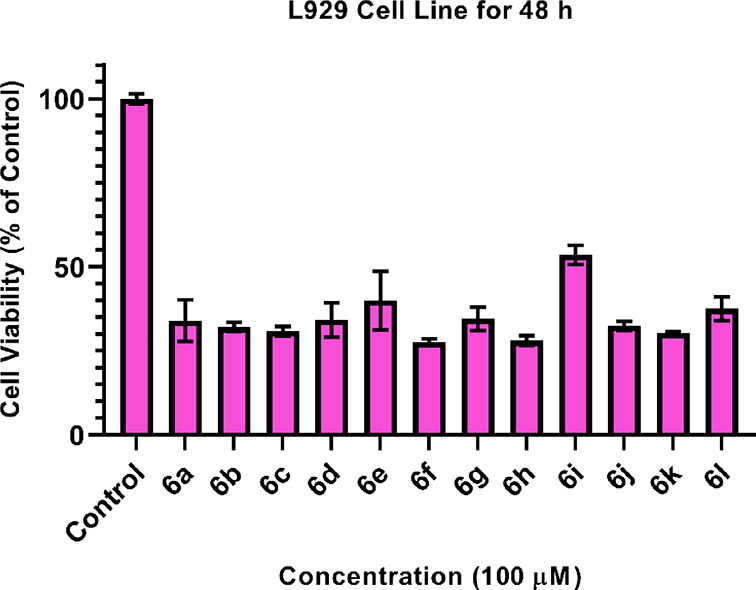
Cell viability of all compounds at 100 μM against L929 cell lines.

The cytotoxic effect of compounds **6a**–**6l** was evaluated against L929 cell lines. For preliminary screening, the cytotoxic bioactivity of synthesized compounds was evaluated in vitro against L929 cell lines with the MTT assay. To evaluate the cytotoxic potency of target compounds, the fibroblast cells were treated with the compounds at a 100 μM constant concentration. Cell viability percentages were calculated after the treatment of cells for 48 h (Figure 4). Preliminary cytotoxic effect results of compounds **6a**–**6l** against L929 fibroblasts are presented in Table 3. As a result of the maximum dose applied, all compounds except compound **6i** showed under 50% viability. However, compound **6i** showed an IC_50_ value above 100 μM, but the cell viability decreased to 53.6% at the maximum dose. As a result of the calculations, it was determined that all the structures except compound **6i** had IC_50_ values below 100 μM and were found to be toxic.

**Table 3:** Cell Viability Percentage of All Compounds at 100 μM against L929 Cell Lines

cell viability (%)	
control	100 ± 1.48
**6a**	34 ± 6.19
**6b**	32.1 ± 1.38
**6c**	30.9 ± 1.42
**6d**	34.2 ± 5.13
**6e**	40 ± 8.71
**6f**	27.6 ± 1.02
**6g**	34.6 ± 3.51
**6h**	28.1 ± 1.48
**6i**	53.6 ± 2.82
**6j**	32.5 ± 1.34
**6k**	30.3 ± 0.41
**6l**	37.6 ± 3.55

### 2.4 Scanning Electron Microscopy with Energy-Dispersive X-ray Analysis (SEM–EDX)

**Figure 5:**
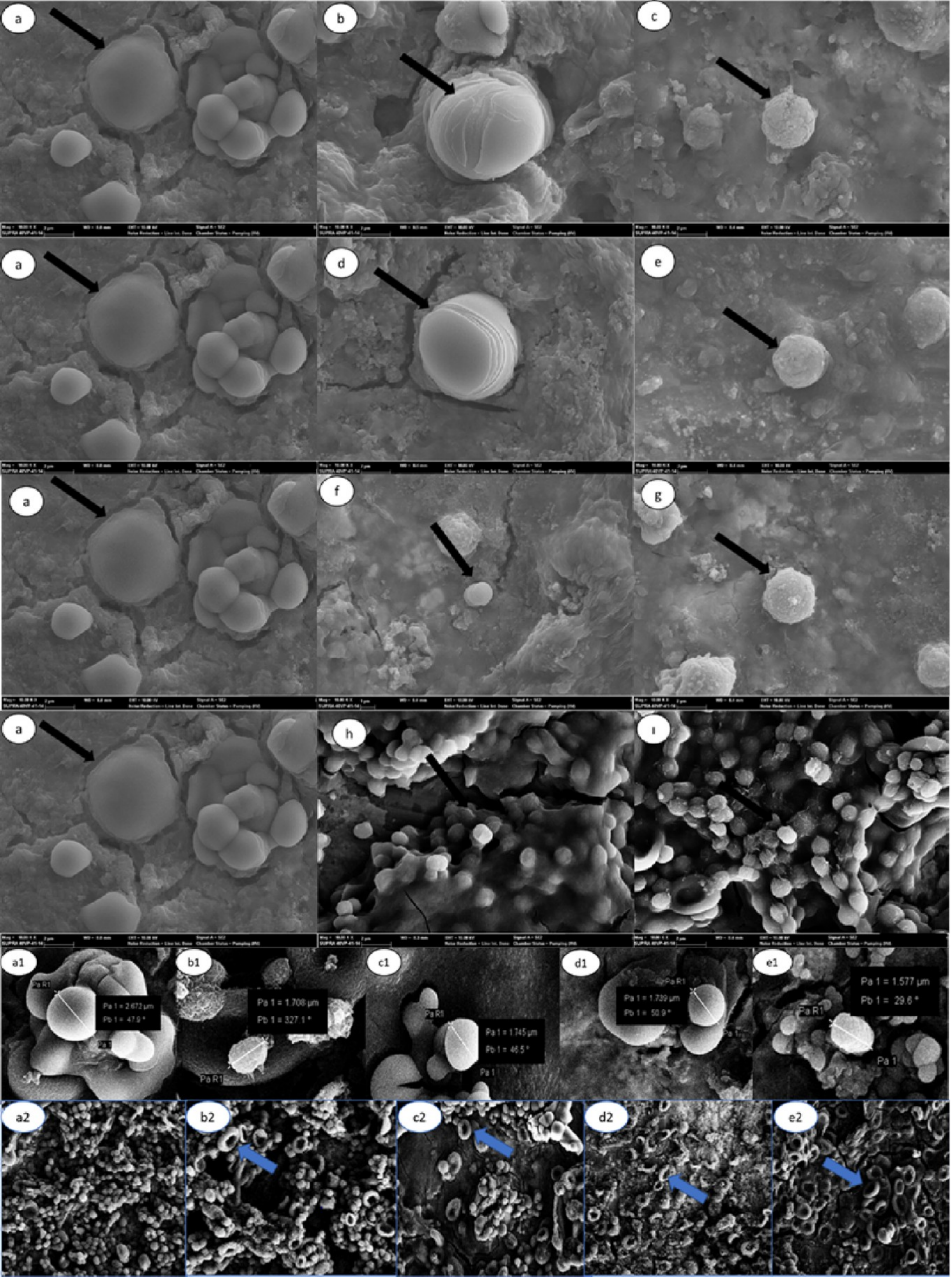
SEM images of untreated and compound-treated *C. glabrata*: (a) *C. glabrata*, (b) 1.95 μg/mL compound **6b**-treated *C. glabrata*, (c) 3.90 μg/mL compound **6b**-treated *C. glabrata*, (d) 1.95 μg/mL compound **6i**-treated *C. glabrata*, (e) 3.90 μg/mL compound **6i**-treated *C. glabrata*, (f) 1.95 μg/mL compound **6j**-treated *C. glabrata*, (g) 3.90 μg/mL compound **6j**-treated *C. glabrata*, (h) 1.95 μg/mL voricanazole-treated *C. glabrata*, (i) 3.90 μg/mL voricanazole treated *C. glabrata*, (a1) *C. glabrata*, (b1) 0.97 μg/mL (MIC) compound **6b**-treated *C. glabrata*, (c1) 0.97 μg/mL (MIC) compound **6i**-treated *C. glabrata*, (d1) 0.97 μg/mL (MIC) compound **6j**-treated *C. glabrata*, (e1) 0.97 μg/mL voricanazole-treated *C. glabrata* (10 kv, magnification: ×10000), (a2) *C. glabrata*, (b2) 0.97 μg/mL (MIC) compound **6b**-treated *C. glabrata*, (c2) 0.97 μg/mL (MIC) compound **6i**-treated *C. glabrata*, (d2) 0.97 μg/mL (MIC) compound **6j**-treated *C. glabrata*, (e2) 0.97 μg/mL voricanazole-treated *C. glabrata* (10 kv, magnification: ×1000).

SEM analyses were performed to examine the effects of **6a**, **6i**, and **6j** compounds on *C. glabrata* cells under in vivo experimental conditions. The SEM images are given in Figure 5. When Figure 5 is examined, it is observed that the cell surface has a regular, homogeneous, and smooth structure in the SEM image of the *C. glabrata* cell that has not been treated with the compound (Figure 5a). Also, in Figure 5a, it is noticeable that healthy cells cluster as a result of budding during natural development. SEM images of *C. glabrata* exposed to test compounds (compounds **6b**, **6i**, and **6j**) show that the cells are cracked, wrinkled, and shrunken (Figure 5b–g). According to the SEM images, when the cell diameters of the untreated cells (Figure 5a1) and cells treated with the MIC concentration (0.97 μg/mL) of test compounds were compared, there was a decrease in the cell diameters (Figure 5b1–d1). A similar situation occurs after the treatment of the positive standard, voriconazole (Figure 5e1). In addition, abnormal folds and indentations in the cell wall and loss of membrane integrity were observed from time to time as cellular deformation increased as the compound concentration increased (Figure 5c,e,g). In addition, spherical or irregularly shaped cellular remnants caused by degenerated cells were often found on the surfaces of cells exposed to the compound at a concentration of 3.90 μg/mL (Figure 5c,e,g). The same situation is observed in cells exposed when voriconazole is used (Figure 5h,i). As a result, it is clearly understood that compounds **6b**, **6i**, and **6j**, which are the tested compounds in SEM analyses, damage the cell surface of *C. glabrata* and cause shrinkage and leakage of intracellular materials and these effects depend on the concentration of the compounds (Figure 5a–g,a1–d1). When the results of the antifungal effects of different substances in the literature are examined against several *Candida* genera, their antifungal properties show via some morphological changes such as shrinking, shriveling, and cracking. These changes cause damage to the cell surface integrity, and residues because of intracellular substance leakage are observed.^30−34^ However, the most important concern here is whether the deformation of the cell surface is caused by ergosterol, a typical plasma component located under the cell wall in the outermost part of the cell. Previously, it has been shown in the literature that cell-wall mannoproteins and related molecules bind and transport extracellular sterols in *C. albicans*.^35^ In fact, ergosterol is the main lipid component of fungal extracellular vesicles, which are the main vehicles of trans-cell wall transport in fungi.^36^ Another study in the literature reported that the passage of ergosterol-containing vesicles through the cell wall means that this sterol is a temporary cell-wall component.^37^ It is known that azole-group antifungal drugs/compounds, such as voriconazole and fluconazole,^33−38^ show their mechanism of action by inhibiting the synthesis of ergosterol via inhibiting lanosterol 14α-demethylase (LDM).^39−41^ Previously, Madhavan et al.^42^ showed that fluconazole and voriconazole changed the shape of different *Candida* cells such as *Candida glabrata* (*C. glabrata*), *Candida parapsilosis* (*C. parapsilosis*), and *Candida rugosa* (*C. rugosa*), and especially, these compounds caused dimples on the cell surface as seen using SEM analyses. Recently, Suchodolski et al.^43^ investigated whether these cell surface dimples in the SEM images of *Candida albicans* (*C*. *albicans*) cells treated with fluconazole are caused by changes in the amount of ergosterol in the plasma membrane. Suchodolski et al. reported^43^ that *C. albicans* cells with normal levels of ergosterol were ovular cells with smooth surfaces but both *C. albicans* erg11Δ/Δ mutant cells (they cannot produce ergosterol) and *C. albicans* cells treated with fluconazole (1 μg/mL) had similar dimple displays on their surface in the SEM images. In addition to SEM analysis, they showed the presence of white-stained parts expressing the presence of chitin in *C. albicans* erg11Δ/Δ mutants via the chitin-stained calcofluor white method using confocal microscope images and concluded that the dimples formed on the cell surface are associated with depletion of ergosterol. In the SEM images of this study, similar dimples were detected in *C. glabrata* cells exposed to 0.97 μg/mL of voriconazole (Figure 5e2). Similar to the results of Suchodolski et al.,^43^*C. glabrata* cells, which were not treated with any compound, appear to be round or oval-shaped cells in clusters (Figure 5a2) in our study. *C. glabrata* cells exposed to test compounds **6a**, **6i**, and **6j** showed similar dimples to those in voriconazole (Figure 5b2–d2). The results obtained from the SEM analyses in this study were found to be compatible with the SEM results of similar studies in the literature. Our results of the SEM analysis indicated that the newly synthesized test compounds acted by inhibiting ergosterol biosynthesis in the same way as azole drugs. Furthermore, this hypothesis was supported by molecular mechanics studies; thus, in vitro and in silico results are in a harmony.

### 2.5 Molecular Docking Analysis

**Figure 6:**
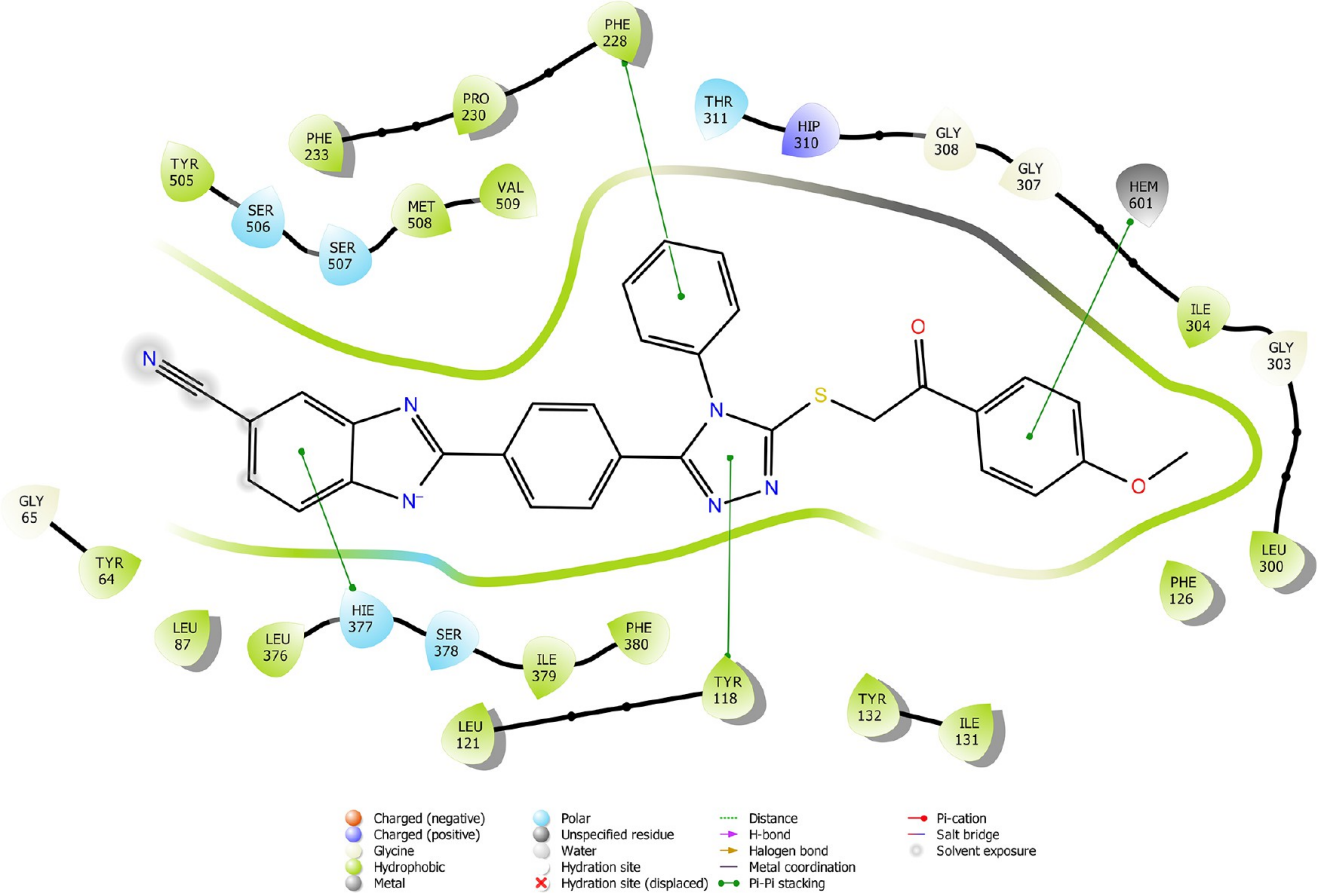
2D schematic protein–ligand interactions of compound **6b** in the active site of lanosterol 14α-demethylase (PDB ID: 5TZ1).

According to the molecular docking study (Figures 6^7^,8,9,10,11,12,13,14,15), compounds **6b**, **6i**, and **6j** were fit into the LDM enzyme active pocket. In a previous study, the Tyr118 amino acid and HEM601 protein were described as essential residues, and in this study, the synthesized active compounds interacted significantly with Tyr118, His377, and HEM601 residues. When the interactions with those amino acids were observed as π–π stacking, the interactions with HEM were seen as π–π stacking and π–cation interactions. Because of that, we envisaged that the antifungal effects of compounds **6b**, **6i**, and **6j** are caused by the breaking cell integrity due to inhibition of the LDM enzyme. We thought that compound **6i** has probably higher inhibitory activity due to H-bonding with Tyr132 than the other two compounds. In addition, compound **6b** (Tyr64, Gly303, Ser378, and Met508), **6i** (Phe126, Ser378, Tyr505, and Ser507), and **6j** (Tyr64, Tyr132, and Met508) formed four, four, and three aromatic bonds, respectively. Although these bonds are at least half-strong of conventional hydrogen bonds, they are important since they can stabilize the ligand–enzyme complex. Moreover, the locations of 4-chloro (**6i**) and 2,4-dichlorophenyl (**6j**) derivatives at active pockets were very similar, their *N*-phenyltriazole moieties faced toward the HEM group, benzimidazole moieties were located near the Hie377 amino acid, and the phenacyl moieties were close to Phe126 amino acids. On the other hand, the 4-methoxyphenyl (**6b**) derivative was found to be located a bit differently from chlorine derivatives. Its *N*-phenyltriazole group faced toward Phe228 and Tyr118 amino acids, and this let its phenacyl group interact with the HEM protein and also with the Gly303 amino acid. One of the possible explanations for this variety is most probably related to volume differences of chlorine and methoxy moieties, and the other one is due to the lipophilic character of the chlorine atom. This location of the enzyme was formed by slightly more hydrophilic amino acids, such as Thr122, Gln142, Lys143, and Gly303. As a result, the methoxy phenyl group may be preferred to interact with these amino acids. As a result, the in silico study explained the possible action mechanism and also structure–activity relationship.

**Figure 7:**
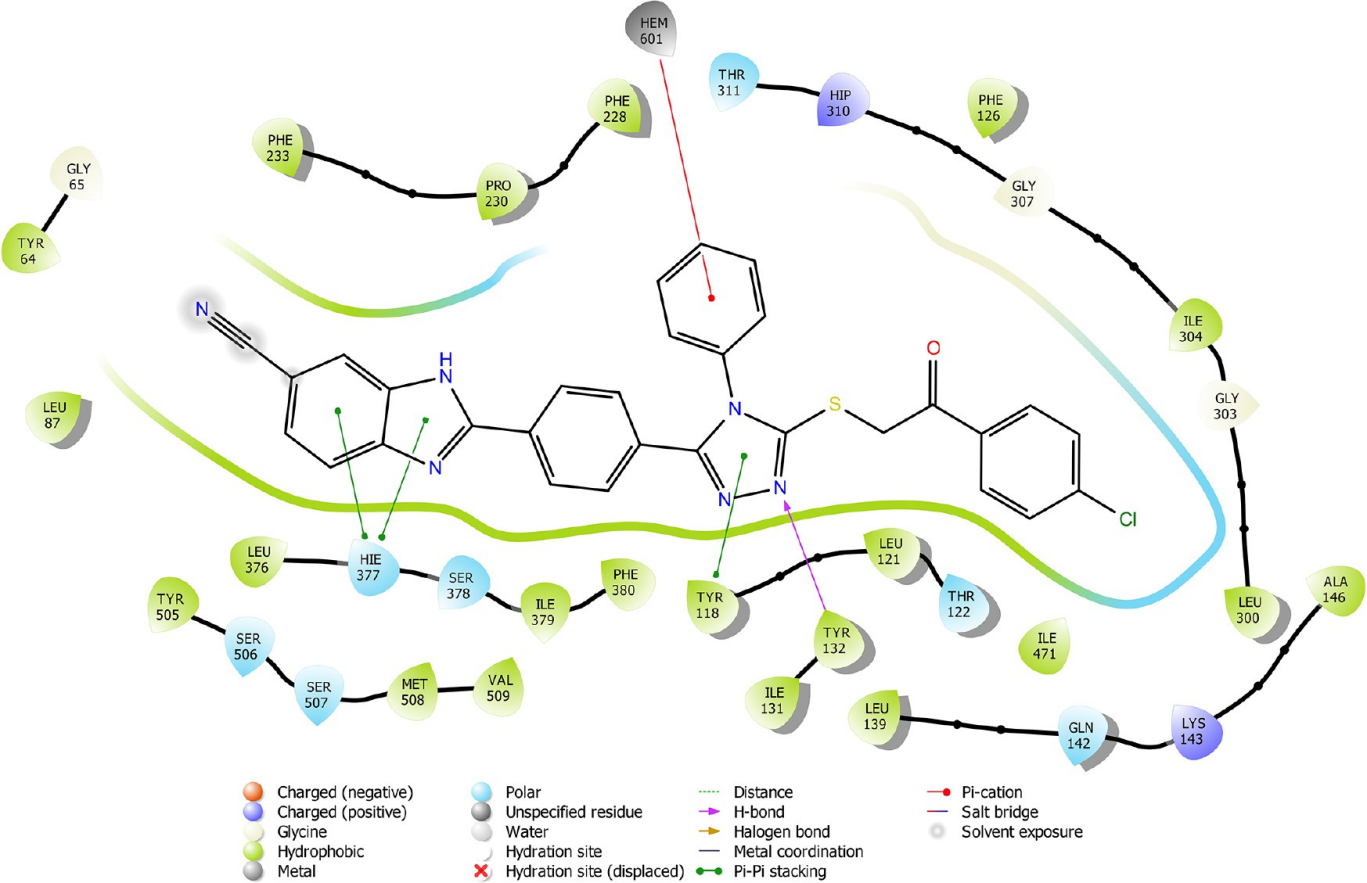
2D schematic protein–ligand interactions of compound **6i** in the active site of lanosterol 14α-demethylase (PDB ID: 5TZ1).

**Figure 8:**
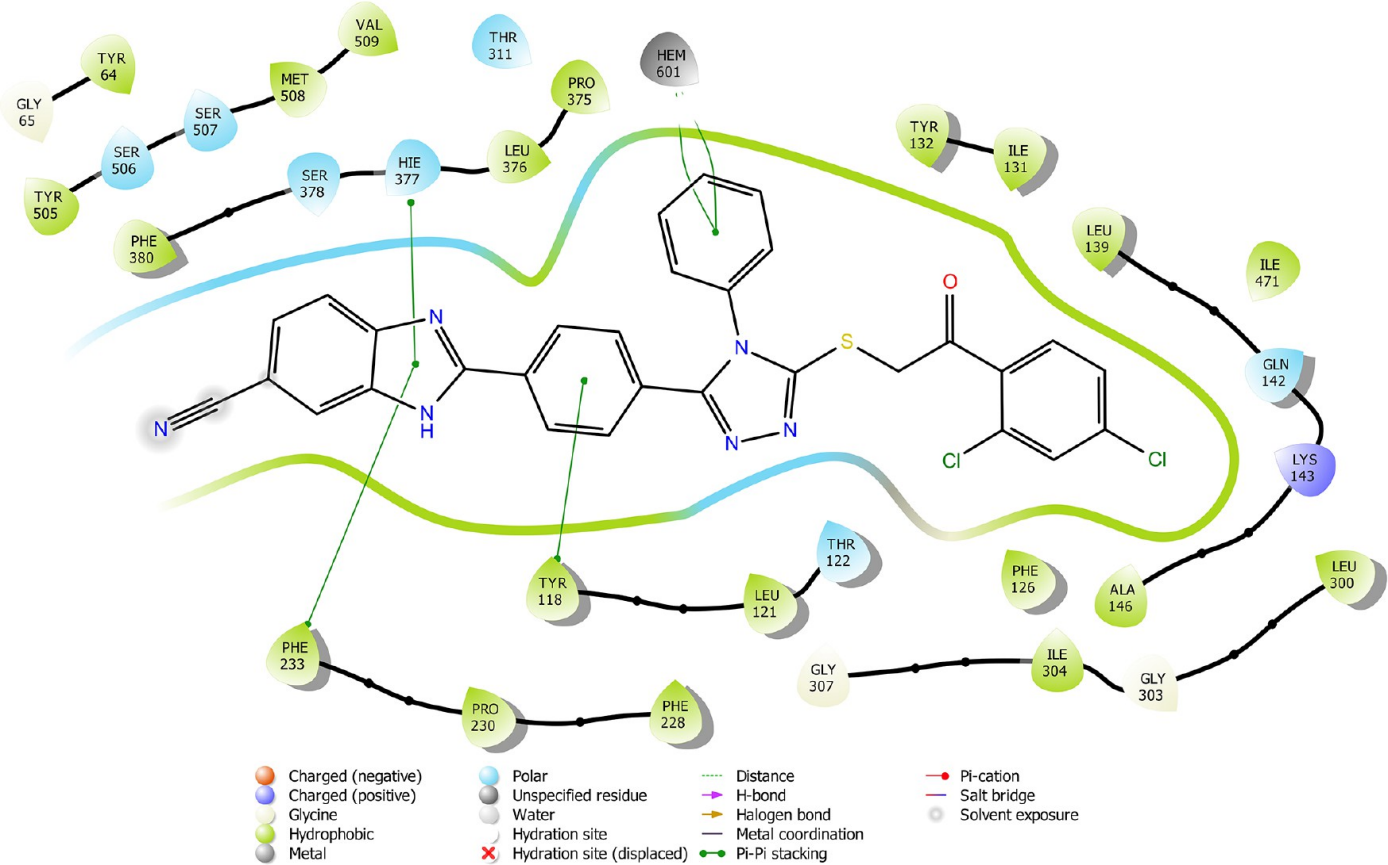
2D schematic protein–ligand interactions of compound **6j** in the active site of lanosterol 14α-demethylase (PDB ID: 5TZ1).

**Figure 9:**
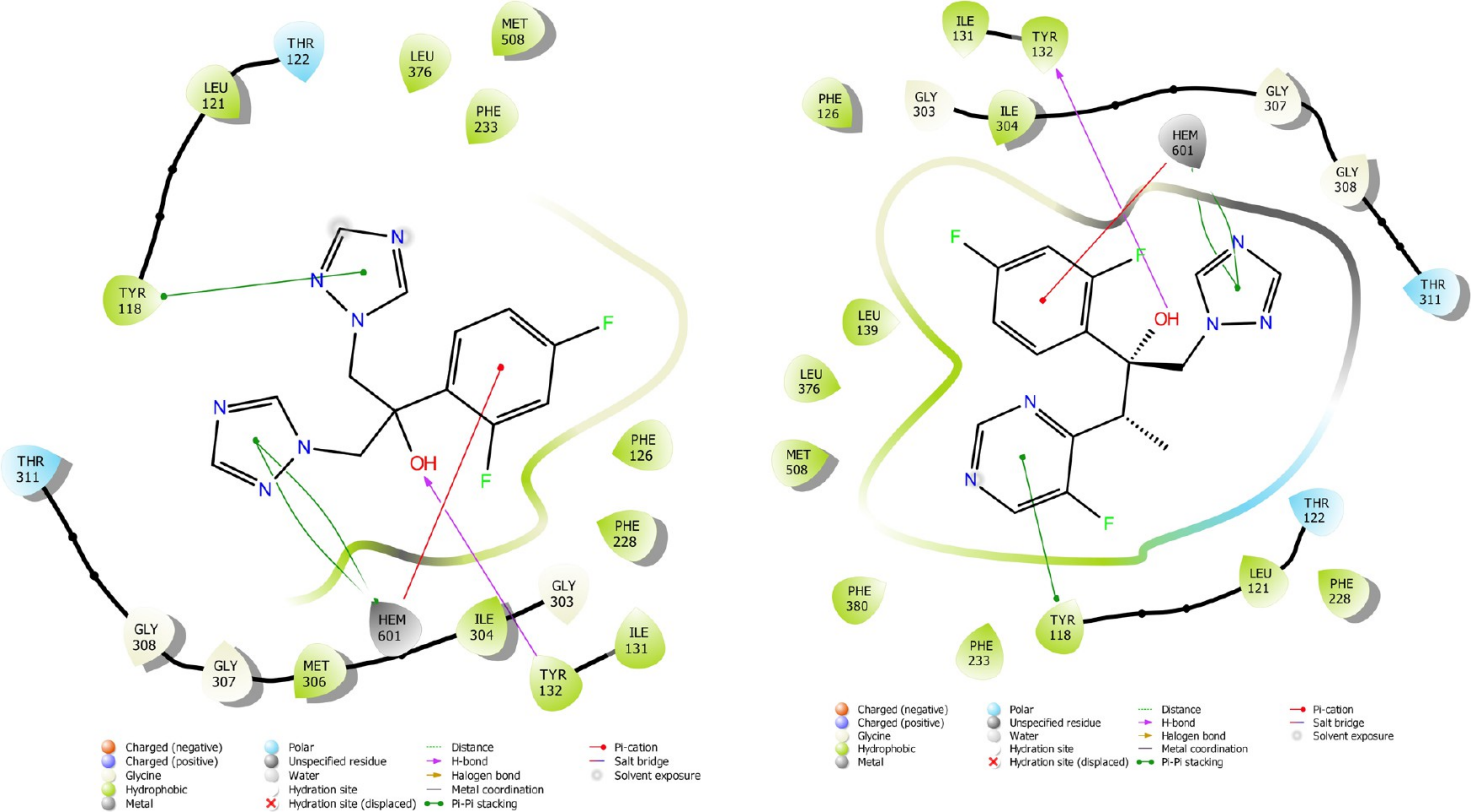
2D schematic protein–ligand interactions of fluconazole and voriconazole in the active site of lanosterol 14α-demethylase (PDB ID: 5TZ1).

**Figure 10:**
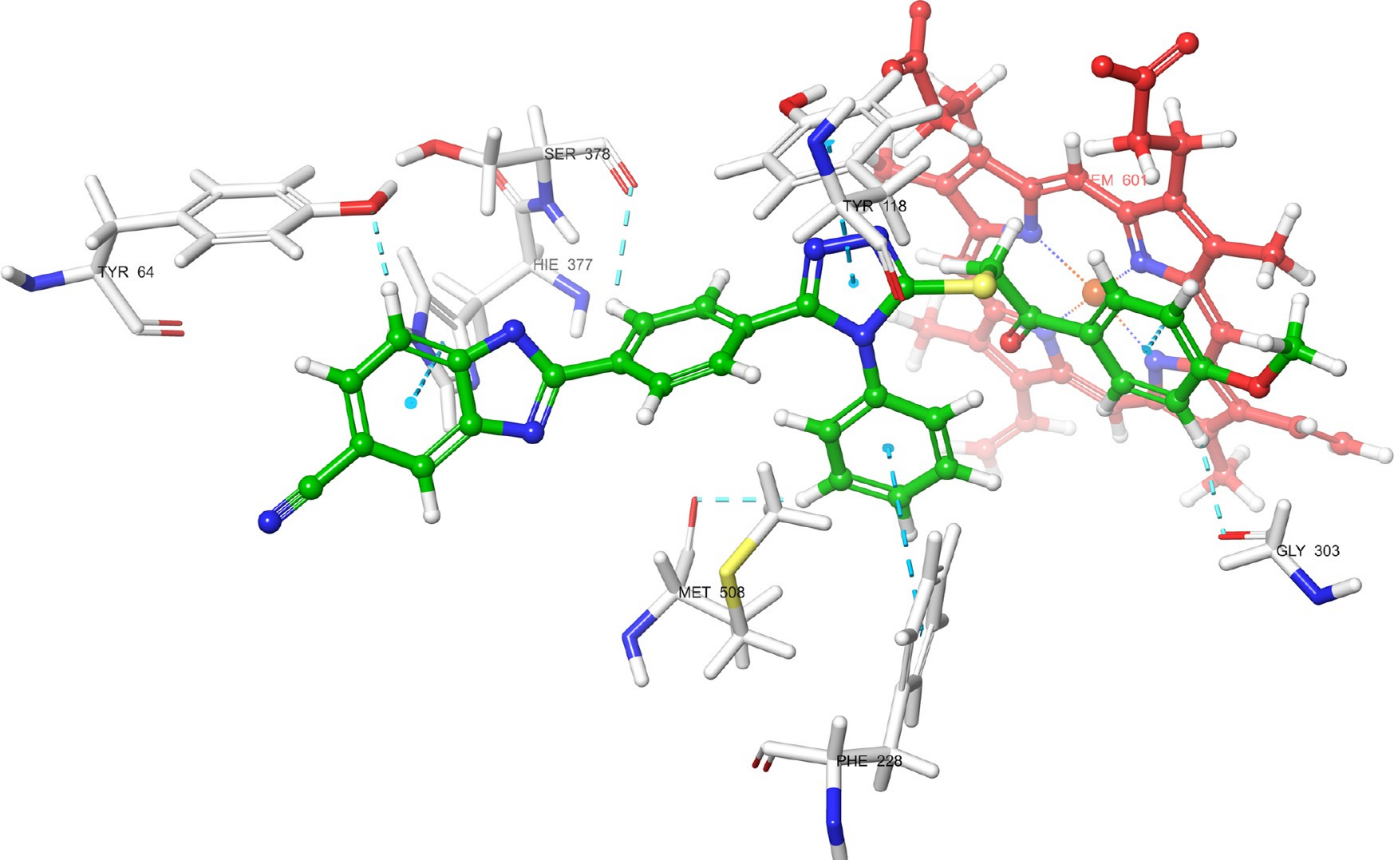
3D schematic protein–ligand interactions of compound **6b** in the active site of lanosterol 14α-demethylase (PDB ID: 5TZ1).

**Figure 11:**
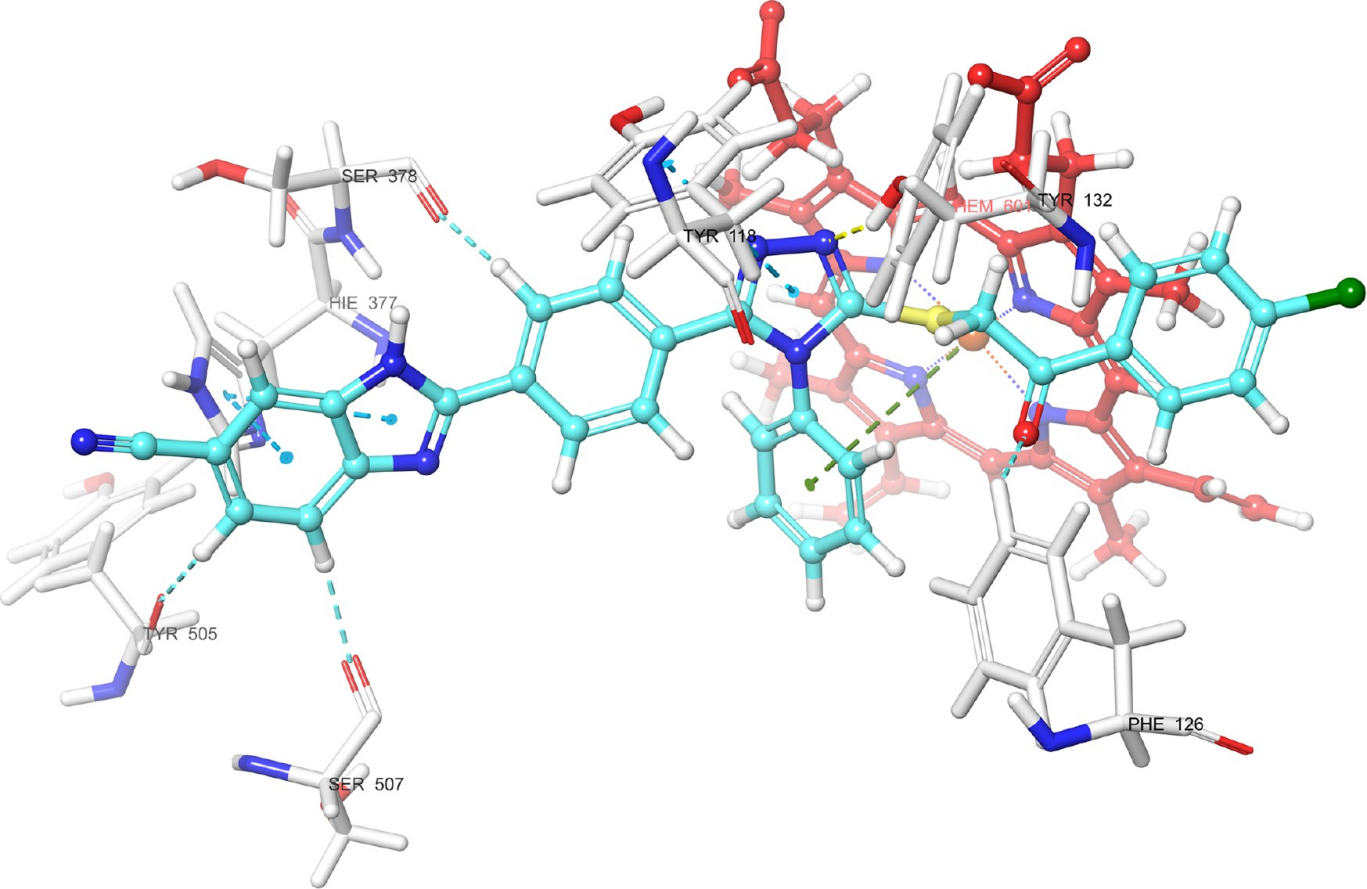
3D schematic protein–ligand interactions of compound **6i** in the active site of lanosterol 14α-demethylase (PDB ID: 5TZ1).

**Figure 12:**
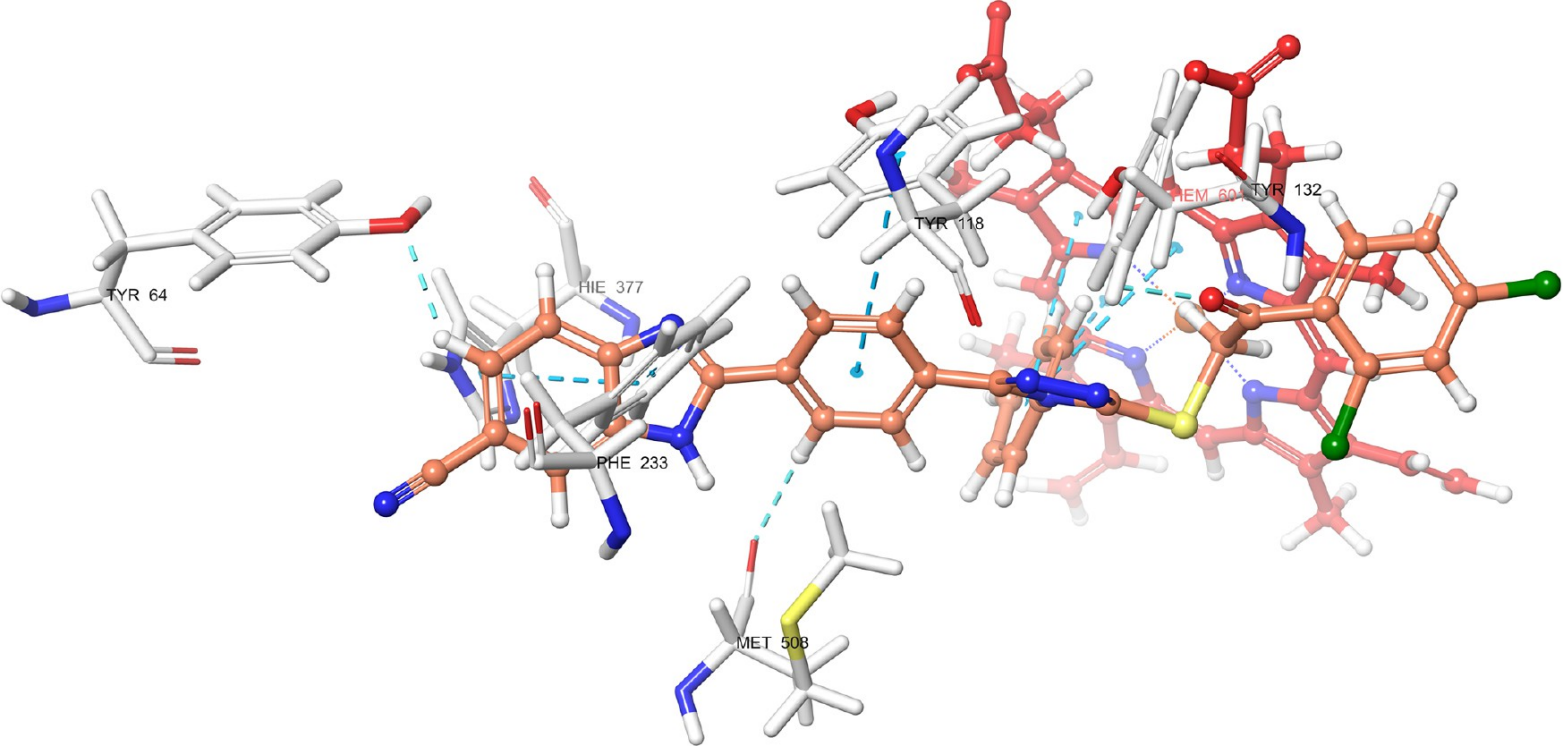
3D schematic protein–ligand interactions of compound **6j** in the active site of lanosterol 14α-demethylase (PDB ID: 5TZ1).

**Figure 13:**
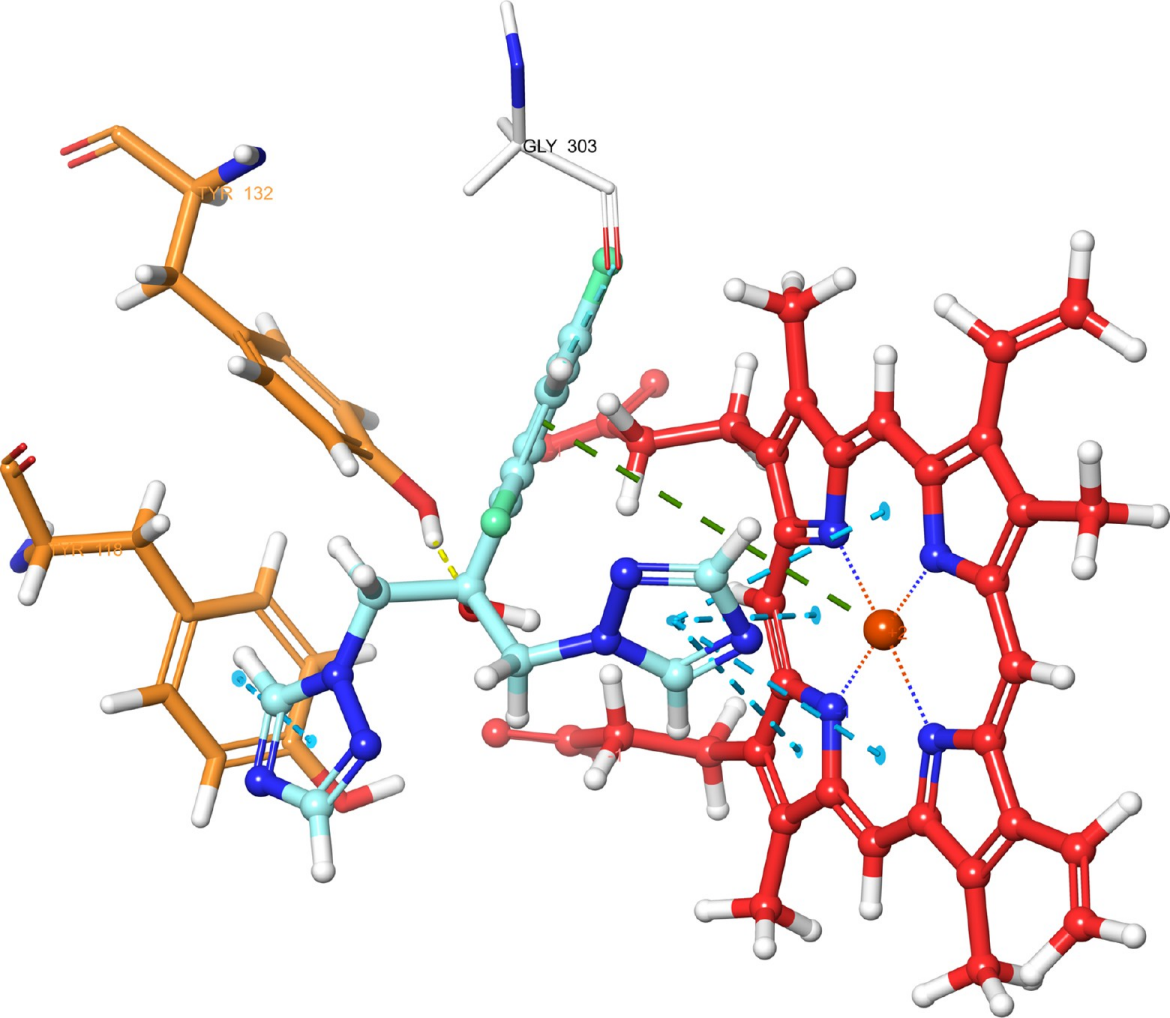
3D schematic protein–ligand interactions of fluconazole in the active site of lanosterol 14α-demethylase (PDB ID: 5TZ1).

**Figure 14:**
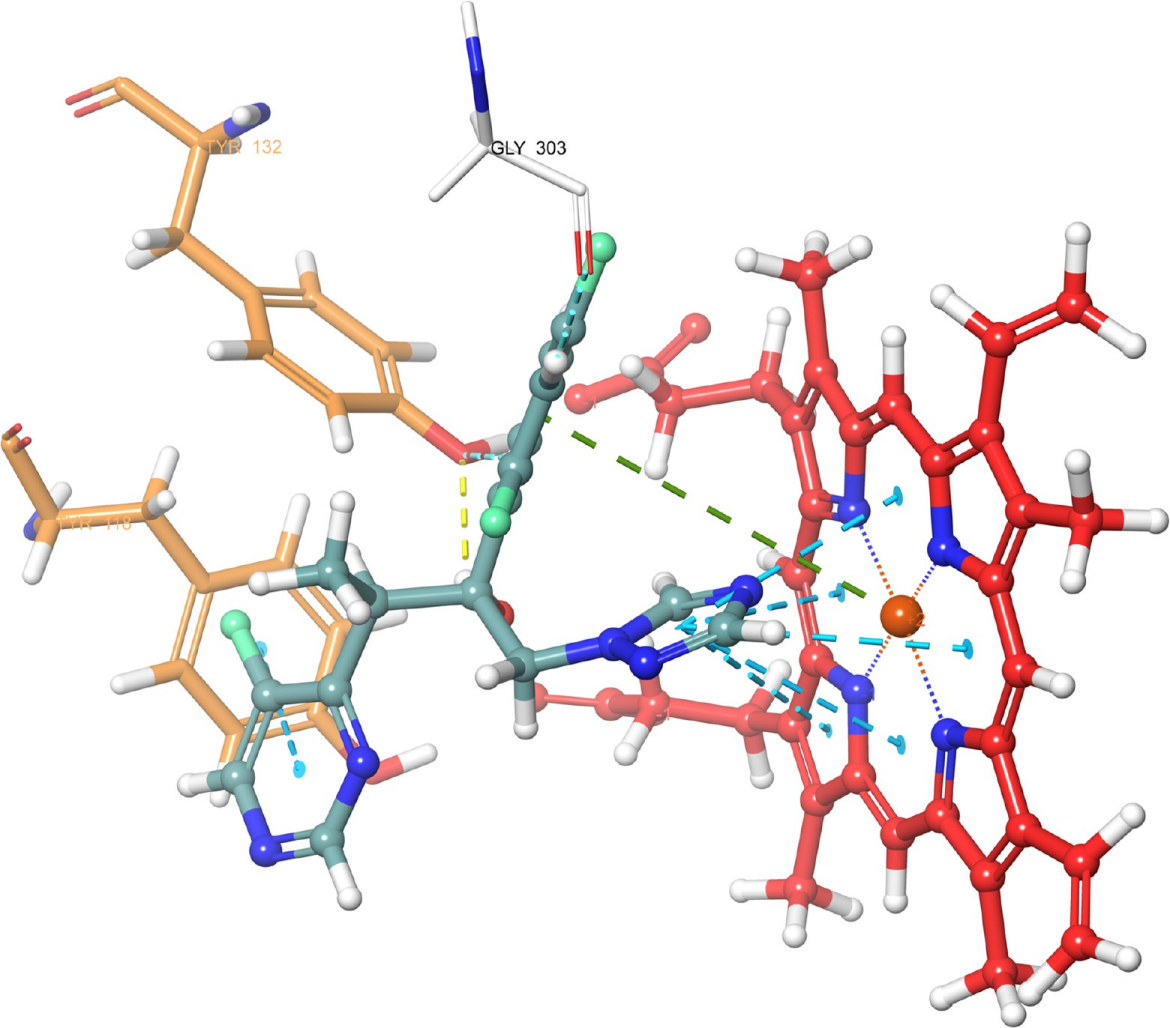
3D schematic protein–ligand interactions of voriconazole in the active site of lanosterol 14α-demethylase (PDB ID: 5TZ1).

**Figure 15:**
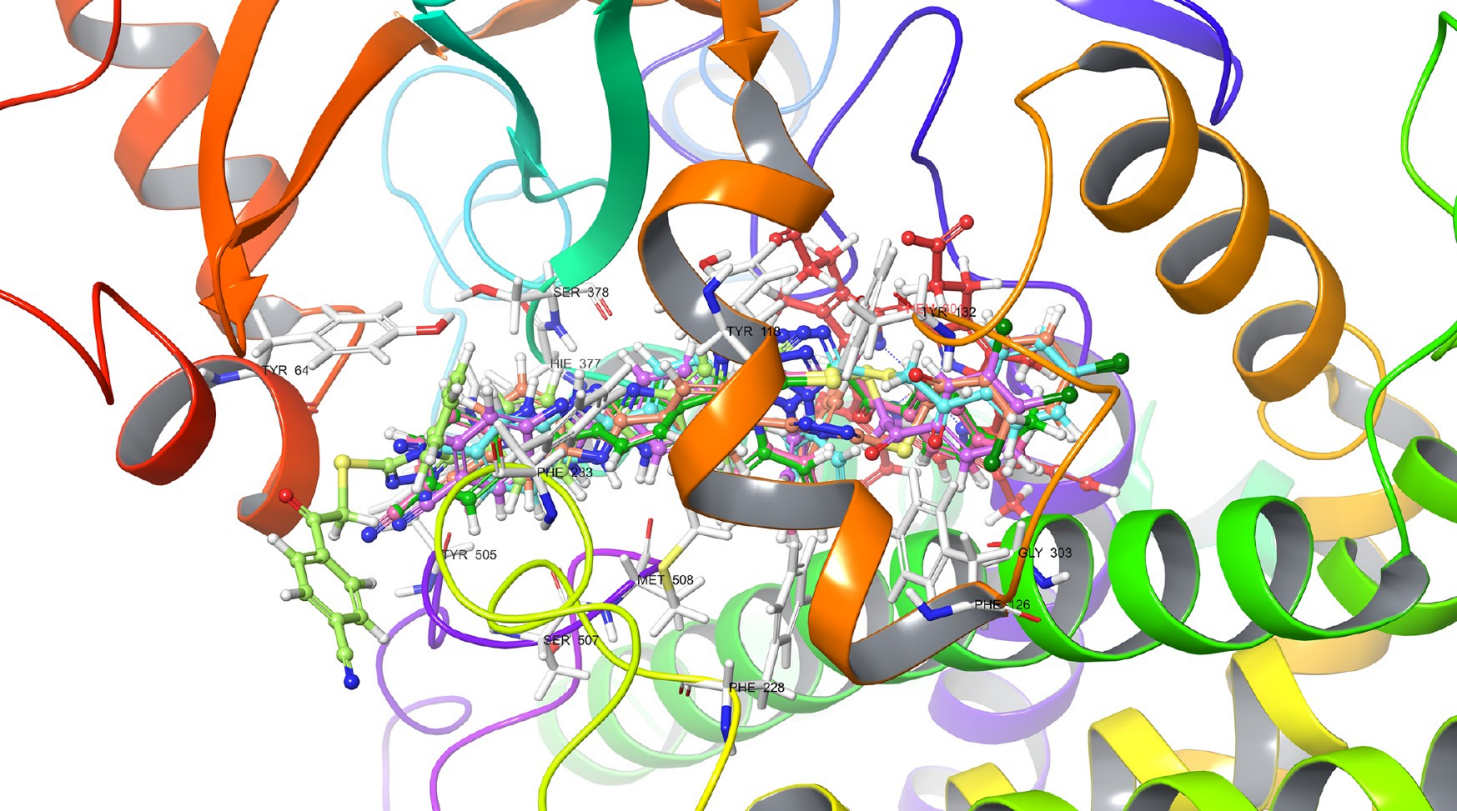
Superimpose of all compounds in the cavity entrance of lanosterol 14α-demethylase (PDB ID: 5TZ1).

## 3 Conclusions

A new series of benzimidazole-triazole derivatives (**6a**–**6l**) was synthesized and characterized with different spectroscopic methods. The target compounds (**6a**–**6l**) were evaluated for antifungal activities against four *Candida* species. All compounds were found to exhibit excellent activity against *C. glabrata*. Especially, compounds **6b**, **6i**, and **6j** were found to be the most effective compounds in the series with an MIC value of 0.97 μg/mL. In addition, molecular docking studies suggested good binding affinity of compounds **6b**, **6j**, and **6i** to the HEME group present in 14α-demethylase (CYP51), which might explain the high antifungal activity found in these compounds. To establish the antifungal selectivity and safety, the cytotoxic effects of the compounds against the L929 healthy cell line were evaluated. SEM analyses were performed to examine the effects of compounds **6a**, **6i**, and 6j on *C. glabrata* cells under in vivo experimental conditions.

## 4 Experimental Section

All the chemicals employed in the synthetic procedure were purchased from Sigma-Aldrich Chemicals (Sigma-Aldrich Corp., St. Louis, MO, USA) or Merck Chemicals (Merck KGaA, Darmstadt, Germany). Melting points of the obtained compounds were determined by an MP90 digital melting point apparatus (Mettler Toledo, OH, USA) and were uncorrected. ^1^H NMR and ^13^C NMR spectra of the synthesized compounds were registered by a Bruker 500 MHz and 125 MHz digital FT-NMR spectrometer (Bruker Bioscience, Billerica, MA, USA) in DMSO-*d*_6_, respectively. Splitting patterns were designated as follows: s: singlet; d: doublet; t: triplet; m: multiplet in the NMR spectra. Coupling constants (*J*) were reported in hertz. M + 1 peaks were determined by a Shimadzu LC/MS ITTOF system (Shimadzu, Tokyo, Japan). All reactions were monitored by thin-layer chromatography (TLC) using Silica Gel 60 F254 TLC plates (Merck KGaA, Darmstadt, Germany).

### 4.1 Chemistry

#### 4.1.1 General Procedure for the Synthesis of Sodium Metabisulfite Salt of Benzaldehyde Derivative (**1**)

Methyl 4-formyl benzoate (5 g, 0.03 mol) was dissolved in ethanol. Sodium metabisulfite (6.84 g, 0.036 mol) solution in ethanol was added dropwise into the benzaldehyde solution. After the dripping was completed, the reaction contents were stirred at room temperature for 1 h. The precipitated product was filtered off.

#### 4.1.2 Synthesis of 4-(5-Cyano-1*H*-benz[*d*]imidazol-2-yl)benzoic Acid Methyl Ester (**2**)

5-Cyano-1,2-phenylenediamine (0.022 mol) was dissolved in DMF, and sodium metabisulfite salt of the benzaldehyde derivative (7.09 g, 0.026 mol) was added. At the end of the reaction, the product was precipitated by pouring the reaction contents into ice water. The precipitated product was filtered off and crystallized from ethanol.

#### 4.1.3 Synthesis of 4-(5-Cyano-1*H*-benz[*d*]imidazol-2-yl)benzohydrazide (**3**)

Compound **2** (0.018 mol) and excess of hydrazine hydrate (5 mL) were placed in the same vial, and ethanol (15 mL) was added. The mixture was refluxed for 12 h. When the reaction was completed, the mixture was poured into iced water, and the product was filtered.

#### 4.1.4 Synthesis of 2-(4-(5-Cyano-1*H*-benz[*d*]imidazol-2-yl)benzoyl)-*N*-phenylhydrazine-1-carbothioamide (**4**)

4-(5-Cyano-1*H*-benz[*d*]imidazol-2-yl)benzohydrazide and phenyl isothiocyanate were dissolved in ethanol. The reaction mixture was boiled under reflux for 4–5 h. At the end of the reaction, the precipitated product was filtered off.

#### 4.1.5 Synthesis of 2-(4-(5-Mercapto-4-phenyl-4*H*-1,2,4-triazol-3-yl)phenyl)-1*H*-benz[*d*]imidazole-5-carbonitrile (**5**)

2-(4-(5-Cyano-1*H*-benz[*d*]imidazol-2-yl)benzoyl)-*N*-phenylhydrazine-1-carbothioamide (**4**) (0.001 mol) in ethanol was refluxed under stirring for 2 h in the presence of NaOH (0.012 mol). After completion of the reaction, the solution was acidified with HCl (37%), and the precipitate was filtered, washed with water, dried, and then recrystallized from ethanol.

#### 4.1.6 Synthesis of Target Compounds (**6a**–**6l**)

A solution of compound **5** (0.001 mol) in acetone (10 mL), an appropriate substituted 2-bromoacetophenone derivative (0.001 mol), and potassium carbonate (0.138 g, 0.001 mol) were refluxed at 40 °C for 12 h. The solvent was evaporated, and the residue was washed with water, dried, and recrystallized from ethanol.

##### 4.1.6.1 2-(4-(5-((2-(4-Nitrophenyl)-2-oxoethyl)thio)-4-phenyl-4*H*-1,2,4-triazol-3-yl)phenyl)-1*H*-benz[*d*]imidazole-5(6)-carbonitrile (**6a**)

Yield: 74%. M.p. 194.6 °C. ^1^H NMR (500 MHz, DMSO-*d*_6_): δ 4.97 (2H, s, CH_2_), 7.15 (3H, d, *J* = 8.67 Hz, aromatic CH), 7.37 (3H, d, *J* = 7.98 Hz, aromatic CH), 7.45 (1H, d, *J* = 8.43 Hz, aromatic CH), 7.74 (1H, d, *J* = 8.34 Hz, aromatic CH), 7.80 (2H, s, aromatic CH), 7.95 (3H, d, *J* = 7.98 Hz, aromatic CH), 8.17 (3H, d, *J* = 8.67 Hz, aromatic CH). ^13^C NMR (125 MHz, DMSO-*d*_6_): δ 30.05, 103.87, 106.99, 109.60, 112.74, 120.42, 123.92, 124.26, 124.47, 125.81, 127.49, 128.08, 128.64, 128.97, 129.64, 129.70, 130.04, 130.42, 130.98, 146.82, 149.05, 154.73, 161.02, 182.74. [M + H]^+^/2 calcd for C_30_H_19_N_7_O_3_S: 279.5708; found: 279.5702. Anal. calcd for C_30_H_19_N_7_O_3_S, C, 64.62; H, 3.43; N, 17.58. Found: C, 64.74; H, 3.44; N, 17.61.

##### 4.1.6.2 2-(4-(5-((2-(4-Methoxyphenyl)-2-oxoethyl)thio)-4-phenyl-4*H*-1,2,4-triazol-3-yl)phenyl)-1*H*-benz[*d*]imidazole-5(6)-carbonitrile (**6b**)

Yield: 78%. M.p. 177.6 °C. ^1^H NMR (500 MHz, DMSO-*d*_6_): δ = 3.84 (3H, s, OCH_3_), 4.99 (2H, s, -CH_2_), 7.11 (3H, d, *J* = 8.46 Hz, aromatic CH), 7.37–7.42 (4H, m, aromatic CH), 7.71 (2H, d, *J* = 8.28 Hz, aromatic CH), 7.79 (1H, s, aromatic CH), 8.12–8.22 (6H, m, aromatic CH). ^13^C NMR (125 MHz, DMSO-*d*_6_): δ (ppm) 29.70, 56.16, 113.74, 114.55, 114.78, 116.04, 116.74, 118.57, 120.27, 120.90, 121.95, 123.61, 126.56, 127.54, 128.32, 129.09, 129.86, 130.86, 132.39, 134.39, 140.07, 143.29, 147.51, 151.50, 185.27. [M + H]^+^/2 calcd for C_31_H_22_N_6_O_2_S: 272.0835; found: 272.0825. Anal. calcd for C_31_H_22_N_6_O_2_S, C, 68.62; H, 4.09; N, 15.49. Found: C, 68.83; H, 4.07; N, 15.53.

##### 4.1.6.3 2-(4-(5-((2-(4-Cyanophenyl)-2-oxoethyl)thio)-4-phenyl-4*H*-1,2,4-triazol-3-yl)phenyl)-1*H*-benz[*d*]imidazole-5(6)-carbonitrile (**6c**)

Yield: 71%. M.p. 237.8 °C. ^1^H NMR (500 MHz, DMSO-*d*_6_): δ = 4.97 (2H, s, CH_2_), 7.11–7.14 (3H, m, aromatic CH), 7.40–7.42 (1H, m, aromatic CH), 7.74–7.83 (6H, m, aromatic CH), 7.97–8.00 (3H, m, aromatic CH), 8.17 (3H, d, *J* = 7.50 Hz, aromatic CH). ^13^C NMR (125 MHz, DMSO-*d*_6_): δ (ppm) 31.14, 103.20, 104.60, 111.42, 118.72, 120.44, 121.31, 122.96, 126.09, 128.10, 128.64, 128.91, 129.24, 129.63, 130.37, 132.67, 133.03, 133.35, 135.60, 135.72, 135.99, 140.22, 160.71, 186.19. [M + H]^+^/2 calcd for C_31_H_19_N_7_OS: 269.5759; found: 269.5748. Anal. calcd for C_31_H_19_N_7_OS, C, 69.26; H, 3.56; N, 18.24. Found: C, 69.37; H, 3.57; N, 18.30.

##### 4.1.6.4 2-(4-(5-((2-(4-Fluorophenyl)-2-oxoethyl)thio)-4-phenyl-4*H*-1,2,4-triazol-3-yl)phenyl)-1*H*-benz[*d*]imidazole-5(6)-carbonitrile (**6d**)

Yield: 69%. M.p. 277.4 °C. ^1^H NMR (500 MHz, DMSO-*d*_6_): δ = 4.89 (2H, s, CH_2_), 7.21 (3H, d, *J* = 8.82 Hz, aromatic CH), 7.26–7.32 (2H, m, aromatic CH), 7.46–7.53 (2H, m, aromatic CH), 7.60 (2H, d, *J* = 8.40 Hz, aromatic CH), 7.84 (2H, d, *J* = 8.40 Hz, aromatic CH), 7.91 (1H, s, aromatic CH), 8.01–8.04 (1H, m, aromatic CH), 8.31 (3H, d, *J* = 8.73 Hz, aromatic CH).^13^C NMR (125 MHz, DMSO-*d*_6_): δ (ppm) 30.16, 112.01, 114.38, 115.96, 116.18, 116.38, 120.52, 122.06, 123.60, 126.31, 127.49, 128.98, 129.22, 129.58, 131.90, 132.27, 132.51, 132.61, 135.34, 136.29, 139.04, 145.27, 148.81, 186.01. [M + H]^+^/2 calcd for C_30_H_19_N_6_OFS: 266.0735; found: 266.0724. Anal. calcd for C_30_H_19_N_6_OFS, C, 67.91; H, 3.61; N, 15.84. Found: C, 68.09; H, 3.61; N, 15.89.

##### 4.1.6.5 2-(4-(5-((2-([1,1′-Biphenyl]-4-yl)2-oxoethyl)thio)-4-phenyl-4*H*-1,2,4-triazol-3-yl)phenyl)-1*H*-benz[*d*]imidazole-5(6)-carbonitrile (**6e**)

Yield: 79%. M.p. 224.4 °C. ^1^H NMR (500 MHz, DMSO-*d*_6_): δ = 5.01 (2H, s, CH_2_), 7.13 (3H, d, *J* = 8.88 Hz, aromatic CH), 7.45 (2H, dd, *J*_1_ = 1.44 Hz, *J*_2_ = 8.28 Hz, aromatic CH), 7.55–7.60 (4H, m, aromatic CH), 7.68–7.75 (4H, m, aromatic CH), 7.81 (2H, s, aromatic CH), 8.04–8.07 (3H, m, aromatic CH), 8.16 (3H, d, *J* = 8.79 Hz, aromatic CH). ^13^C NMR (125 MHz, DMSO-d_6_): δ (ppm) 30.93, 117.23, 117.67, 119.77, 120.36, 122.13, 124.05, 124.64, 127.35, 127.41, 127.51, 128.20, 128.86, 129.55, 129.60, 129.75, 130.32, 130.42, 132.50, 134.30, 136.49, 137.55, 138.41, 139.29, 141.12, 144.40, 145.76, 181.26. [M + H]^+^/2 calcd for C_36_H_24_N_6_OS: 295.0939; found: 295.0934. Anal. calcd for C_36_H_24_N_6_OS, C, 73.45; H, 4.11; N, 14.28. Found: C, 73.70; H, 4.10; N, 14.30.

##### 4.1.6.6 2-(4-(5-((2-(4-Bromophenyl)-2-oxoethyl)thio)-4-phenyl-4*H*-1,2,4-triazol-3-yl)phenyl)-1*H*-benz[*d*]imidazole-5(6)-carbonitrile (**6f**)

Yield: 77%. M.p. 279.3 °C. ^1^H NMR (500 MHz, DMSO-*d*_6_): δ = 4.99 (2H, s, CH_2_), 7.18 (3H, d, *J* = 8.52 Hz, aromatic CH), 7.54–7.56 (1H, m, aromatic CH), 7.65 (3H, d, *J* = 8.34 Hz, aromatic CH), 7.79–7.82 (2H, m, aromatic CH), 7.87 (1H, s, aromatic CH), 8.07 (3H, d, *J* = 8.37 Hz, aromatic CH), 8.22–8.25 (3H, m, aromatic CH). ^13^C NMR (125 MHz, DMSO-*d*_6_): δ (ppm) 30.05, 117.56, 120.49, 121.07, 122.11, 123.45, 125.62, 127.52, 128.26, 129.25, 129.57, 129.74, 130.44, 130.86, 131.42, 131.75, 132.04, 132.15, 132.41, 136.48, 137.33, 139.97, 142.17, 182.05. [M + H]^+^/2 calcd for C_30_H_19_N_6_OSBr: 296.0335; found: 296.0323. Anal. calcd for C_30_H_19_N_6_OSBr, C, 60.92; H, 3.24; N, 14.21. Found: C, 61.08; H, 3.23; N, 14.24.

##### 4.1.6.7 2-(4-(5-((2-(3,4-Dichlorophenyl)-2-oxoethyl)thio)-4-phenyl-4*H*-1,2,4-triazol-3-yl)phenyl)-1*H*-benz[*d*]imidazole-5(6)-carbonitrile (**6g**)

Yield: 73%. M.p. >300 °C. ^1^H NMR (500 MHz, DMSO-*d*_6_): δ = 4.97 (2H, s, CH_2_), 7.13 (2H, d, *J* = 8.85 Hz, aromatic CH), 7.44 (1H, dd, *J*_1_ = 1.47 Hz, *J*_2_ = 8.13 Hz, aromatic CH), 7.72–7.85 (6H, m, aromatic CH), 7.92–7.94 (2H, m, aromatic CH), 7.97–8.00 (2H, m, aromatic CH), 8.13 (2H, d, *J* = 8.76 Hz, aromatic CH). ^13^C NMR (125 MHz, DMSO-*d*_6_): δ (ppm) 30.05, 120.37, 121.38, 122.10, 123.64, 127.47, 128.21, 128.95, 129.09, 129.32, 129.47, 129.57, 129.66, 129.80, 129.92, 130.23, 130.50, 130.62, 132.95, 133.14, 138.34, 138.73, 140.42, 144.65, 145.11, 182.62. [M + H]^+^ calcd for C_30_H_18_N_6_OSCl_2_: 581.0713; found: 581.0707. Anal. calcd for C_30_H_18_N_6_O_S_Cl_2_, C, 61.97; H, 3.12; N, 14.45. Found: C, 62.15; H, 3.13; N, 14.50.

##### 4.1.6.8 2-(4-(5-((2-(2,4-Difluorophenyl)-2-oxoethyl)thio)-4-phenyl-4*H*-1,2,4-triazol-3-yl)phenyl)-1*H*-benz[*d*]imidazole-5(6)-carbonitrile (**6h**)

Yield: 76%. M.p. 219.4 °C. ^1^H NMR (500 MHz, DMSO-*d*_6_): δ = 5.02 (2H, s, CH_2_), 7.11 (3H, d, *J* = 8.85 Hz, aromatic CH), 7.40 (1H, dd, *J*_1_ = 1.35 Hz, *J*_2_ = 8.25 Hz, aromatic CH), 7.70 (1H, d, *J* = 8.55 Hz, aromatic CH), 7.78 (1H, s, aromatic CH), 8.07 (3H, d, *J* = 8.43 Hz, aromatic CH), 8.15–8.22 (6H, m, aromatic CH). ^13^C NMR (125 MHz, DMSO-*d*_6_): δ (ppm) 30.24, 105.07, 105.77, 111.55, 111.71, 113.05, 117.20, 120.08, 120.34, 122.19, 122.35, 125.21, 126.56, 127.52, 128.22, 128.75, 129.26, 129.36, 129.61, 133.36, 134.01, 134.82, 139.04, 141.83, 144.46, 180.00. [M + H]^+^/2 calcd for C_30_H_18_N_6_OF_2_S: 275.0688; found: 275.0679. Anal. calcd for C_30_H_18_N_6_OF_2_S, C, 65.68; H, 3.31; N, 15.32. Found: C, 65.74; H, 3.32; N, 15.35.

##### 4.1.6.9 2-(4-(5-((2-(4-Chlorophenyl)-2-oxoethyl)thio)-4-phenyl-4*H*-1,2,4-triazol-3-yl)phenyl)-1*H*-benz[*d*]imidazole-5(6)-carbonitrile (**6i**)

Yield: 76%. M.p. 197.9 °C. ^1^H NMR (500 MHz, DMSO-*d*_6_): δ = 4.87 (2H, s, CH_2_), 6.85 (2H, d, *J* = 8.25 Hz, aromatic CH), 7.12 (3H, d, *J* = 8.91 Hz, aromatic CH), 7.40–7.47 (6H, m, aromatic CH), 7.73 (1H, d, *J* = 8.28 Hz, aromatic CH), 7.80 (1H, s, aromatic CH), 8.15 (3H, d, *J* = 8.85 Hz, aromatic CH). ^13^C NMR (125 MHz, DMSO-*d*_6_): δ (ppm) 29.40, 121.34, 126.84, 127.52, 128.21, 128.59, 128.79, 128.83, 129.20, 129.38, 129.56, 129.72, 130.01, 130.32, 130.44, 130.79, 130.91, 131.61, 131.88, 134.19, 138.86, 141.03, 143.01, 181.36. [M + H]^+^/2 calcd for C_30_H_19_N_6_OSCl: 274.0588; found: 274.0579. Anal. calcd for C_30_H_19_N_6_OSCl, C, 65.87; H, 3.50; N, 15.36. Found: C, 66.06; H, 3.51; N, 15.40.

##### 4.1.6.10 2-(4-(5-((2-(2,4-Dichlorophenyl)-2-oxoethyl)thio)-4-phenyl-4*H*-1,2,4-triazol-3-yl)phenyl)-1*H*-benz[*d*]imidazole-5(6)-carbonitrile (**6j**)

Yield: 78%. M.p. 206.5 °C. ^1^H NMR (500 MHz, DMSO-*d*_6_): δ = 5.02 (2H, s, CH_2_), 6.92–6.95 (3H, m, aromatic CH), 7.40–7.41 (2H, m, aromatic CH), 7.82 (1H, s, aromatic CH), 8.02–8.08 (6H, m, aromatic CH), 8.19–8.22 (3H, m, aromatic CH). ^13^C NMR (125 MHz, DMSO-*d*_6_): δ (ppm) 30.70, 117.12, 117.74, 118.42, 120.54, 122.22, 123.56, 124.49, 125.52, 126.75, 128.17, 129.23, 129.36, 129.56, 129.89, 130.43, 130.63, 132.13, 132.80, 134.27, 136.89, 139.07, 139.31, 142.77, 145.92, 180.34. [M + H]^+^/2 calcd for C_30_H_18_N_6_OSCl_2_: 291.0393; found: 291.0385. Anal. calcd for C_30_H_18_N_6_OSCl_2_, C, 61.97; H, 3.12; N, 14.45. Found: C, 62.18; H, 3.11; N, 14.47.

##### 4.1.6.11 2-(4-(5-((2-(3-Nitrophenyl)-2-oxoethyl)thio)-4-phenyl-4*H*-1,2,4-triazol-3-yl)phenyl)-1*H*-benz[*d*]imidazole-5(6)-carbonitrile (**6k**)

Yield: 71%. M.p. >300 °C. ^1^H NMR (500 MHz, DMSO-*d*_6_): δ = 4.84 (2H, s, CH_2_), 6.95 (3H, d, *J* = 8.31 Hz, aromatic CH), 7.40 (2H, d, *J* = 8.34 Hz, aromatic CH), 7.60 (2H, d, *J* = 8.37 Hz, aromatic CH), 7.69 (2H, d, *J* = 8.25 Hz, aromatic CH), 7.77 (2H, s, aromatic CH), 7.90 (2H, d, *J* = 8.34 Hz, aromatic CH), 8.05 (3H, d, *J* = 8.37 Hz, aromatic CH). ^13^C NMR (125 MHz, DMSO-*d*_6_): δ (ppm) 30.03, 104.66, 117.21, 117.77, 120.40, 121.32, 122.10, 124.10, 124.20, 126.46, 127.64, 127.79, 128.13, 128.22, 129.51, 129.81, 130.41, 130.95, 131.03, 131.37, 135.82, 135.99, 148.27, 160.63, 166.03, 179.75. [M + H]^+^/2 calcd for C_30_H_19_N_7_O_3_S: 279.5708; found: 279.5698. Anal. calcd for C_30_H_19_N_7_O_3_S, C, 64.62; H, 3.43; N, 17.58. Found: C, 64.70; H, 3.44; N, 17.60.

##### 4.1.6.12 2-(4-(5-((2-Oxo-2-phenylethyl)thio)-4-phenyl-4*H*-1,2,4-triazol-3-yl)phenyl)-1*H*-benz[*d*]imidazole-5(6)-carbonitrile (**6l**)

Yield: 73%. M.p. 187.4 °C. ^1^H NMR (500 MHz, DMSO-*d*_6_): δ = 4.95 (2H, s, CH_2_), 7.07–7.08 (1H, m, aromatic CH), 7.10–7.11 (1H, m, aromatic CH), 7.19 (1H, s, aromatic CH), 7.22 (1H, s, aromatic CH), 7.36–7.38 (4H, m, aromatic CH), 7.42–7.43 (1H, m, aromatic CH), 7.45–7.46 (1H, m, aromatic CH), 7.78 (1H, s, aromatic CH), 7.81 (1H, s, aromatic CH), 7.86–7.90 (3H, m, aromatic CH), 7.93 (2H, d, *J* = 8.25 Hz, aromatic CH). ^13^C NMR (125 MHz, DMSO-*d*_6_): δ (ppm) 30.39, 117.18, 117.97, 119.68, 120.46, 124.13, 126.64, 127.50, 128.27, 128.91, 129.02, 129.34, 129.40, 129.48, 129.71, 130.63, 133.32, 134.10, 135.59, 138.42, 138.97, 140.05, 143.55, 178.98. [M + H]^+^/2 calcd for C_30_H_20_N_6_OS: 257.0782; found: 257.0773. Anal. calcd for C_30_H_20_N_6_OS, C, 70.30; H, 3.93; N, 16.40. Found: C, 70.42 H, 3.92; N, 16.44.

### 4.2 In Vitro Antifungal Activity

The antifungal activity of final compounds (**6a**–**6l**) was screened against four fungal strains according to the standard procedure of CLSI as described in the previous study.^44^*C. albicans* (ATCC 24433), *C. krusei* (ATCC 6258), *C. parapsilopsis* (ATCC 22019), and *C. glabrata* (ATCC 9) were used to test the antifungal activity of the final compounds. Voriconazole and fluconazole (against candida strains) were used as standard reference drugs.

### 4.3 Cytotoxicity Assay

The effect of the compounds **6a**–**6l** on the viability of L929 cell lines was analyzed by MTT assay as described in the previous work.^45^

### 4.4 Scanning Electron Microscopy with Energy-Dispersive X-ray Analysis (SEM-EDX)

Scanning electron microscopy (SEM) was used to observe the surface morphology of *C. glabrata* before and after the test-compound treatment. SEM images at a magnification of ×1000 and 10,000 are obtained using a ZEISS/Supra 40 VP at 10 kV of voltage. The procedure explained by Donelli et al.^46^ is modified in this study. To observe the activity of compounds on the fungal morphology, *C. glabrata* (10^8^ CFU/mL) was inoculated into the tubes containing desired concentrations (0.97, 1.95, and 3.90 μg/mL) of the most active compounds (**6b**, **6i**, and **6j**), standard drugs, and MHB. After incubation at 37 °C for 48 h, the tubes were centrifuged at 5000 rpm and the pellet was washed with sterile distilled water. The yeast cells in the pellet were fixed on the stubs, dried at 37 °C, and coated with gold as described by Jalal et al.^47^

### 4.5 Molecular Docking

All docking studies on various enzymes were performed using the Schrodinger Maestro Suite program. The interfaces of this program are used for the protein preparation process, ligand preparation process, grid generation, docking, and visualization studies.^48−50^ The crystal structure of the lanosterol 14α-demethylase enzyme was retrieved from the Protein Data Bank server (PDB code: 5TZ1). All ligands were set to the physiological pH (pH = 7.4) at the protonation step. The methodology for preparation of the protein structure was applied according to the previous study.^51^

## Supporting Information Available

^1^H NMR, ^13^C NMR, and HRMS spectra of compounds **6a**–**6l** (PDF)
