# Supplementary material for: Synthesis of Benzimidazole-1,2,4-triazole Derivatives as Potential Antifungal Agents Targeting 14α-Demethylase
Source: ACS Omega. 2023 Jan 19;8(4):4369–84. doi: 10.1021/acsomega.2c07755 (PMC9893751; doi:10.1021/acsomega.2c07755)
Supplement: Supplementary file 1 — ao2c07755_si_001.pdf [file ao2c07755_si_001.pdf]

Synthesis of benzimidazole-1,2,4-triazole derivatives as potential antifungal agents targeting 14 α-demethylase

Emir Güzel[1], Ulviye Acar Çevik*[2], Asaf Evrim Evren[3], Hayrani Eren Bostancı[4], Ülküye Dudu Gül[5], Uğur Kayış[6], Yusuf Özkay[2], Zafer Asım Kaplancıklı[2]

[1] Department of Pharmaceutical Chemistry, Faculty of Pharmacy, Biruni University, İstanbul, Turkey.

[2] Department of Pharmaceutical Chemistry, Faculty of Pharmacy, Anadolu University, Eskişehir 26470, Turkey.

[3] Department of Pharmacy Services, Vocational School of Health Services, Bilecik Şeyh Edebali University, 11000, Bilecik, Turkey.

[4] Department of Biochemistry, Faculty of Pharmacy, Sivas Cumhuriyet University, Sivas, Turkey.

[5]Department of Bioengineering, Faculty of Engineering, Bilecik Seyh Edebali University, Bilecik, Turkey.

[6] Pazaryeri Vocational School, Program of Pharmacy Services, Bilecik Şey Edebali University, 11230 Bilecik, Turkey.

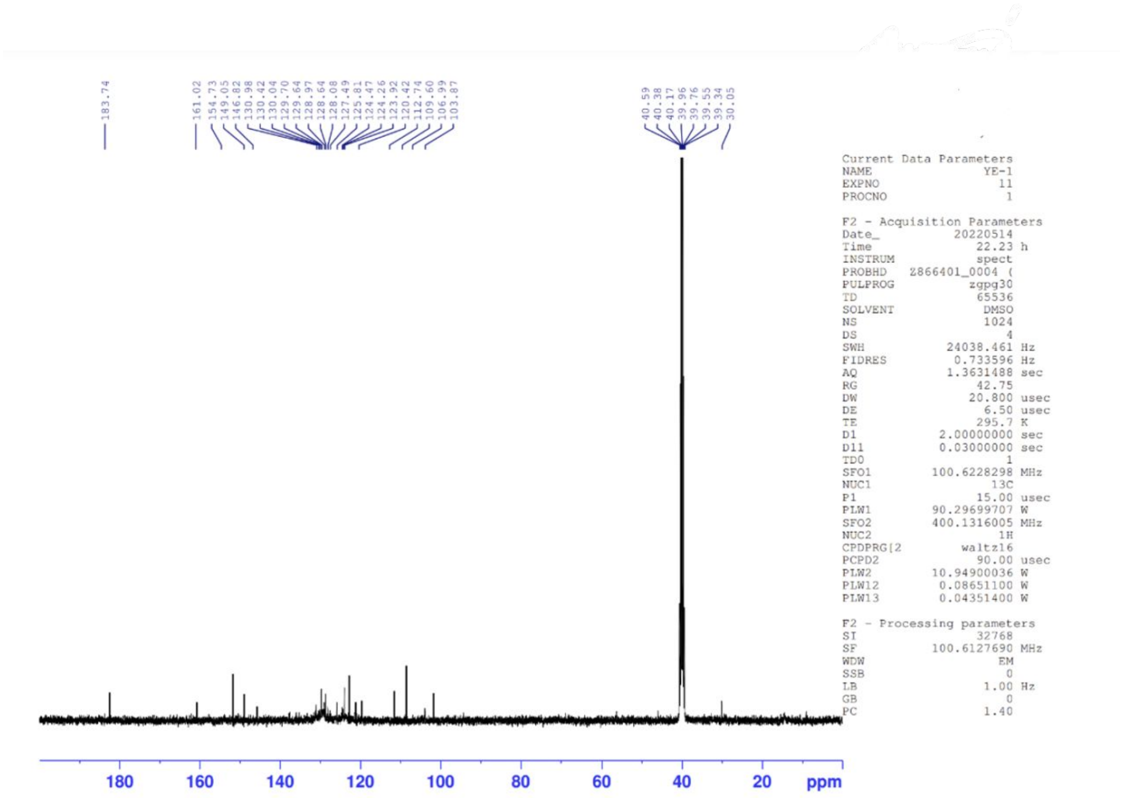

Figure S1. ^{1}H-NMR spectrum of compound 6a

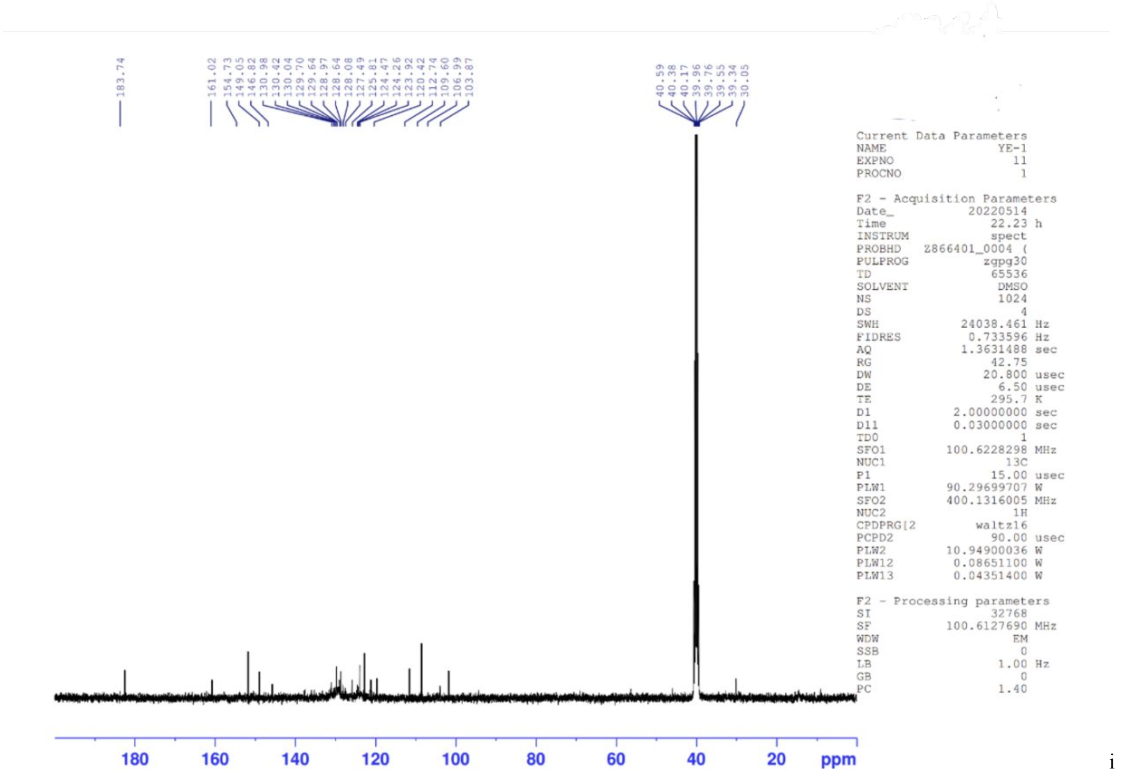

i

Figure S2. ^{13}C-NMR spectrum of compound 6a

Data File: C:\LabSolutions\Data\Analiz\uac\YE-1_150.lcd

Elmt	Val.	Min	Max	Elmt	Val.	Min	Max	Elmt	Val.	Min	Max	Elmt	Val.	Min	Max	Use Adduct
H	1	9	25	O	2	0	3	S	2	1	1	Ru	2	0	0	H
C	4	7	35	F	1	0	0	Cl	1	0	0	Pd	2	0	0	Na
N	3	7	7	P	3	0	0	Br	1	0	0	I	3	0	0	NH4

Error Margin (ppm): 5
HC Ratio: unlimited
Max Isotopes: 3
MSn Iso RI (%): 10.00

DBE Range: 5.0 - 30.0
Apply N Rule: yes
Isotope RI (%): 1.00
MSn Logic Mode: AND

Electron Ions: both
Use MSn Info: yes
Isotope Res: 9000
Max Results: 50

Event#: 1 MS(E+) Ret. Time : 2.867 Scan# : 431

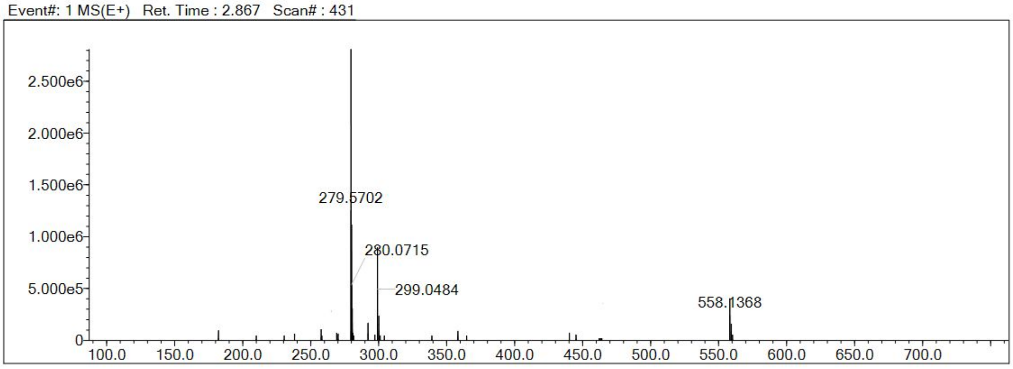

Measured region for 279.5702 m/z

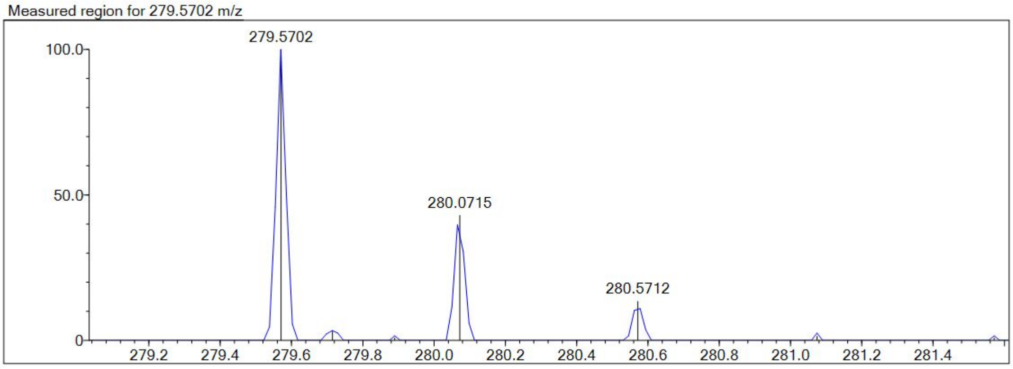

C30 H19 N7 O3 S [M+2H]2+ : Predicted region for 279.5708 m/z

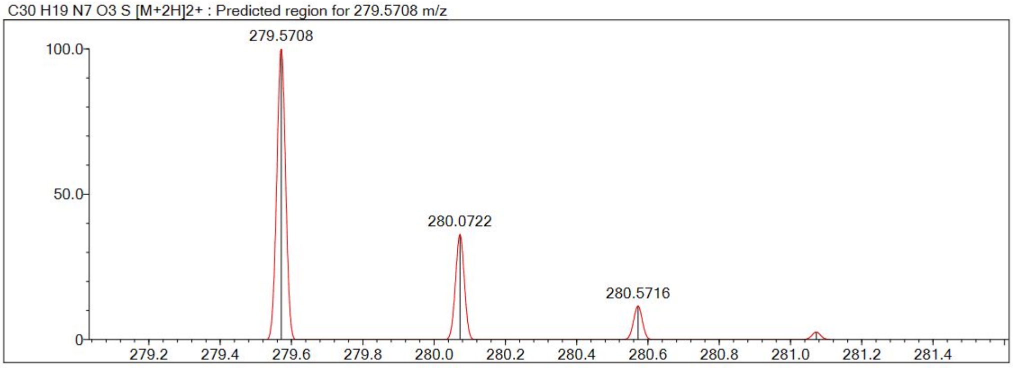

Rank	Score	Formula (M)	Ion	Meas. m/z	Pred. m/z	Df. (mDa)	Df. (ppm)	Iso	DBE
1	96.54	C30 H19 N7 O3 S	[M+2H]2+	279.5702	279.5708	-0.6	-2.15	99.39	25.0

Figure S3. Mass spectrum of compound 6a

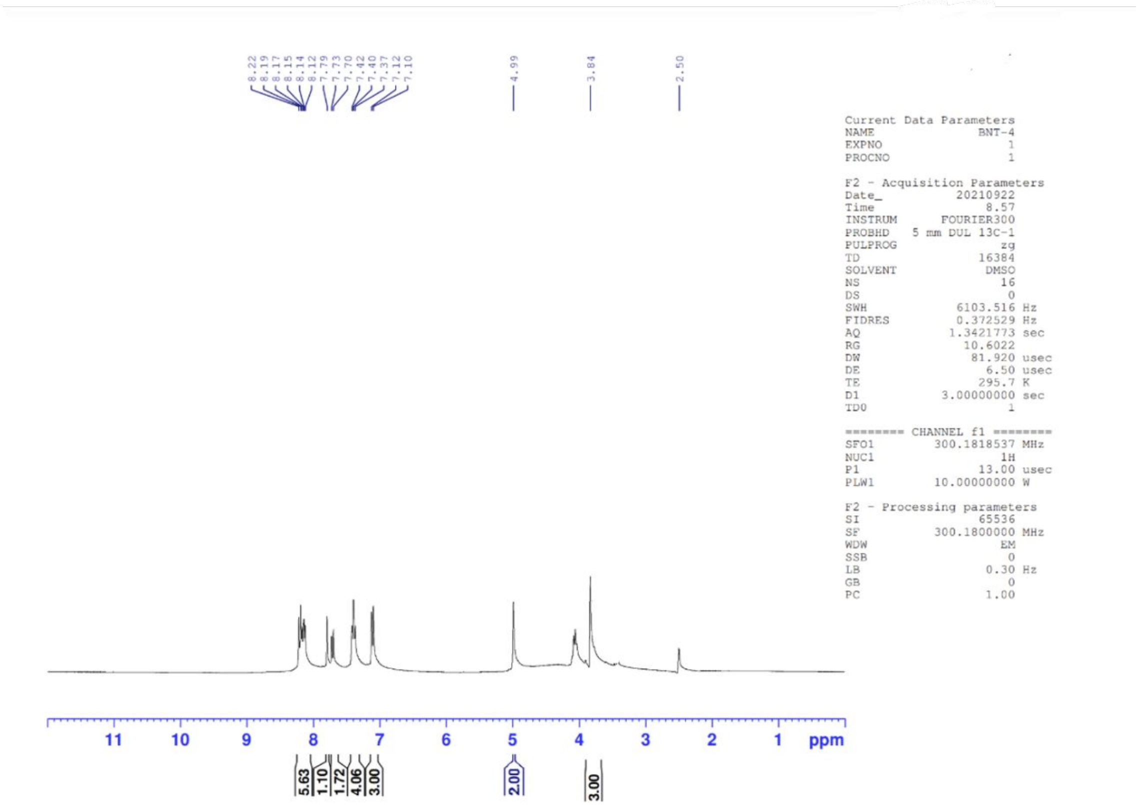

Figure S4. ^{1}H-NMR spectrum of compound 6b

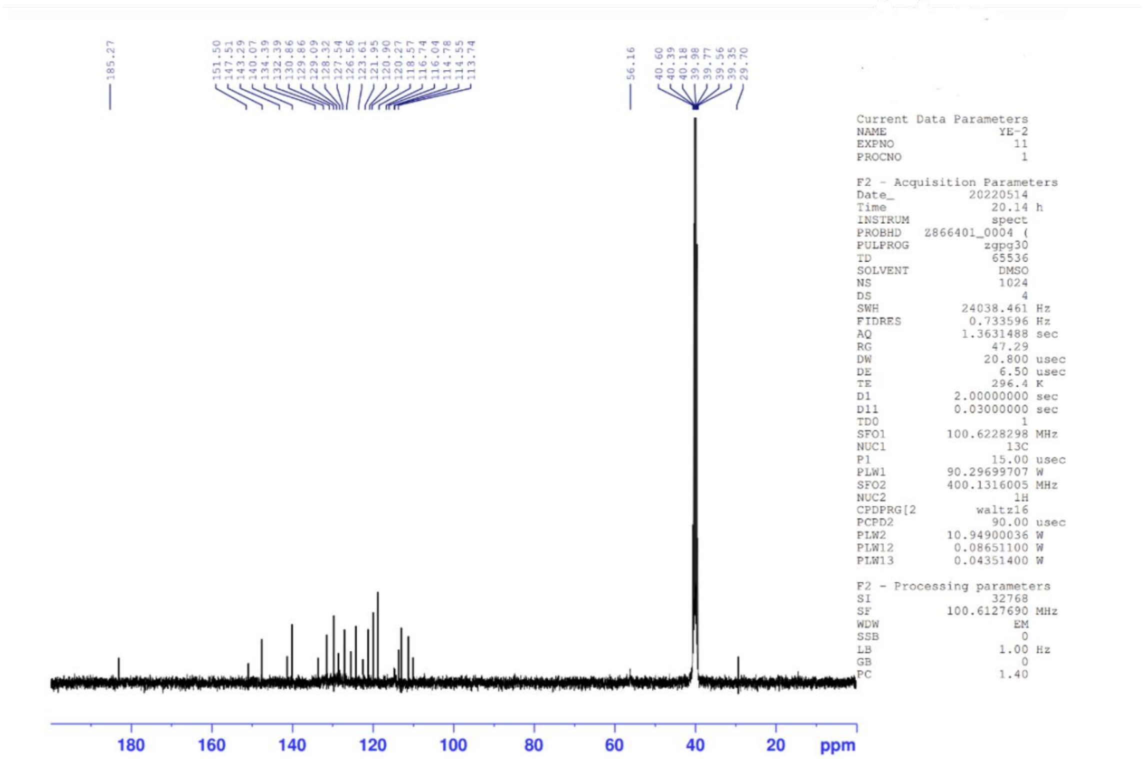

Figure S5. ^{13}C-NMR spectrum of compound 6b

Formula Predictor Report - YE-2_151.lcd Page 1 of 1

Data File: C:\LabSolutions\Data\Analiz\uac\YE-2_151.lcd

Elmt	Val.	Min	Max	Elmt	Val.	Min	Max	Elmt	Val.	Min	Max	Elmt	Val.	Min	Max	Use Adduct
H	1	9	25	O	2	0	3	S	2	1	1	Ru	2	0	0	H
C	4	7	35	F	1	0	0	Cl	1	0	0	Pd	2	0	0	Na
N	3	6	6	P	3	0	0	Br	1	0	0	I	3	0	0	NH4

Error Margin (ppm): 5
HC Ratio: unlimited
Max Isotopes: 3
MSn Iso RI (%): 10.00

DBE Range: 5.0 - 30.0
Apply N Rule: yes
Isotope RI (%): 1.00
MSn Logic Mode: AND

Electron Ions: both
Use MSn Info: yes
Isotope Res: 9000
Max Results: 50

Event#: 1 MS(E+) Ret. Time : 2.787 Scan# : 419

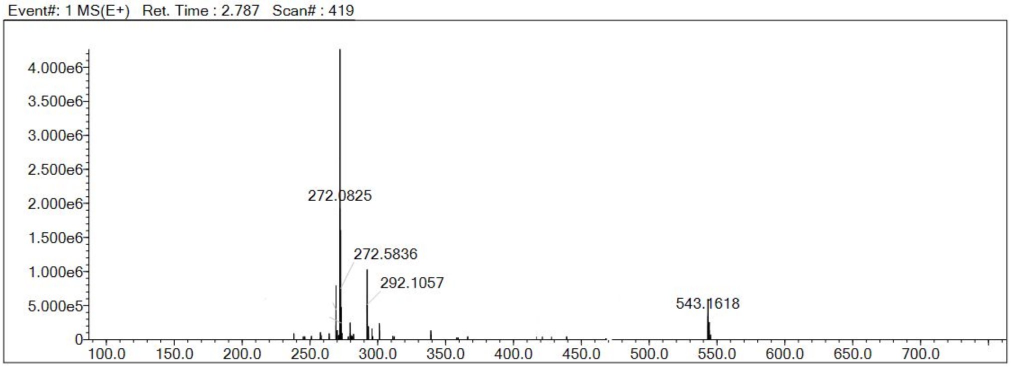

Measured region for 272.0825 m/z

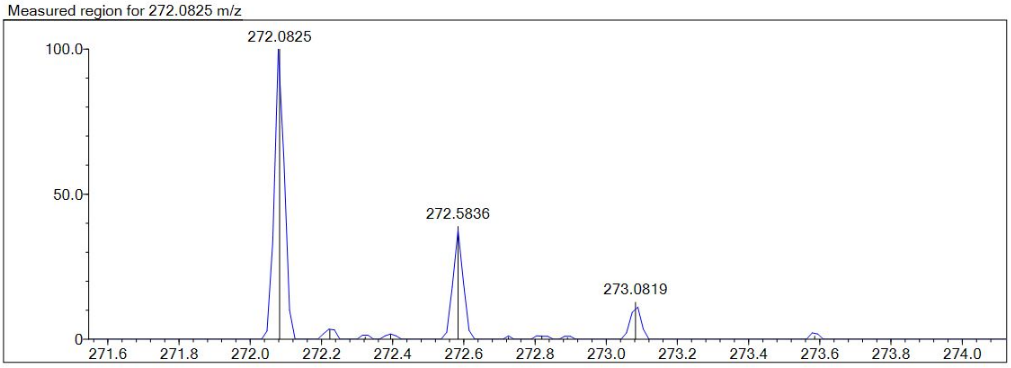

C31 H22 N6 O2 S [M+2H]2+ : Predicted region for 272.0835 m/z

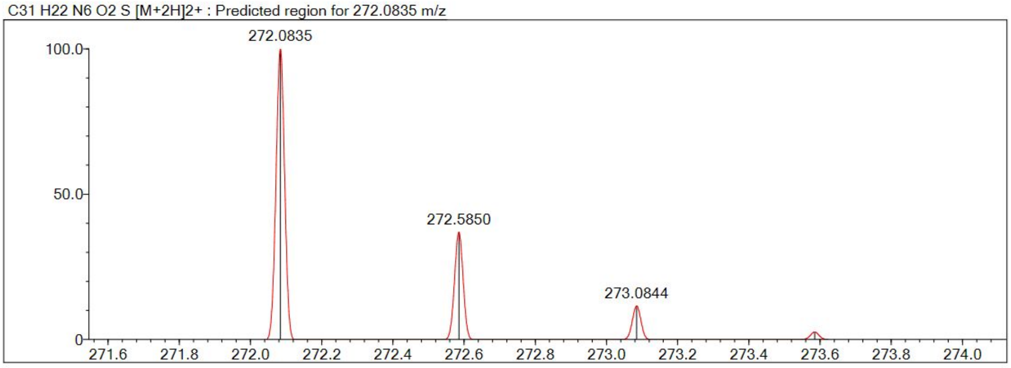

Rank	Score	Formula (M)	Ion	Meas. m/z	Pred. m/z	Df. (mDa)	Df. (ppm)	Iso	DBE
1	68.04	C31 H22 N6 O2 S	[M+2H]2+	272.0825	272.0835	-1.0	-3.68	72.92	24.0

Figure S6. Mass spectrum of compound 6b

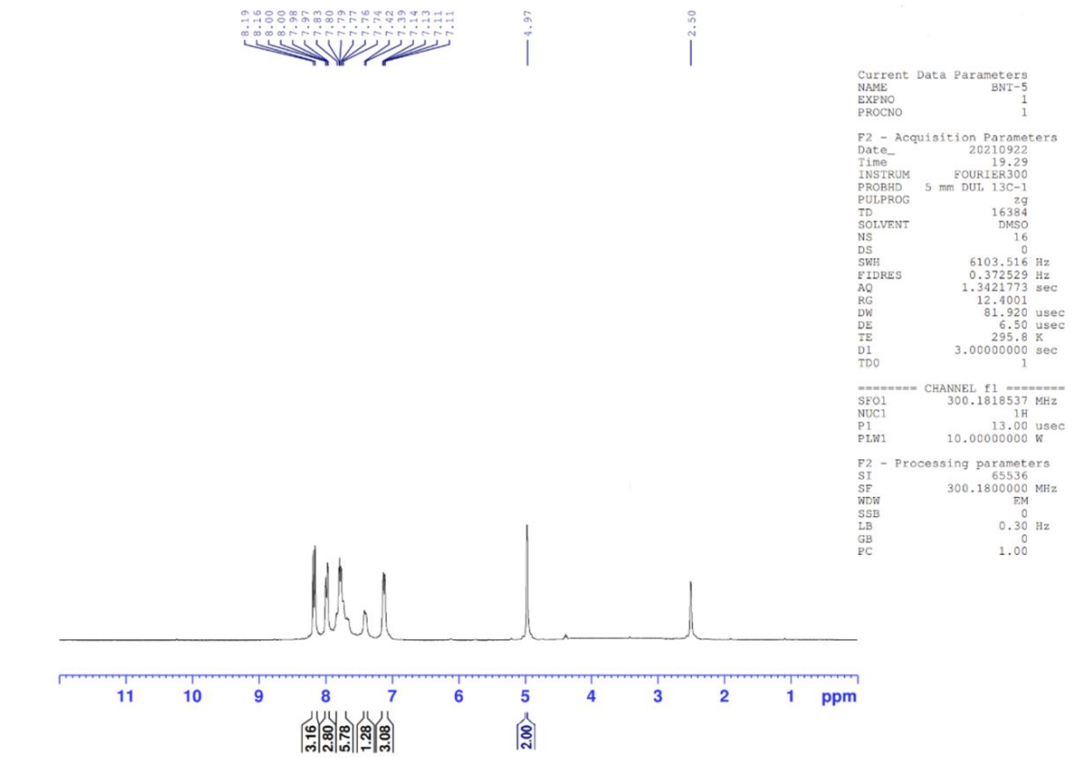

Figure S7. ^{1}H-NMR spectrum of compound 6c

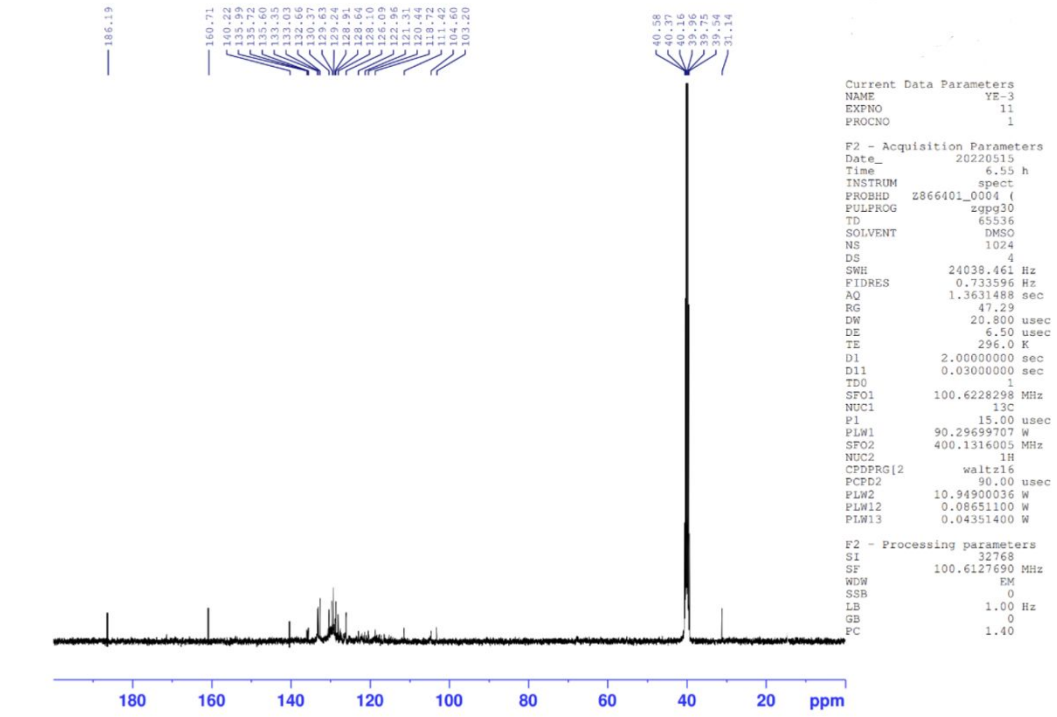

Figure S8. ^{13}C-NMR spectrum of compound 6c

Data File: C:\LabSolutions\Data\Analiz\uac\YE-3_152.lcd

Elmt	Val.	Min	Max	Elmt	Val.	Min	Max	Elmt	Val.	Min	Max	Elmt	Val.	Min	Max	Use Adduct
H	1	9	20	O	2	0	1	S	2	1	1	Ru	2	0	0	H
C	4	7	35	F	1	0	0	Cl	1	0	0	Pd	2	0	0	Na
N	3	7	7	P	3	0	0	Br	1	0	0	I	3	0	0	NH4

Error Margin (ppm):	5	DBE Range:	5.0 - 30.0	Electron Ions:	both
HC Ratio:	unlimited	Apply N Rule:	yes	Use MSn Info:	yes
Max Isotopes:	3	Isotope RI (%):	1.00	Isotope Res:	9000
MSn Iso RI (%):	10.00	MSn Logic Mode:	AND	Max Results:	50

Event#: 1 MS(E+) Ret. Time : 2.400 Scan# : 361

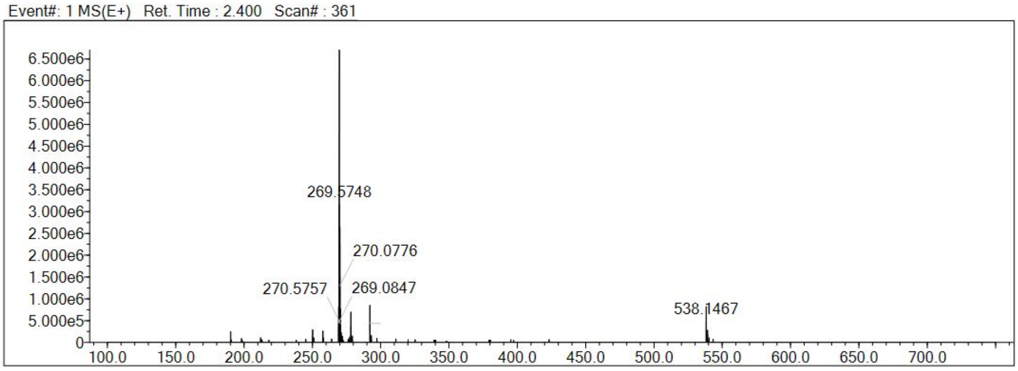

Measured region for 269.5748 m/z

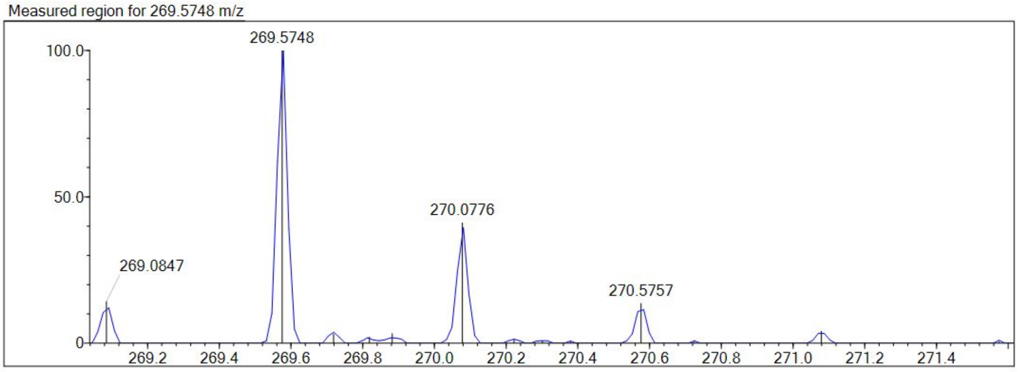

C31 H19 N7 O S [M+2H]2+ : Predicted region for 269.5759 m/z

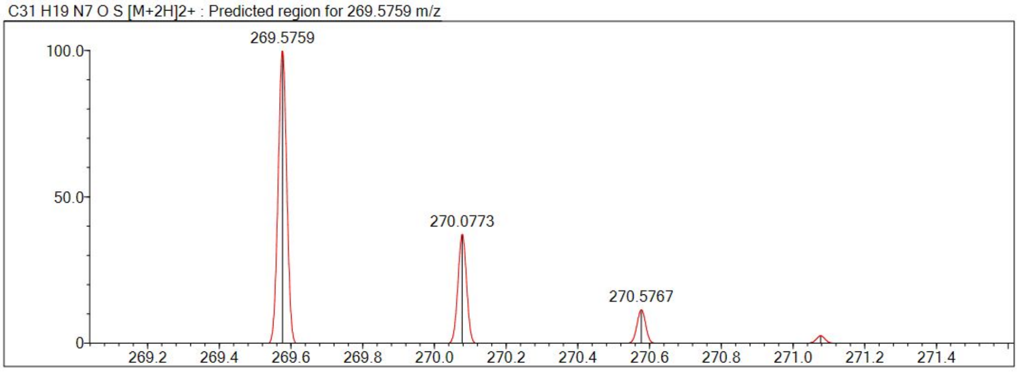

Rank	Score	Formula (M)	Ion	Meas. m/z	Pred. m/z	Df. (mDa)	Df. (ppm)	Iso	DBE
1	88.98	C31 H19 N7 O S	[M+2H]2+	269.5748	269.5759	-1.1	-4.08	96.40	26.0

Figure S9. Mass spectrum of compound 6c

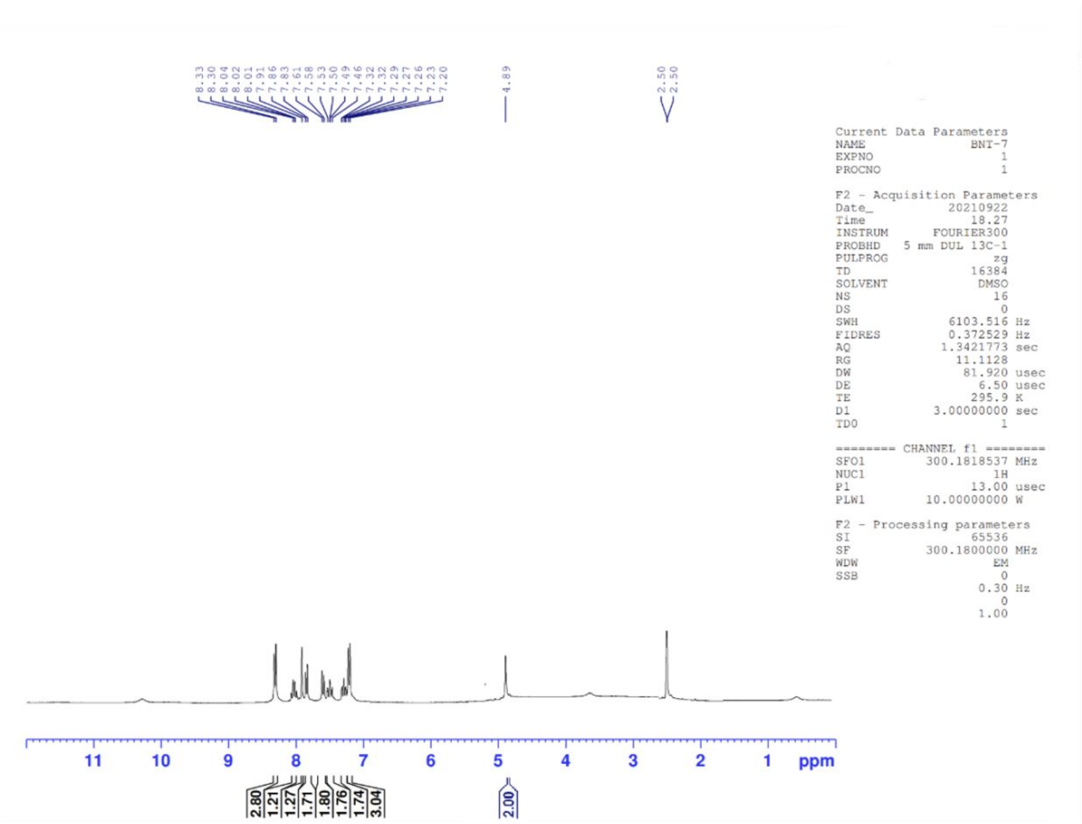

Figure S10. ^{1}H-NMR spectrum of compound 6d

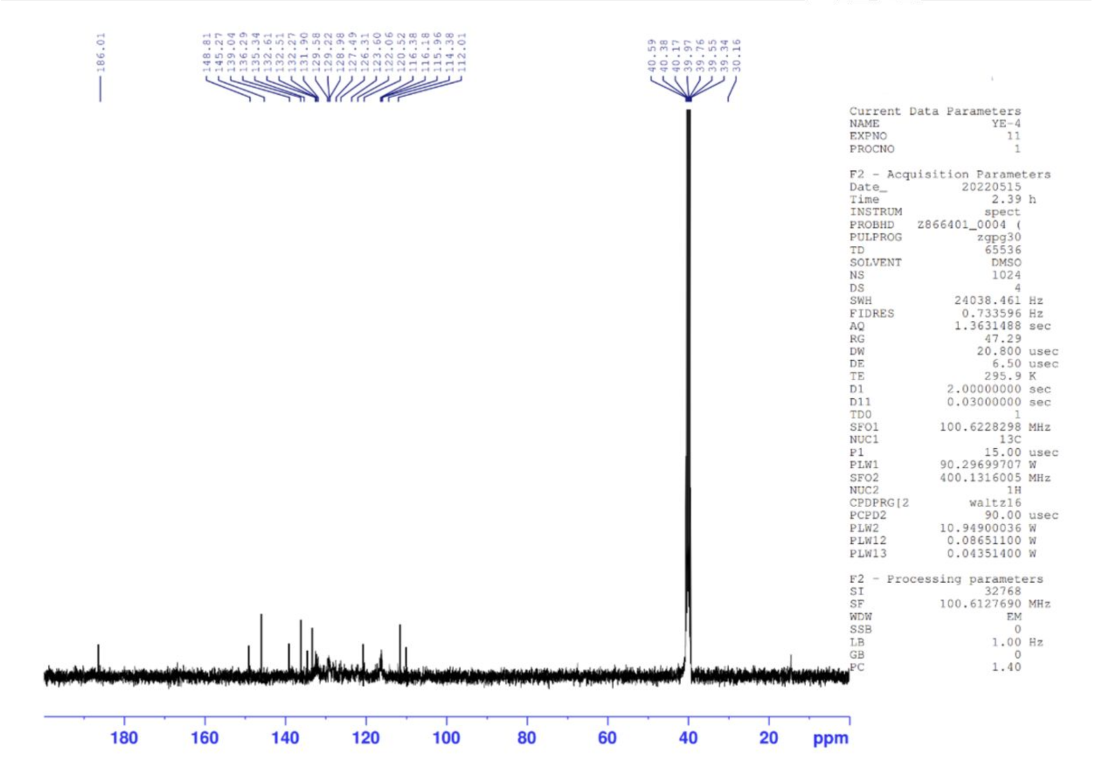

Figure S11. ^{13}C-NMR spectrum of compound 6d

Data File: C:\LabSolutions\Data\Analiz\uac\YE-4_153.lcd

Elmt	Val.	Min	Max	Elmt	Val.	Min	Max	Elmt	Val.	Min	Max	Elmt	Val.	Min	Max	Use Adduct
H	1	9	20	O	2	0	1	S	2	1	1	Ru	2	0	0	H
C	4	7	35	F	1	1	1	Cl	1	0	0	Pd	2	0	0	Na
N	3	6	6	P	3	0	0	Br	1	0	0	I	3	0	0	NH4

Error Margin (ppm): 5
HC Ratio: unlimited
Max Isotopes: 3
MSn Iso RI (%): 10.00

DBE Range: 5.0 - 30.0
Apply N Rule: yes
Isotope RI (%): 1.00
MSn Logic Mode: AND

Electron Ions: both
Use MSn Info: yes
Isotope Res: 9000
Max Results: 50

Event#: 1 MS(E+) Ret. Time : 2.787 Scan# : 419

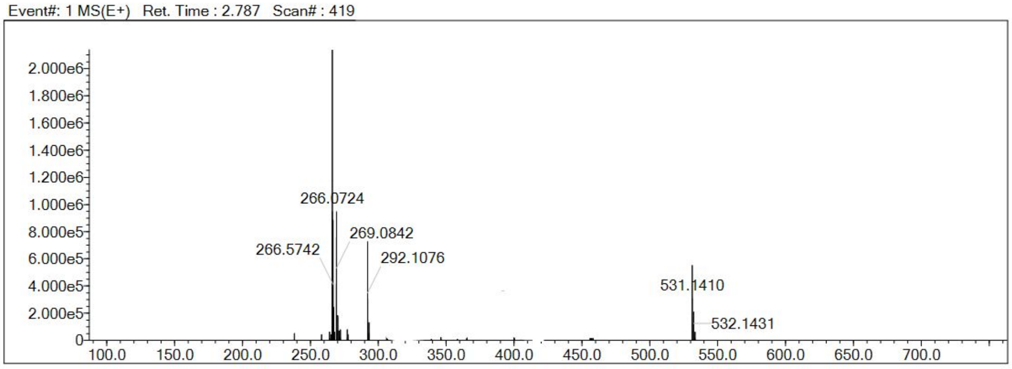

Measured region for 266.0724 m/z

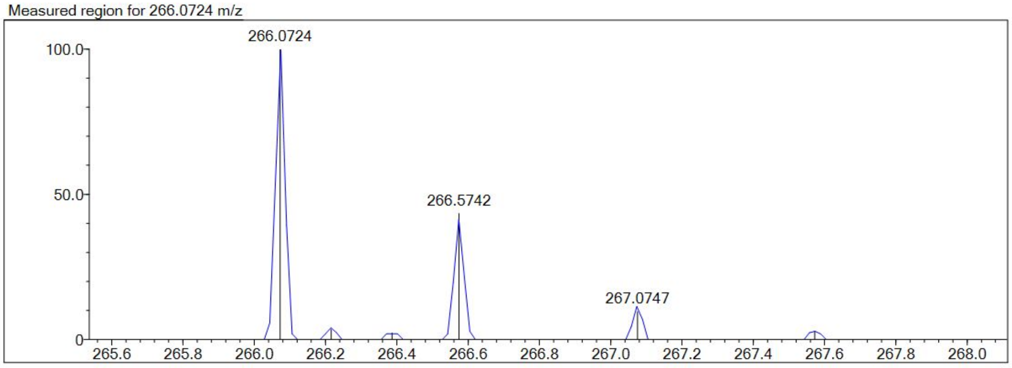

C30 H19 N6 O F S [M+2H]2+ : Predicted region for 266.0735 m/z

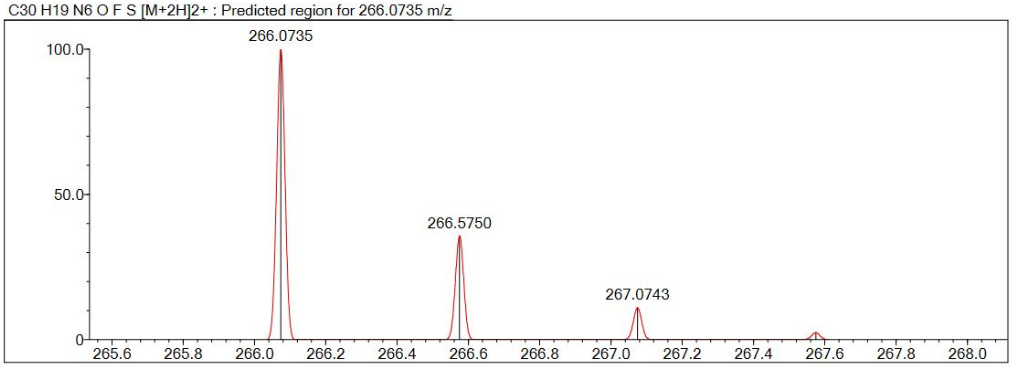

Rank	Score	Formula (M)	Ion	Meas. m/z	Pred. m/z	Df. (mDa)	Df. (ppm)	Iso	DBE
1	89.31	C30 H19 N6 O F S	[M+2H]2+	266.0724	266.0735	-1.1	-4.13	96.90	24.0

Figure S12. Mass spectrum of compound 6d

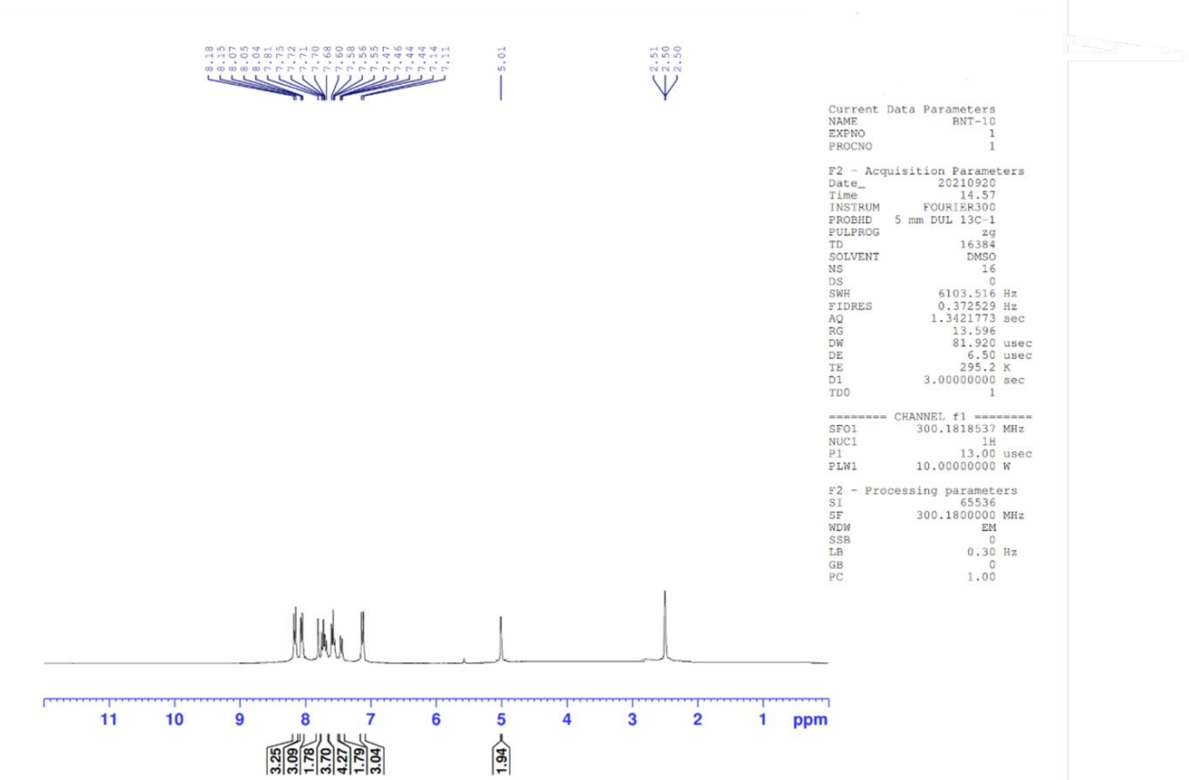

Figure S13. ^{1}H-NMR spectrum of compound 6e

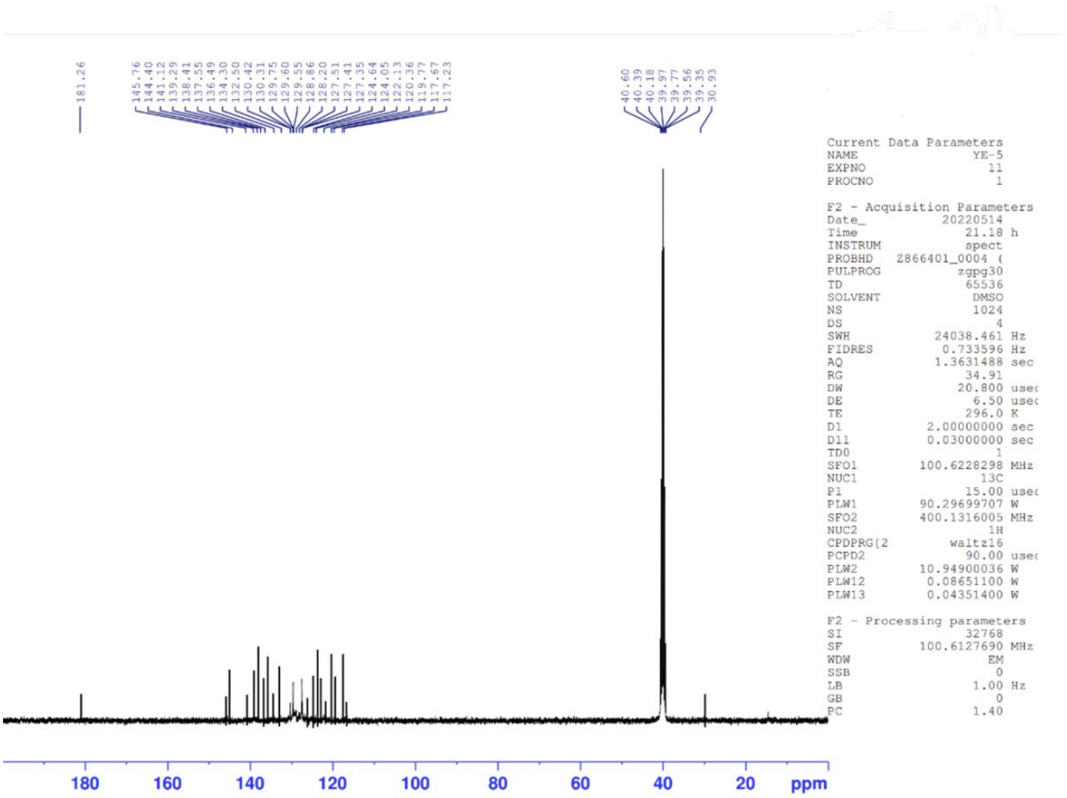

Figure S14. ^{13}C-NMR spectrum of compound 6e

Data File: C:\LabSolutions\Data\Analiz\uac\YE-5_154.lcd

Elmt	Val.	Min	Max	Elmt	Val.	Min	Max	Elmt	Val.	Min	Max	Elmt	Val.	Min	Max	Use Adduct
H	1	9	25	O	2	0	1	S	2	1	1	Ru	2	0	0	H
C	4	7	37	F	1	0	0	Cl	1	0	0	Pd	2	0	0	Na
N	3	6	6	P	3	0	0	Br	1	0	0	I	3	0	0	NH4

Error Margin (ppm):	5	DBE Range:	5.0 - 30.0	Electron Ions:	both
HC Ratio:	unlimited	Apply N Rule:	yes	Use MSn Info:	yes
Max Isotopes:	3	Isotope RI (%):	1.00	Isotope Res:	9000
MSn Iso RI (%):	10.00	MSn Logic Mode:	AND	Max Results:	50

Event#: 1 MS(E+) Ret. Time : 3.293 -> 4.120 Scan# : 495 -> 619

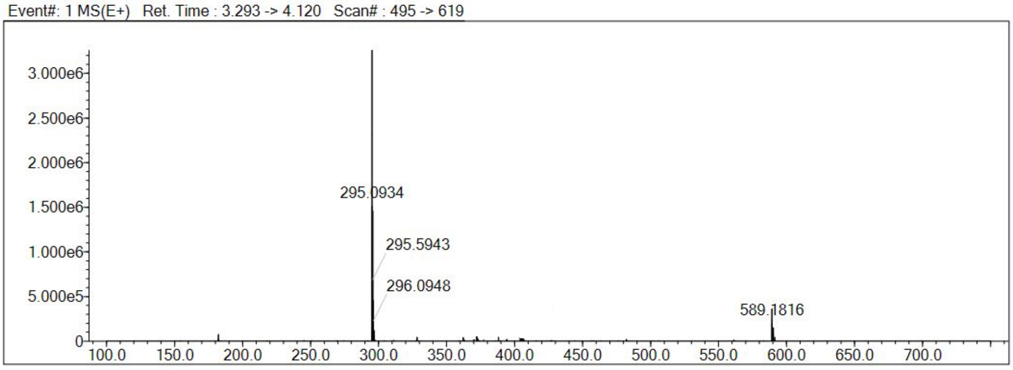

Measured region for 295.0934 m/z

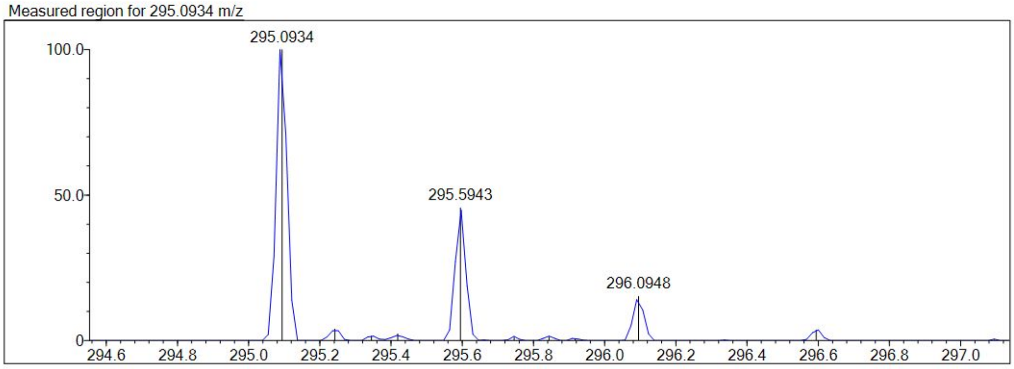

C36 H24 N6 O S [M+2H]2+ : Predicted region for 295.0939 m/z

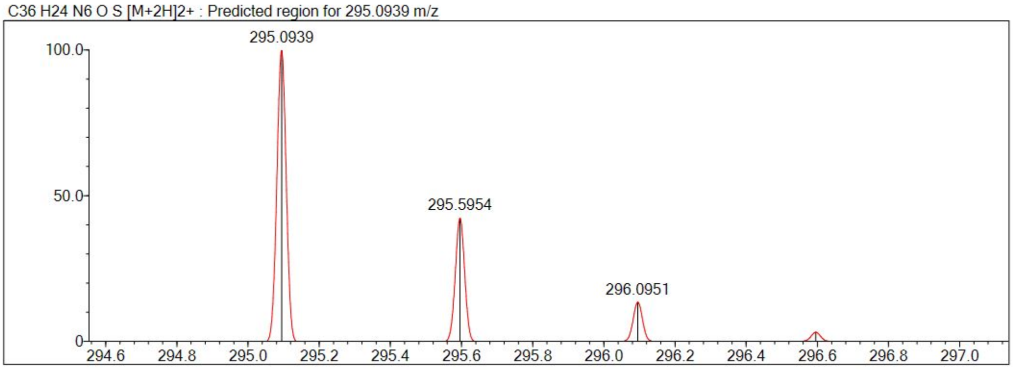

Rank	Score	Formula (M)	Ion	Meas. m/z	Pred. m/z	Df. (mDa)	Df. (ppm)	Iso	DBE
1	77.05	C36 H24 N6 O S	[M+2H]2+	295.0934	295.0939	-0.5	-1.69	78.40	28.0

Figure S15. Mass spectrum of compound 6e

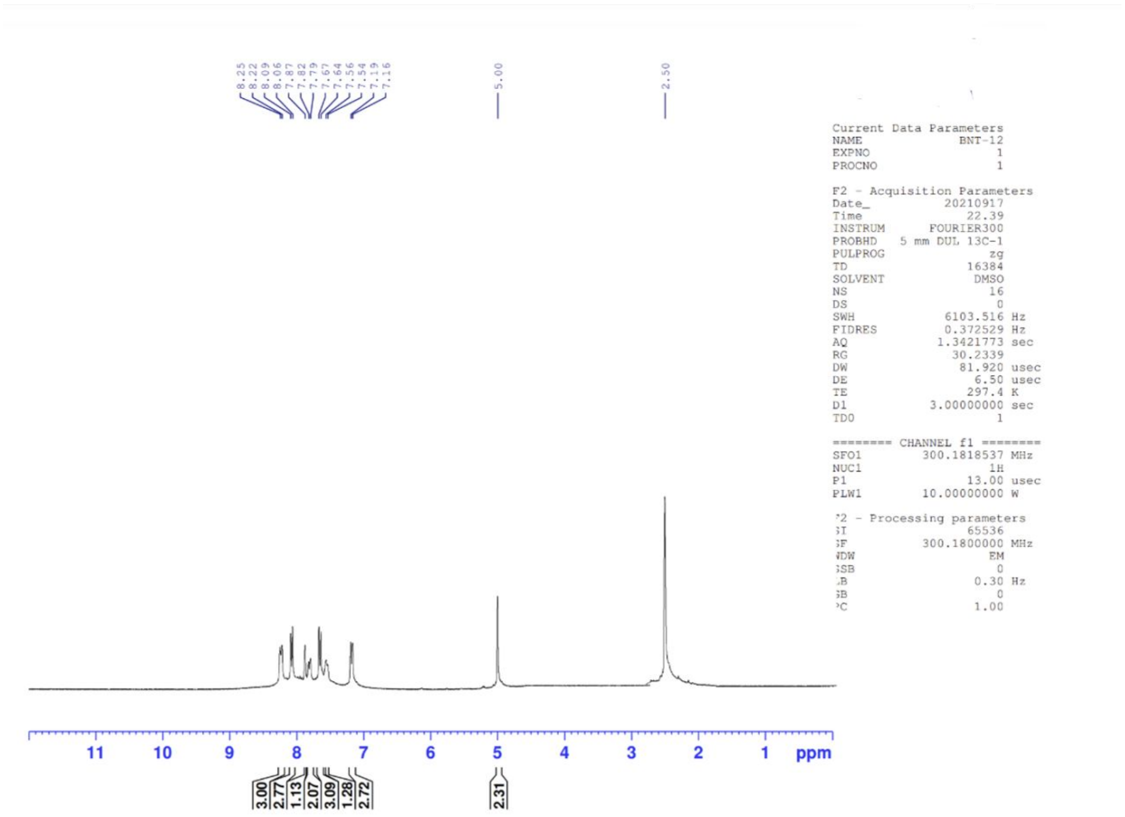

Figure S16. ^{1}H-NMR spectrum of compound 6f

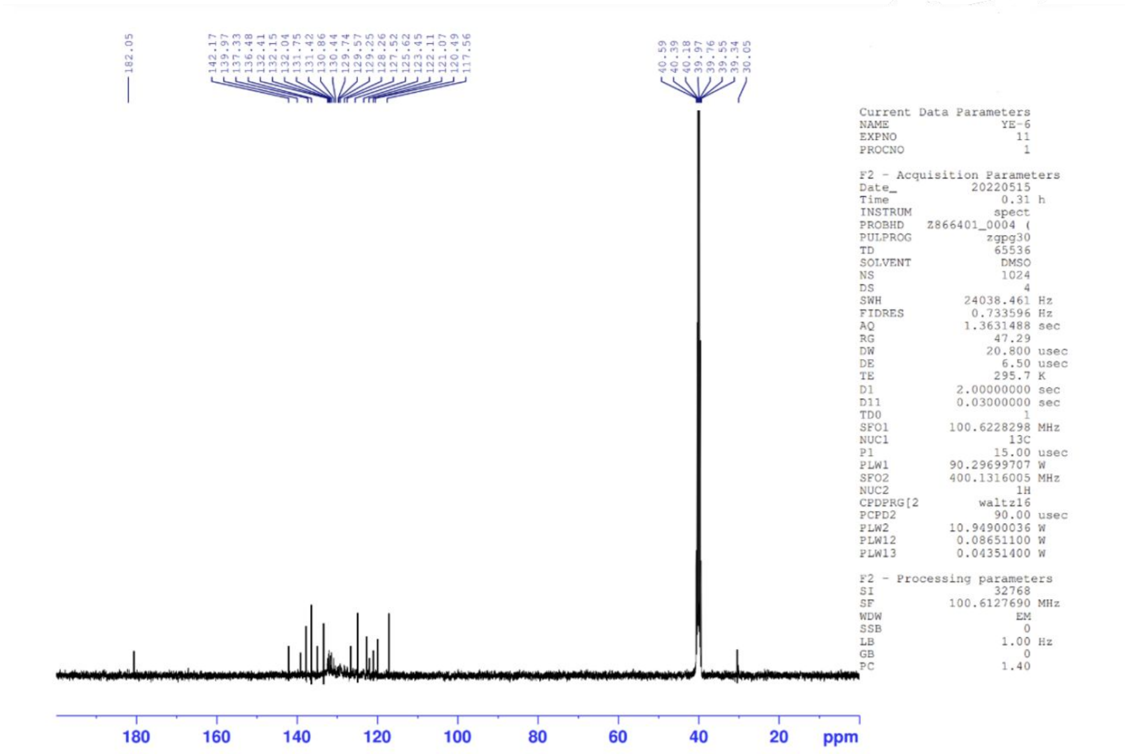

Figure S17. ^{13}C-NMR spectrum of compound 6f

Data File: C:\LabSolutions\Data\Analiz\uac\YE-6_155.lcd

Elmt	Val.	Min	Max	Elmt	Val.	Min	Max	Elmt	Val.	Min	Max	Elmt	Val.	Min	Max	Use Adduct
H	1	9	20	O	2	0	1	S	2	1	1	Ru	2	0	0	H
C	4	7	37	F	1	0	0	Cl	1	0	0	Pd	2	0	0	Na
N	3	6	6	P	3	0	0	Br	1	1	1	I	3	0	0	NH4

Error Margin (ppm): 5
HC Ratio: unlimited
Max Isotopes: 3
MSn Iso RI (%): 10.00

DBE Range: 5.0 - 30.0
Apply N Rule: yes
Isotope RI (%): 1.00
MSn Logic Mode: AND

Electron Ions: both
Use MSn Info: yes
Isotope Res: 9000
Max Results: 50

Event#: 1 MS(E+) Ret. Time : 3.307 -> 3.373 Scan# : 497 -> 507

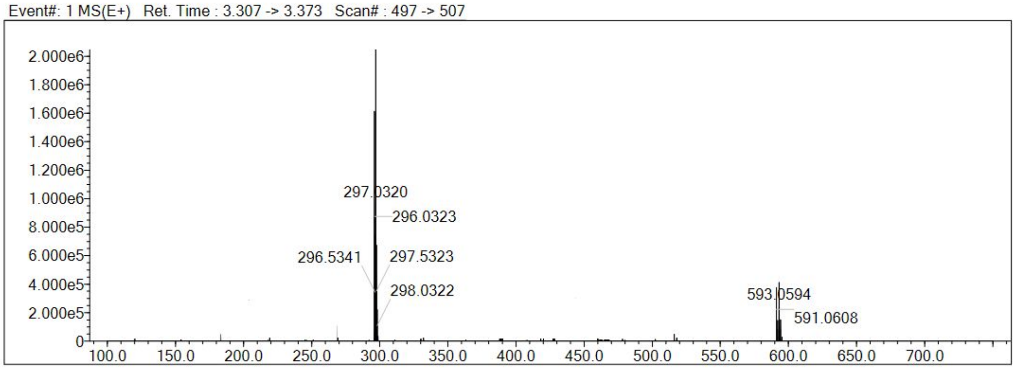

Measured region for 296.0323 m/z

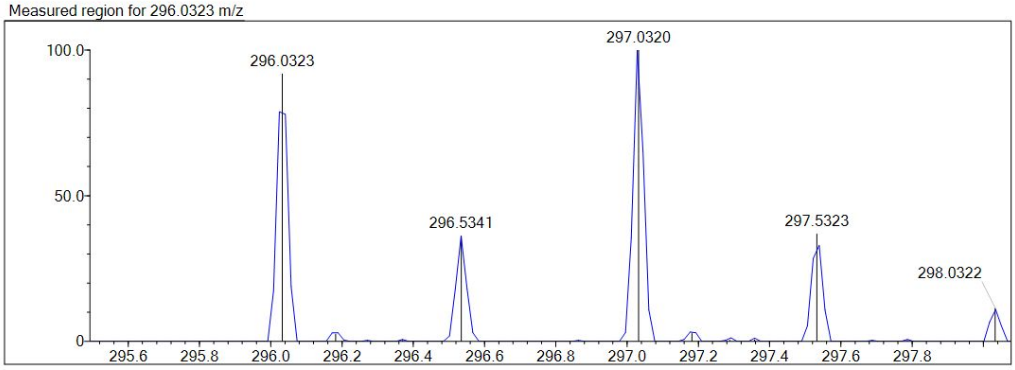

C30 H19 N6 O S Br [M+2H]2+ : Predicted region for 296.0335 m/z

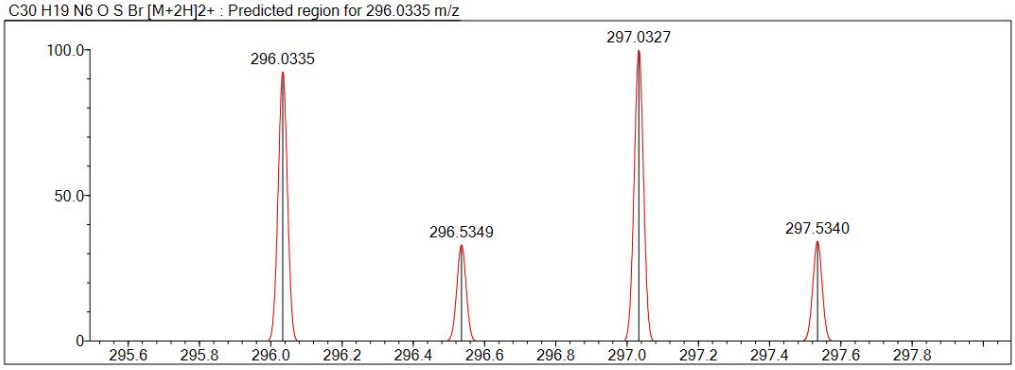

Rank	Score	Formula (M)	Ion	Meas. m/z	Pred. m/z	Df. (mDa)	Df. (ppm)	Iso	DBE
1	62.84	C30 H19 N6 O S Br	[M+2H]2+	296.0323	296.0335	-1.2	-4.05	68.03	24.0

Figure S18. Mass spectrum of compound 6f

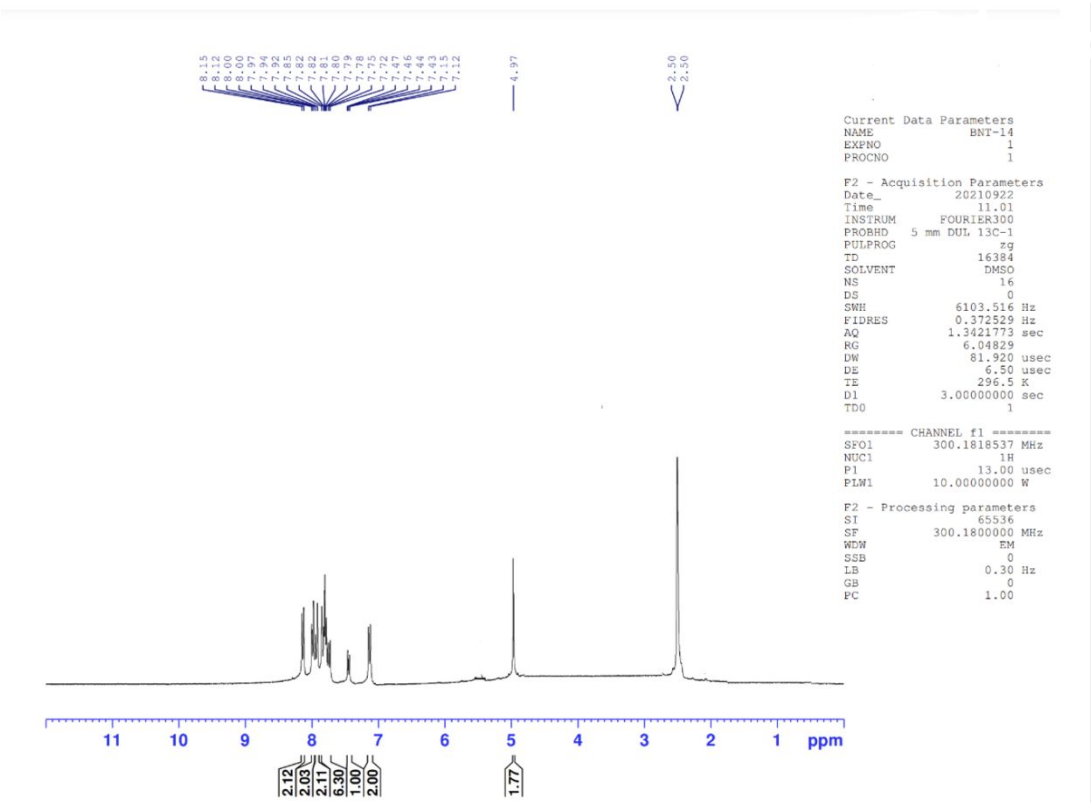

Figure S19. ^{1}H-NMR spectrum of compound 6g

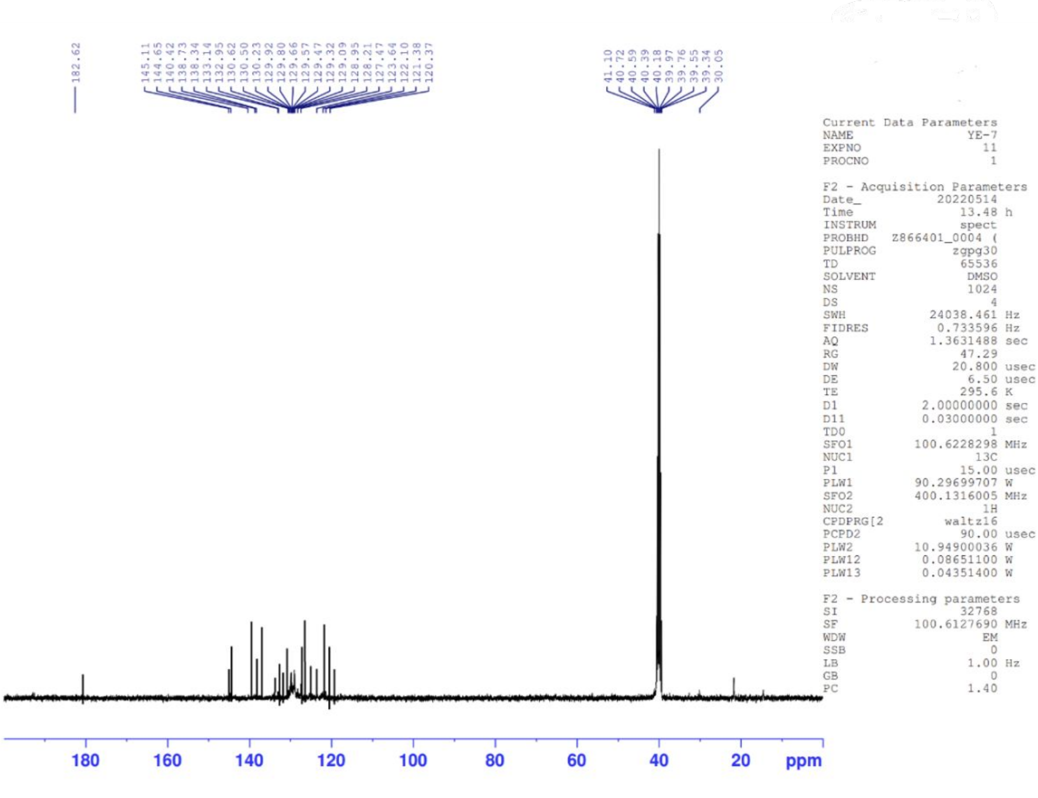

Figure S20. ^{13}C-NMR spectrum of compound 6g

Formula Predictor Report - YE-8_157.lcd | Page 1 of 1

Data File: C:\LabSolutions\Data\Analiz\uac\YE-8_157.lcd

Elmt	Val.	Min	Max	Elmt	Val.	Min	Max	Elmt	Val.	Min	Max	Elmt	Val.	Min	Max	Use Adduct
H	1	9	18	O	2	0	1	S	2	1	1	Ru	2	0	0	H
C	4	7	37	F	1	0	0	Cl	1	2	2	Pd	2	0	0	Na
N	3	6	6	P	3	0	0	Br	1	0	0	I	3	0	0	NH4

Error Margin (ppm): 5
HC Ratio: unlimited
Max Isotopes: 3
MSn Iso RI (%): 10.00

DBE Range: 5.0 - 30.0
Apply N Rule: yes
Isotope RI (%): 1.00
MSn Logic Mode: AND

Electron Ions: both
Use MSn Info: yes
Isotope Res: 9000
Max Results: 50

Event#: 1 MS(E+) Ret. Time : 3.733 Scan# : 561

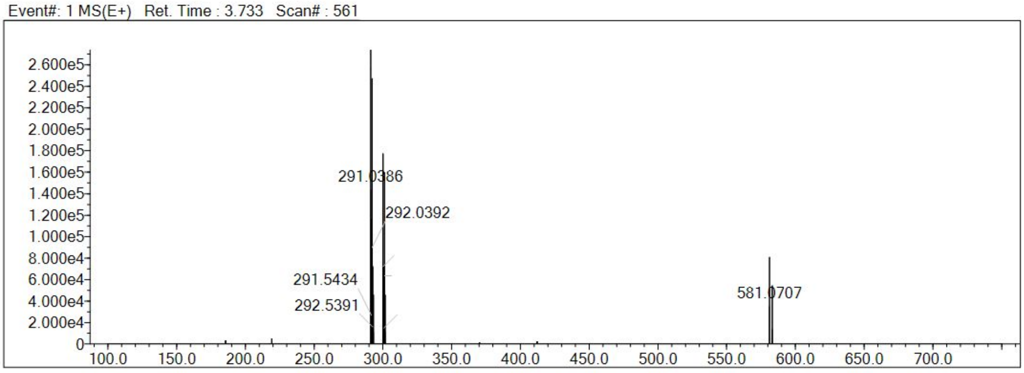

Measured region for 581.0707 m/z

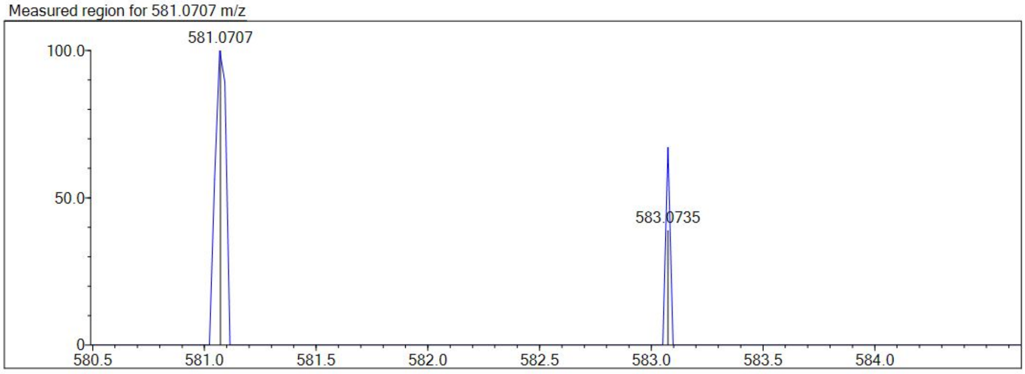

C30 H18 N6 O S Cl2 [M+H]+ : Predicted region for 581.0713 m/z

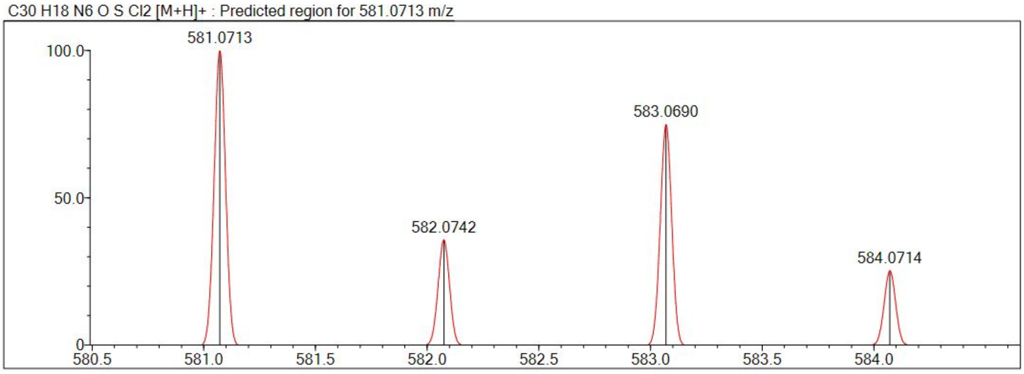

Rank	Score	Formula (M)	Ion	Meas. m/z	Pred. m/z	Df. (mDa)	Df. (ppm)	Iso	DBE
1	14.78	C30 H18 N6 O S Cl2	[M+H]+	581.0707	581.0713	-0.6	-1.03	14.79	24.0

Figure S21. Mass spectrum of compound 6g

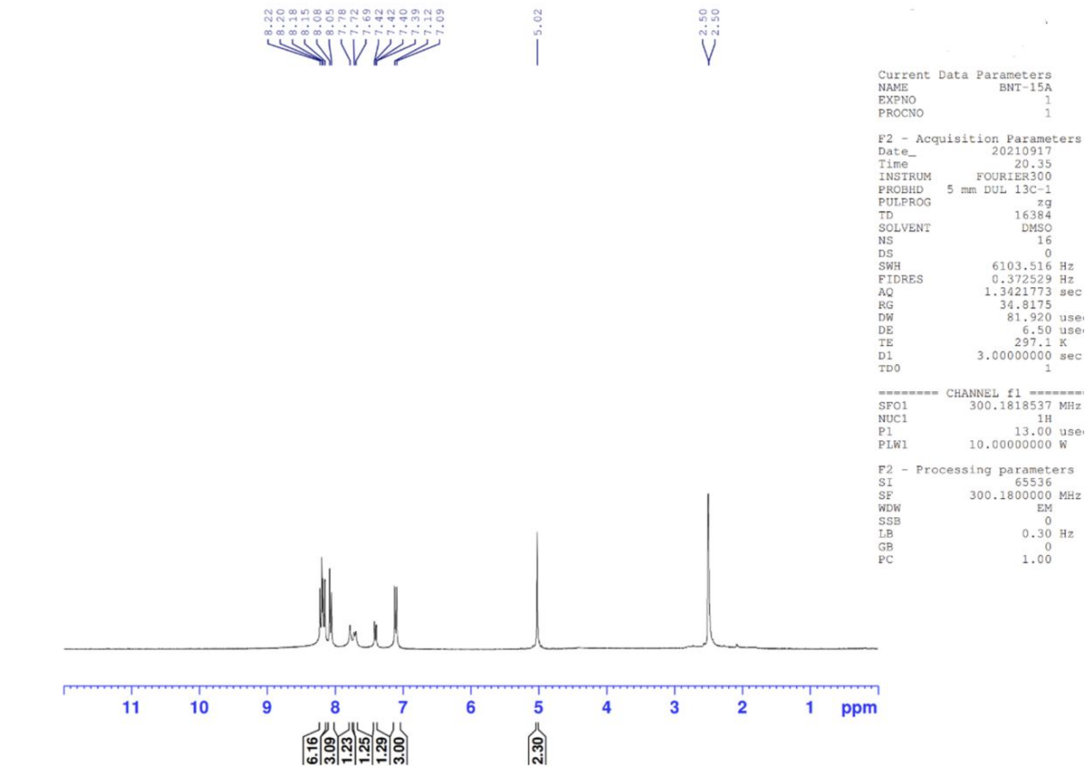

Figure S22. ¹H-NMR spectrum of compound 6h

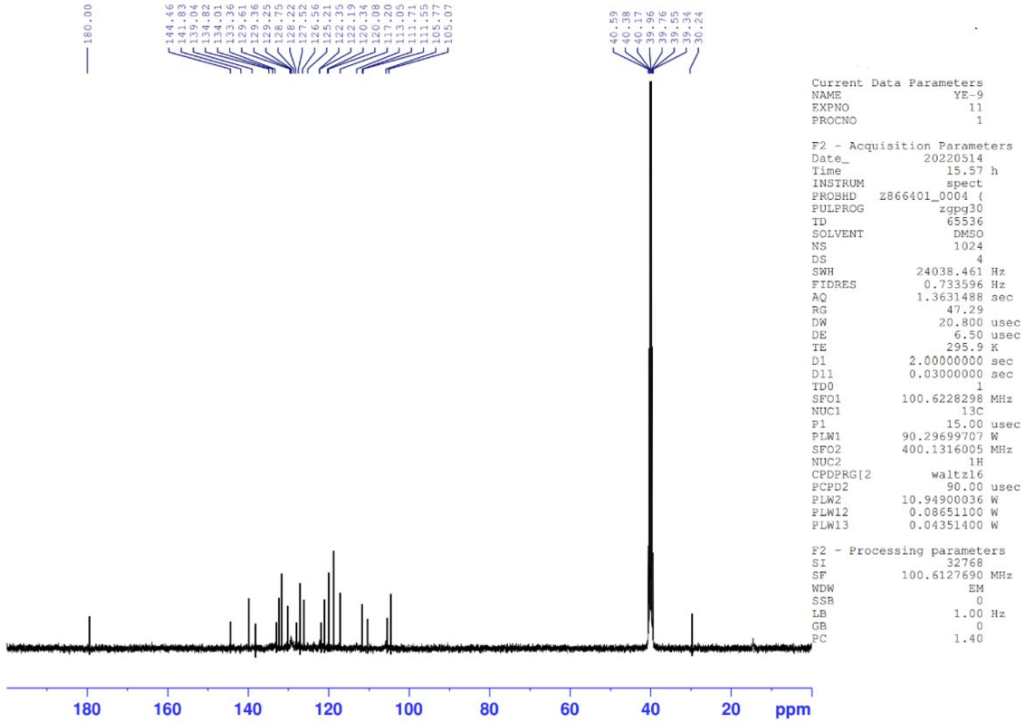

Figure S23. ¹³C-NMR spectrum of compound 6h

Formula Predictor Report - YE-9_158.lcd Page 1 of 1

Data File: C:\LabSolutions\Data\Analiz\uac\YE-9_158.lcd

Elmt	Val.	Min	Max	Elmt	Val.	Min	Max	Elmt	Val.	Min	Max	Elmt	Val.	Min	Max	Use Adduct
H	1	9	18	O	2	1	1	S	2	1	1	Ru	2	0	0	H
C	4	7	37	F	1	0	2	Cl	1	0	0	Pd	2	0	0	Na
N	3	6	6	P	3	0	0	Br	1	0	0	I	3	0	0	NH4

Error Margin (ppm):	5	DBE Range:	5.0 - 30.0	Electron Ions:	both
HC Ratio:	unlimited	Apply N Rule:	yes	Use MSn Info:	yes
Max Isotopes:	3	Isotope RI (%):	1.00	Isotope Res:	9000
MSn Iso RI (%):	10.00	MSn Logic Mode:	AND	Max Results:	50

Event#: 1 MS(E+) Ret. Time : 2.827 -> 2.827 Scan# : 425 -> 425

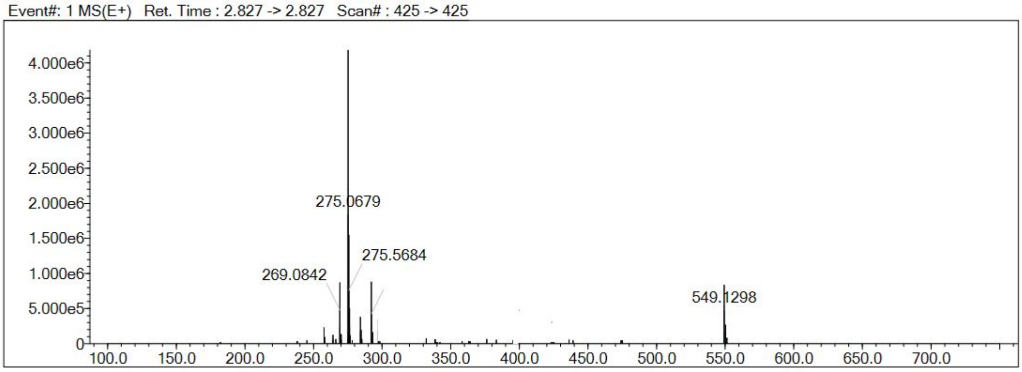

Measured region for 275.0679 m/z

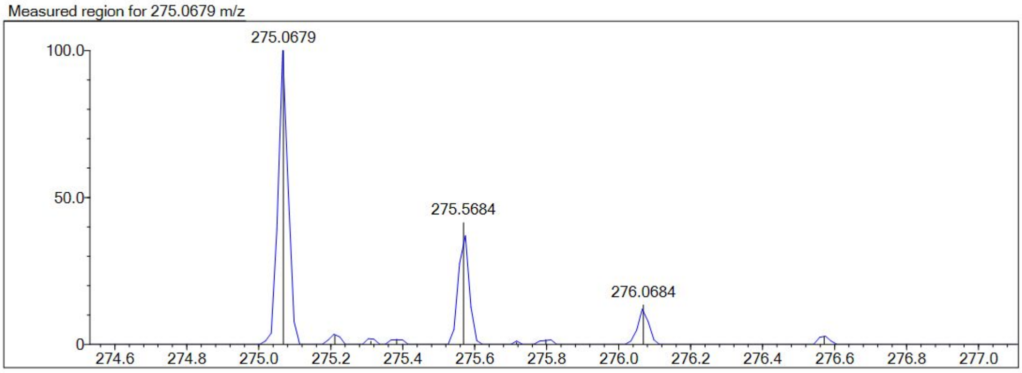

C30 H18 N6 O F2 S [M+2H]2+ : Predicted region for 275.0688 m/z

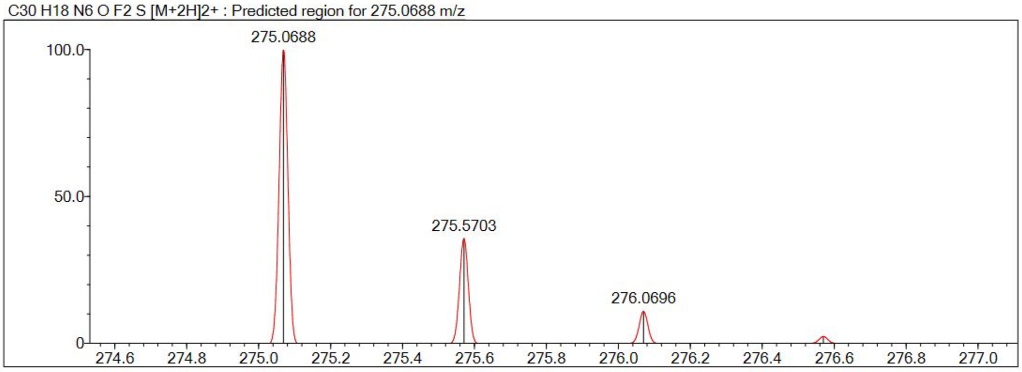

Rank	Score	Formula (M)	Ion	Meas. m/z	Pred. m/z	Df. (mDa)	Df. (ppm)	Iso	DBE
1	79.72	C30 H18 N6 O F2 S	[M+2H]2+	275.0679	275.0688	-0.9	-3.27	84.51	24.0

Figure S24. Mass spectrum of compound 6h

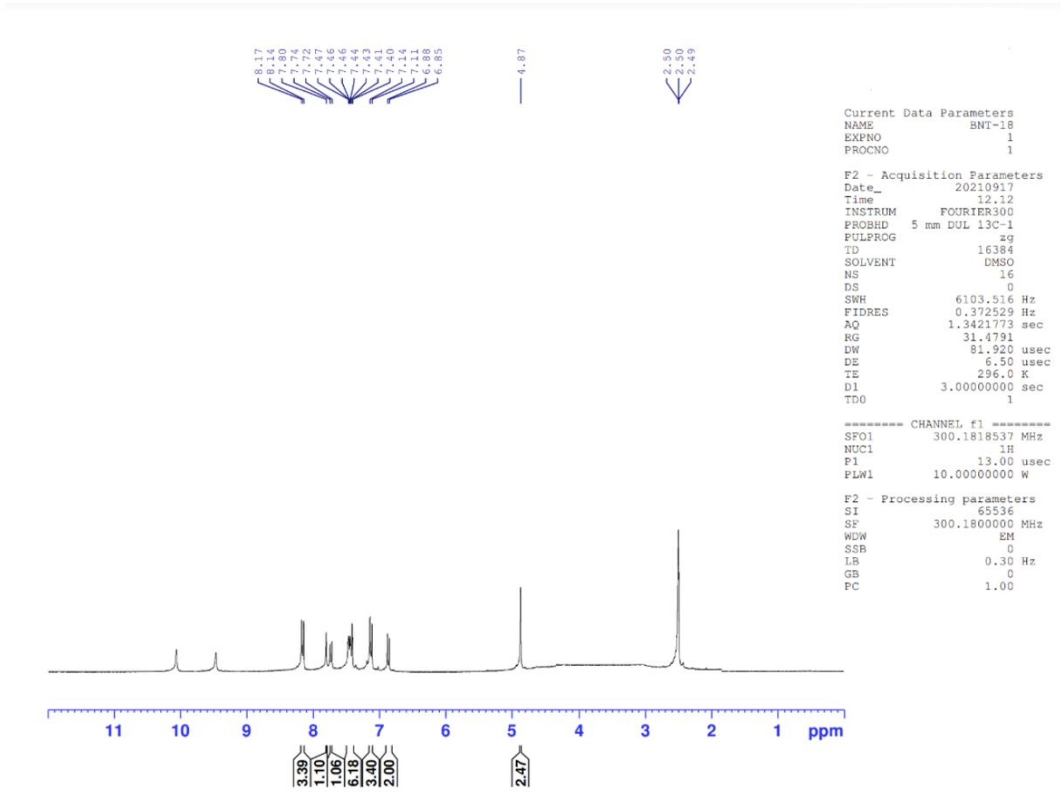

Figure S25. ^{1}H-NMR spectrum of compound 6i

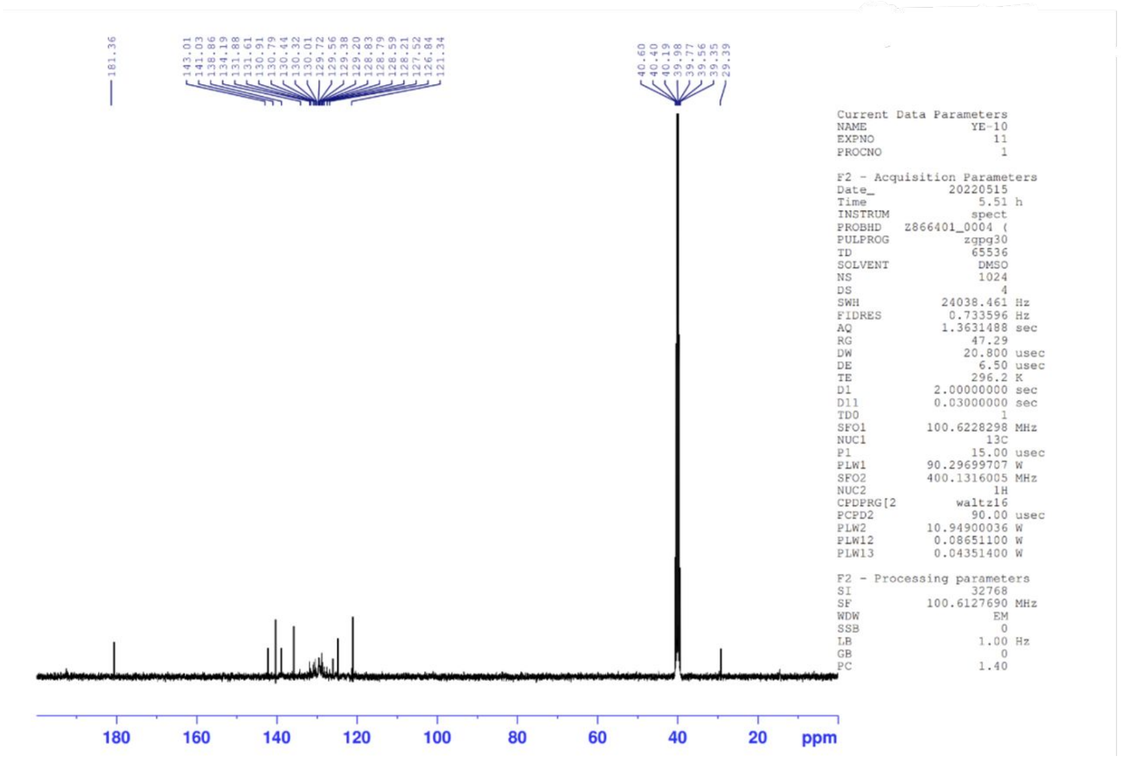

Figure S26. ^{13}C-NMR spectrum of compound 6i

Data File: C:\LabSolutions\Data\Analiz\uac\YE-10_159.lcd

Elmt	Val.	Min	Max	Elmt	Val.	Min	Max	Elmt	Val.	Min	Max	Elmt	Val.	Min	Max	Use Adduct
H	1	9	20	O	2	1	1	S	2	1	1	Ru	2	0	0	H
C	4	7	37	F	1	0	0	Cl	1	1	1	Pd	2	0	0	Na
N	3	6	6	P	3	0	0	Br	1	0	0	I	3	0	0	NH4

Error Margin (ppm):	5	DBE Range:	5.0 - 30.0	Electron Ions:	both
HC Ratio:	unlimited	Apply N Rule:	yes	Use MSn Info:	yes
Max Isotopes:	3	Isotope RI (%):	1.00	Isotope Res:	9000
MSn Iso RI (%):	10.00	MSn Logic Mode:	AND	Max Results:	50

Event#: 1 MS(E+) Ret. Time : 3.000 -> 3.000 Scan# : 451 -> 451

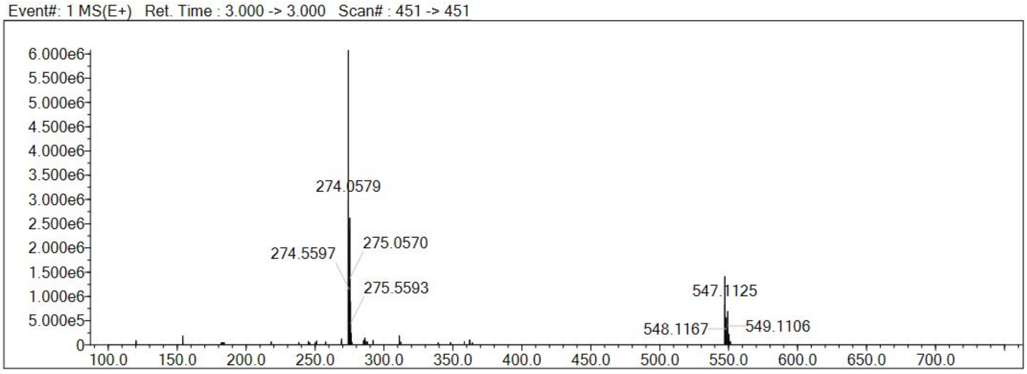

Measured region for 274.0579 m/z

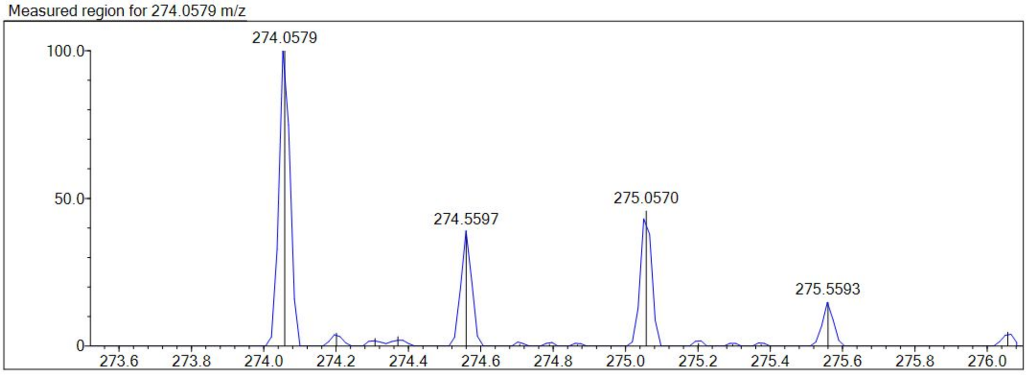

C30 H19 N6 O S Cl [M+2H]2+ : Predicted region for 274.0588 m/z

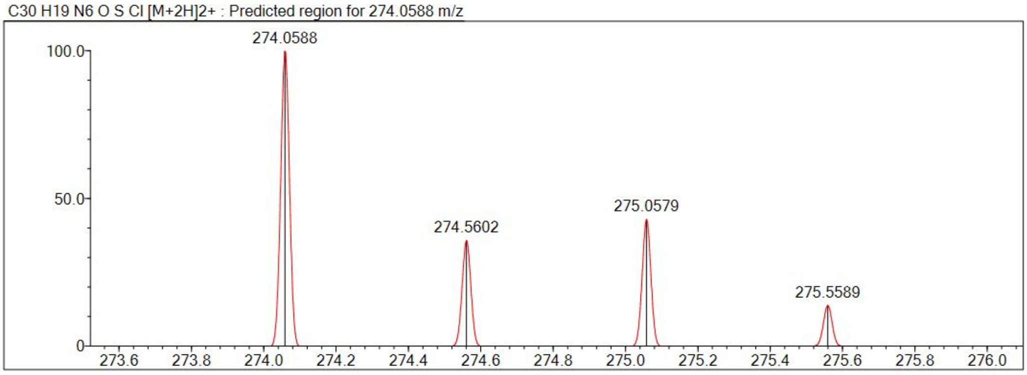

Rank	Score	Formula (M)	Ion	Meas. m/z	Pred. m/z	Df. (mDa)	Df. (ppm)	Iso	DBE
1	75.46	C30 H19 N6 O S Cl	[M+2H]2+	274.0579	274.0588	-0.9	-3.28	80.02	24.0

Figure S27. Mass spectrum of compound 6i

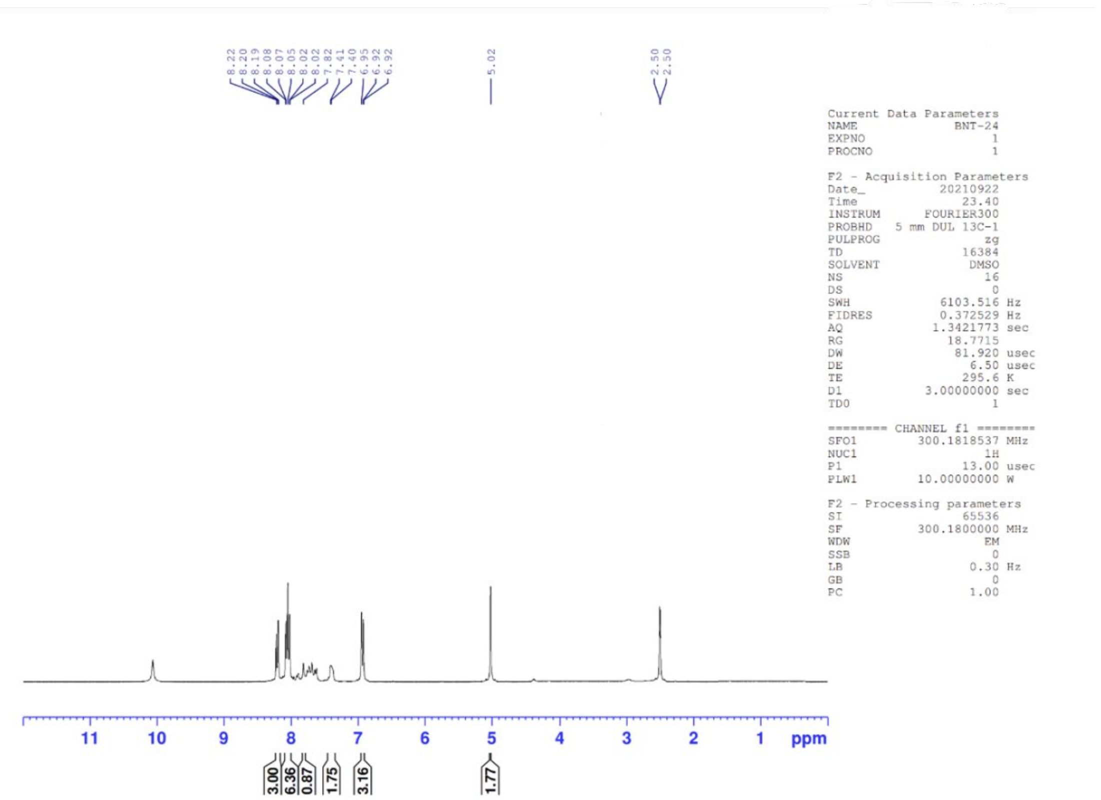

Figure S28. ^{1}H-NMR spectrum of compound 6j

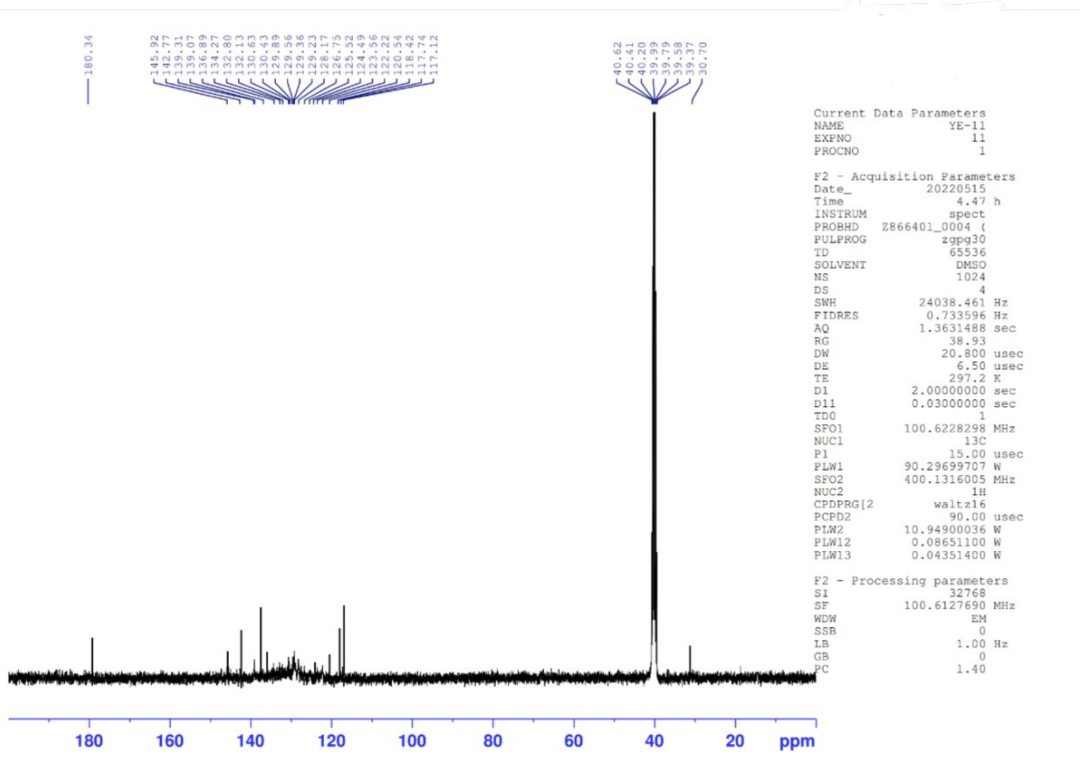

Figure S29. ^{13}C-NMR spectrum of compound 6j

Data File: C:\LabSolutions\Data\Analiz\uac\YE-11_160.lcd

Elmt	Val.	Min	Max	Elmt	Val.	Min	Max	Elmt	Val.	Min	Max	Elmt	Val.	Min	Max	Use Adduct
H	1	9	20	O	2	1	1	S	2	1	1	Ru	2	0	0	H
C	4	7	37	F	1	0	0	Cl	1	2	2	Pd	2	0	0	Na
N	3	6	6	P	3	0	0	Br	1	0	0	I	3	0	0	NH4

Error Margin (ppm): 5
HC Ratio: unlimited
Max Isotopes: 3
MSn Iso RI (%): 10.00

DBE Range: 5.0 - 30.0
Apply N Rule: yes
Isotope RI (%): 1.00
MSn Logic Mode: AND

Electron Ions: both
Use MSn Info: yes
Isotope Res: 9000
Max Results: 50

Event#: 1 MS(E+) Ret. Time : 3.640 -> 3.640 Scan# : 547 -> 547

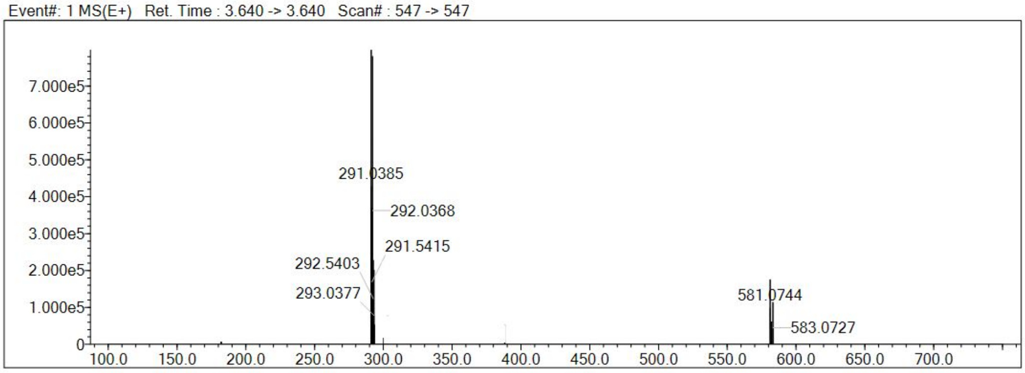

Measured region for 291.0385 m/z

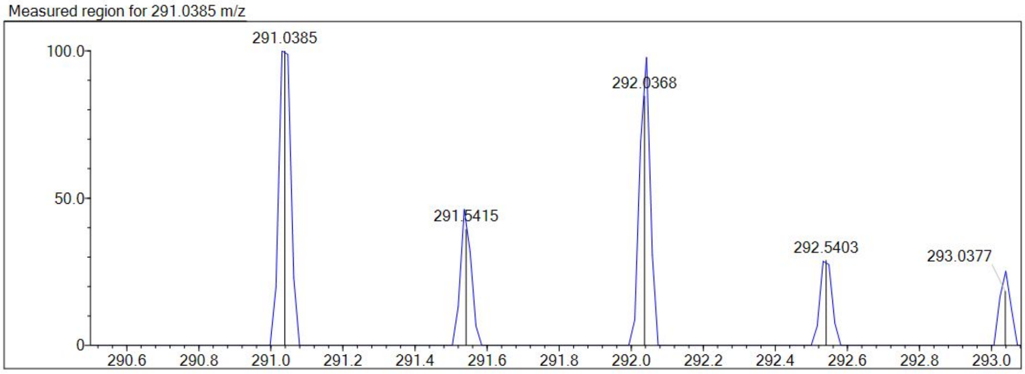

C30 H18 N6 O S Cl2 [M+2H]2+ : Predicted region for 291.0393 m/z

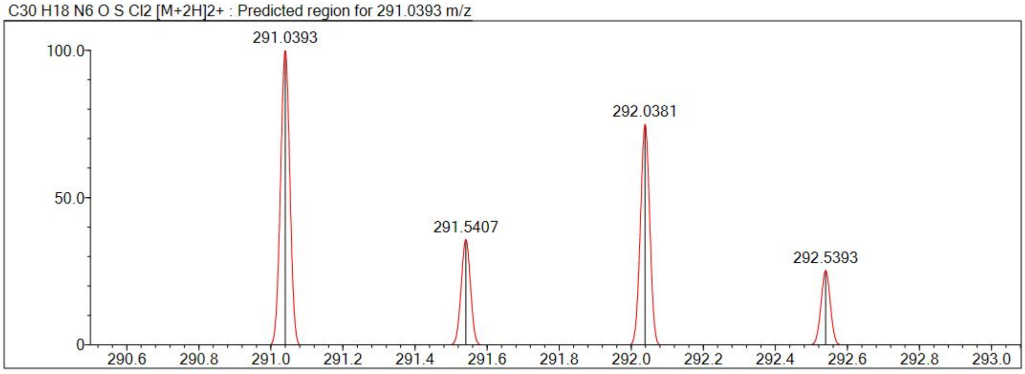

Rank	Score	Formula (M)	Ion	Meas. m/z	Pred. m/z	Df. (mDa)	Df. (ppm)	Iso	DBE
1	64.89	C30 H18 N6 O S Cl2	[M+2H]2+	291.0385	291.0393	-0.8	-2.75	67.86	24.0

Figure S30. Mass spectrum of compound 6j

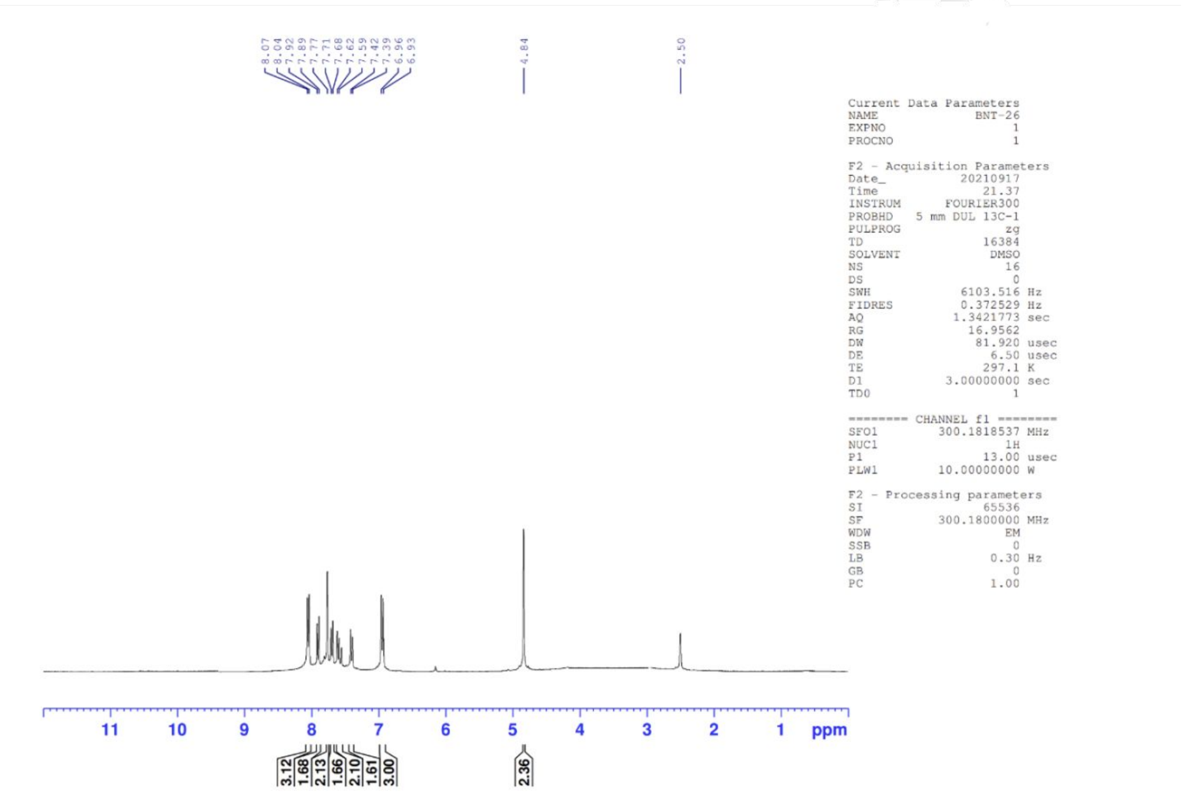

Figure S31. ^{1}H-NMR spectrum of compound 6k

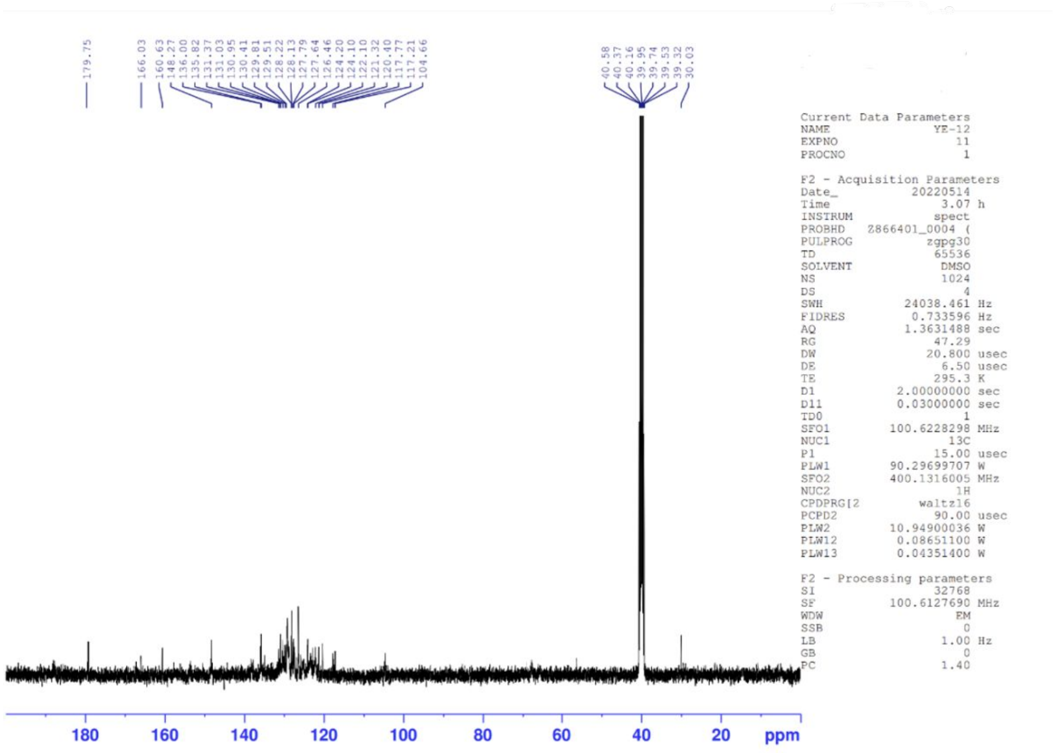

Figure S32. ^{13}C-NMR spectrum of compound 6k

Formula Predictor Report - YE-12_161.lcd	Page 1 of 1

Data File: C:\LabSolutions\Data\Analiz\uac\YE-12_161.lcd

Elmt	Val.	Min	Max	Elmt	Val.	Min	Max	Elmt	Val.	Min	Max	Elmt	Val.	Min	Max	Use Adduct
H	1	9	20	O	2	3	3	S	2	1	1	Ru	2	0	0	H
C	4	7	37	F	1	0	0	Cl	1	0	0	Pd	2	0	0	Na
N	3	7	7	P	3	0	0	Br	1	0	0	I	3	0	0	NH4

Error Margin (ppm):	5	DBE Range:	5.0 - 30.0	Electron Ions:	both
HC Ratio:	unlimited	Apply N Rule:	yes	Use MSn Info:	yes
Max Isotopes:	3	Isotope RI (%):	1.00	Isotope Res:	9000
MSn Iso RI (%):	10.00	MSn Logic Mode:	AND	Max Results:	50

Event#: 1 MS(E+)   Ret. Time : 2.760   Scan# : 415

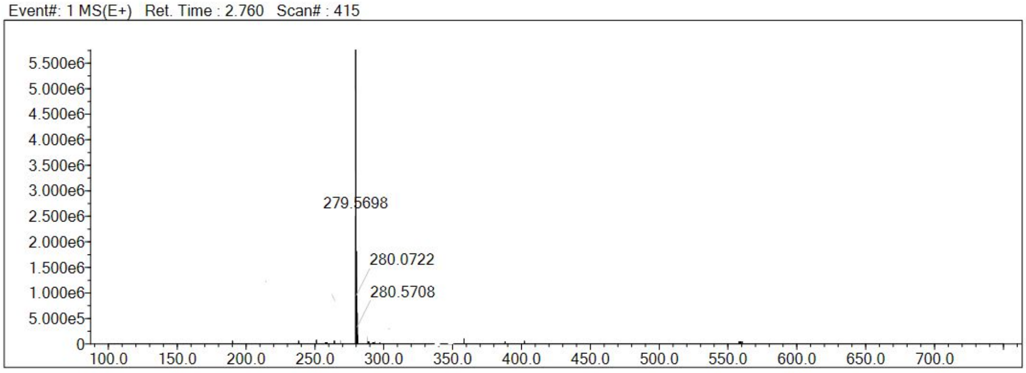

Measured region for 279.5698 m/z

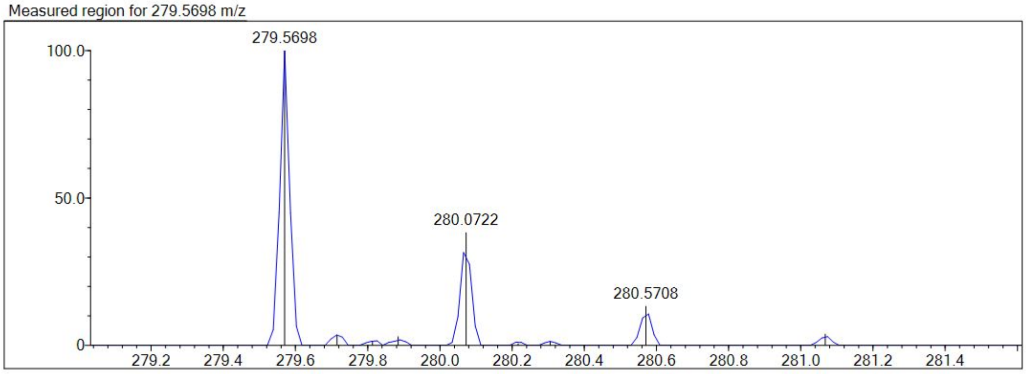

C30 H19 N7 O3 S [M+2H]2+ : Predicted region for 279.5708 m/z

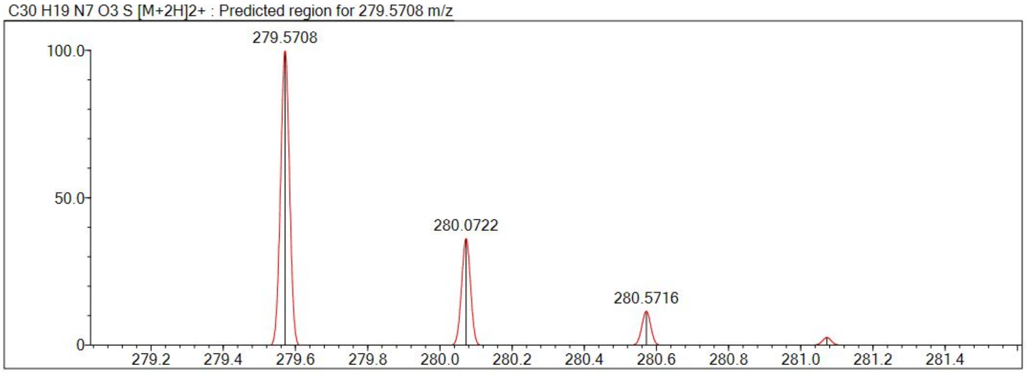

Rank	Score	Formula (M)	Ion	Meas. m/z	Pred. m/z	Df. (mDa)	Df. (ppm)	Iso	DBE
1	78.67	C30 H19 N7 O3 S	[M+2H]2+	279.5698	279.5708	-1.0	-3.58	84.09	25.0

Figure S33. Mass spectrum of compound 6k

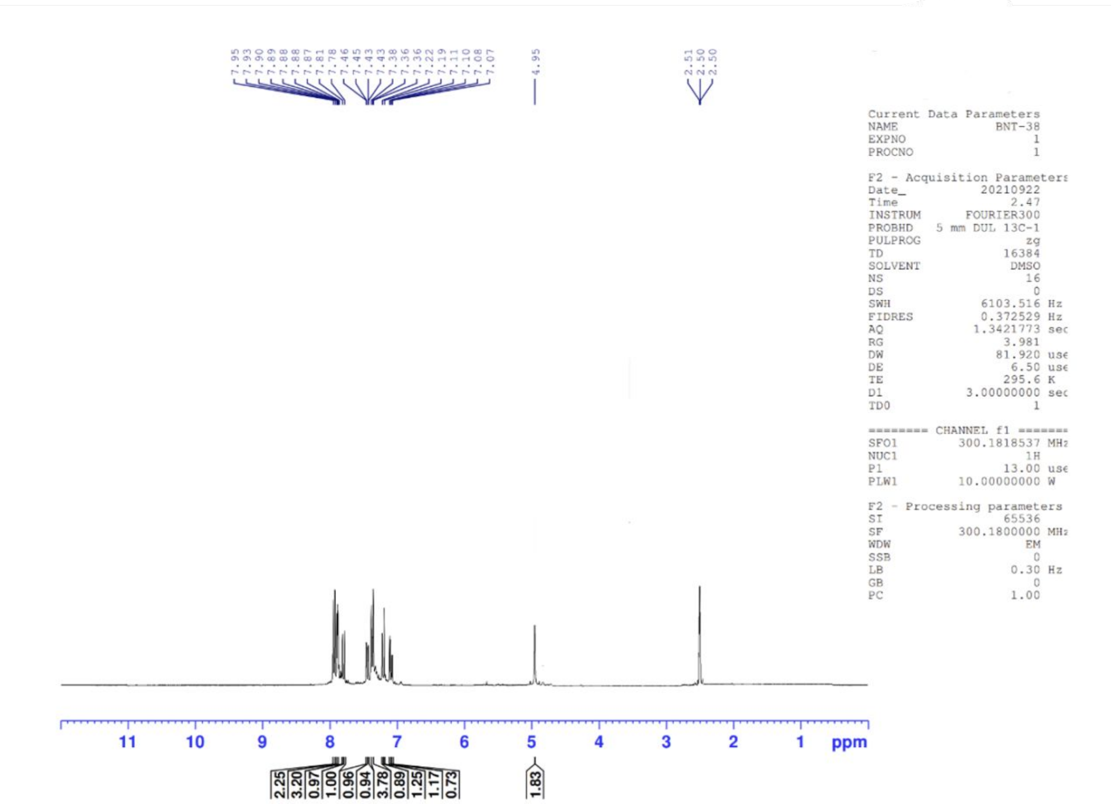

Figure S34. ^{1}H-NMR spectrum of compound 6l

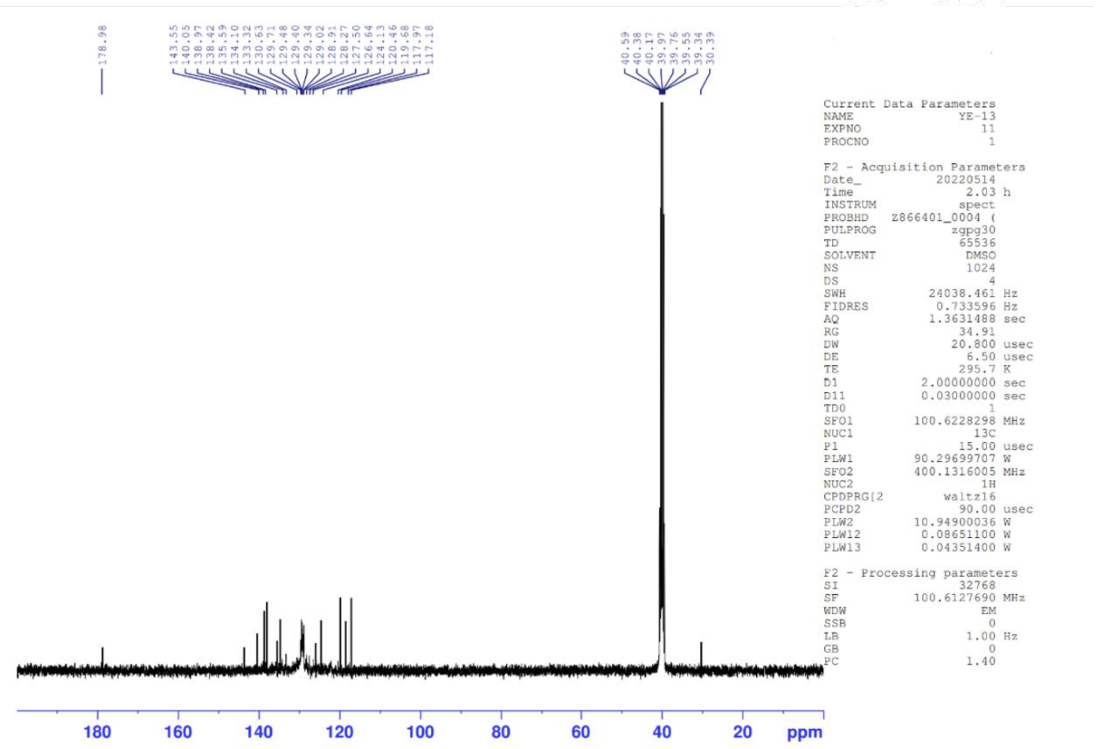

Figure S35. ^{13}C-NMR spectrum of compound 6l

Formula Predictor Report - YE-13_162.lcd Page 1 of 1

Data File: C:\LabSolutions\Data\Analiz\uac\YE-13_162.lcd

Elmt	Val.	Min	Max	Elmt	Val.	Min	Max	Elmt	Val.	Min	Max	Elmt	Val.	Min	Max	Use Adduct
H	1	9	20	O	2	1	1	S	2	1	1	Ru	2	0	0	H
C	4	7	37	F	1	0	0	Cl	1	0	0	Pd	2	0	0	Na
N	3	6	6	P	3	0	0	Br	1	0	0	I	3	0	0	NH4

Error Margin (ppm):	5	DBE Range:	5.0 - 30.0	Electron Ions:	both
HC Ratio:	unlimited	Apply N Rule:	yes	Use MSn Info:	yes
Max Isotopes:	3	Isotope RI (%):	1.00	Isotope Res:	9000
MSn Iso RI (%):	10.00	MSn Logic Mode:	AND	Max Results:	50

Event#: 1 MS(E+) Ret. Time : 2.373 Scan# : 357

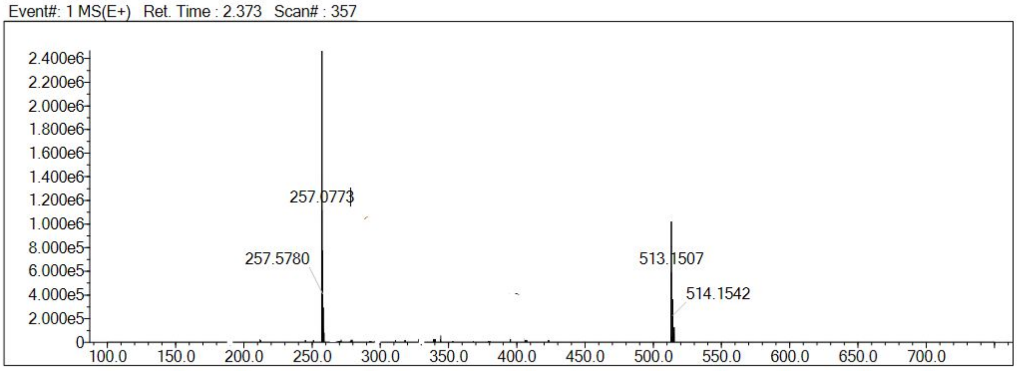

Measured region for 257.0773 m/z

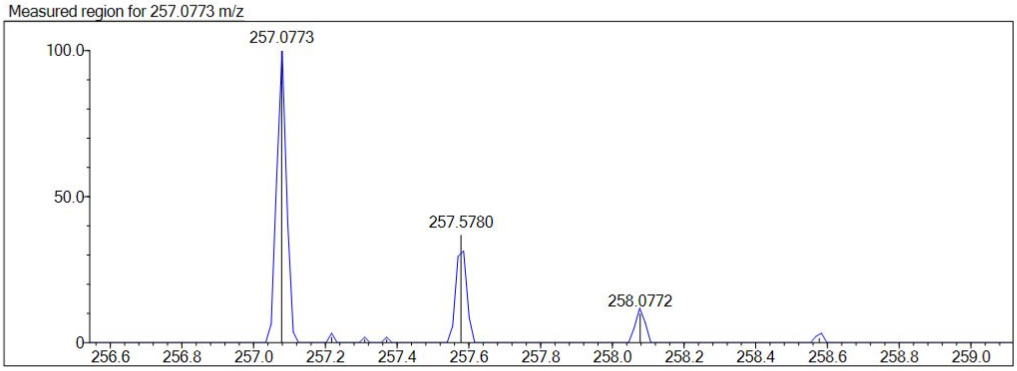

C30 H20 N6 O S [M+2H]2+ : Predicted region for 257.0782 m/z

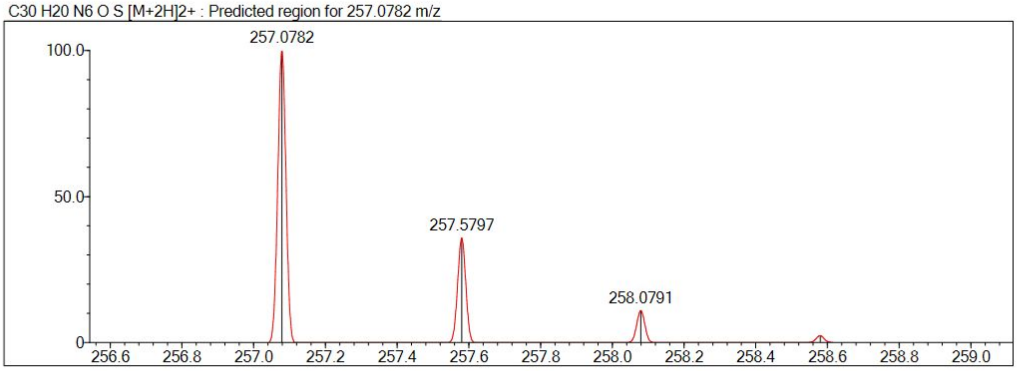

Rank	Score	Formula (M)	Ion	Meas. m/z	Pred. m/z	Df. (mDa)	Df. (ppm)	Iso	DBE
1	81.78	C30 H20 N6 O S	[M+2H]2+	257.0773	257.0782	-0.9	-3.50	87.23	24.0

Figure S36. Mass spectrum of compound 6l
